# Programmable DNA-binding proteins from *Burkholderia* provide a fresh perspective on the TALE-like repeat domain

**DOI:** 10.1093/nar/gku329

**Published:** 2014-05-03

**Authors:** Orlando de Lange, Christina Wolf, Jörn Dietze, Janett Elsaesser, Robert Morbitzer, Thomas Lahaye

**Affiliations:** Genetics, Department of Biology I, Ludwig-Maximilians-University Munich, Martinsried, Bavaria, 82152, Germany

## Abstract

The tandem repeats of transcription activator like effectors (TALEs) mediate sequence-specific DNA binding using a simple code. Naturally, TALEs are injected by *Xanthomonas* bacteria into plant cells to manipulate the host transcriptome. In the laboratory TALE DNA binding domains are reprogrammed and used to target a fused functional domain to a genomic locus of choice. Research into the natural diversity of TALE-like proteins may provide resources for the further improvement of current TALE technology. Here we describe TALE-like proteins from the endosymbiotic bacterium *Burkholderia rhizoxinica*, termed Bat proteins. Bat repeat domains mediate sequence-specific DNA binding with the same code as TALEs, despite less than 40% sequence identity. We show that Bat proteins can be adapted for use as transcription factors and nucleases and that sequence preferences can be reprogrammed. Unlike TALEs, the core repeats of each Bat protein are highly polymorphic. This feature allowed us to explore alternative strategies for the design of custom Bat repeat arrays, providing novel insights into the functional relevance of non-RVD residues. The Bat proteins offer fertile grounds for research into the creation of improved programmable DNA-binding proteins and comparative insights into TALE-like evolution.

## INTRODUCTION

When the DNA binding code of transcription activator like effectors (TALEs) was published in 2009 (1,2), a doorway was opened for researchers to build custom DNA-binding proteins. In nature, TALE proteins are injected by members of the plant pathogenic bacterial genus *Xanthomonas* into host cells. They act as eukaryotic transcription factors, inducing expression of targeted host genes that promote bacterial disease. This relies on a set of functional domains within the protein (3). Upon injection into host cells, nuclear localisation signals (NLSs) target TALEs to the plant nucleus. There the central domain of the protein, composed of tandem-arranged repeats, mediates sequence-specific binding to the promoters of target genes. A C-terminal transcriptional activation domain (AD) mediates promoter activation. The unique repeat array, mediating interaction of TALEs with DNA, has received great attention in the past years. Functional arrays are typically composed of 10–30 repeats, each 33–35 amino acids in length (3). Within repeats, variation is almost exclusively limited to positions 12 and 13, termed the repeat variable di-residue (RVD; 2). One repeat binds one base with specificity determined by the RVD. The TALE code refers to this 1-to-1 correlation and the base preferences defined by the distinct RVDs, providing a simple guide for users. By modifying repeat number and RVD composition users can design custom TALE repeat arrays that target nucleotide sequences of desired length and base composition.

Since the inter-repeat polymorphisms of TALE repeat arrays are almost solely limited to the RVDs, reprogramming of base specificity is straightforward. As a consequence of the almost identical amino acid composition, each TALE repeat forms a near identical structure irrespective of its position in the array (4,5). Accordingly, each repeat is competent to make almost exactly the same inter-repeat interactions regardless of the residues occupying the RVD positions (4,5). Thus, changes to repeat number or position do not perturb the network of inter-repeat interactions that stabilize the superhelical structure formed by tandem-arranged repeats. This allows each repeat to be treated as a functionally independent module and isolates the RVD as the only position within the repeat of interest to the user.

Functional domains of choice can be fused to the TALE DNA binding domain and targeted to a predefined DNA sequence. By now TALE-activators, repressors and nucleases have been used extensively (6) and more recently TALE fusions mediating targeted epigenetic modifications have also been described (7–9).

Work on the TALE-like proteins of *Ralstonia solanacearum*, termed RipTALs, has revealed that they too act as eukaryotic transcription factors and that RipTAL target specificity is linked to RVDs as in TALEs (10). Comparative analysis of TALE and RipTAL repeat arrays also revealed functional differences, due to non-RVD polymorphisms, which could be used to improve custom TALE repeat arrays. Considering the ever-increasing use of TALEs across fundamental and applied biology, it seems sensible to further explore the natural diversity of this protein class in order to identify new functional features of benefit to users.

*Burkholderia rhizoxinica* is an obligate endosymbiotic bacterium of the fungal plant pathogen *Rhizopus microsporus* (11). The genome of *B. rhizoxinica* strain HKI 0454 has been sequenced (12) and among the predicted proteins are three with similarity to TALEs that we have termed Bat (*Burkholderia* TALE-like) proteins. The gene encoding the predicted Bat1 protein (Uniprot E5AV36, GenBank RBRH_01844) is located on megaplasmid pBRH01 while the predicted Bat2 (Uniprot E5AW45, GenBank RBRH_01776) and Bat3 proteins (Uniprot E5AW43, GenBank RBRH_01777) are encoded on neighbouring, non-overlapping open reading frames within plasmid pBRH02. Evidence for DNA binding activity and use as a programmable DNA binding domain has been demonstrated recently for Bat1 (alternatively designated BurrH; 13,14). We investigated DNA binding properties of the three Bat proteins, showing that Bat2 as well as Bat1 binds DNA with the same code as TALEs. We quantified the interaction of Bat1 with its predicted target DNA bearing the four possible zero bases and found that, unlike TALEs and RipTALs, Bat1 has no sequence preference at this position. Bat proteins share limited sequence identity with TALEs and also show greater inter-repeat diversity than TALEs or the recently described RipTALs. However, alignments between repeats of these different proteins reveal a core set of conserved residues that might be of use to identify further members of this class. We show that the Bat proteins can be used as modular DNA binding domains to mediate targeted transcriptional activation or site-directed DNA cleavage. However, in contrast to TALEs, no two repeats of any Bat proteins are identical, with inter-repeat similarity dropping below 50% in some cases. Because of this alternative approaches are possible for the customisation of the DNA binding repeats. We explored two options: exchanging whole repeats along with their RVDs or exchanging RVDs only. We found that while one strategy seems preferable, both are viable. In the process we gained evidence to suggest that polymorphisms at non-RVD positions affect binding domain function. Our observations suggest that the Bat proteins may offer a more compact alternative to the TALE platform for programmable DNA binding.

## MATERIALS AND METHODS

### Assembly of Bat1 and TALE expression constructs

Genes encoding the three Bat proteins were synthesized with *Escherichia coli* codon usage (GenScript) in separate BsaI-site flanked subunits (Supplementary Figure S4). For *E. coli* protein production, these modules were assembled via BsaI cut-ligation into a pENTR/D-TOPO (Life Technologies) derivative bearing BsaI sites (overlaps *CACC-AAGG*) within the LR recombination sites, created using primers listed in Supplementary Table S2. The genes were then transferred into pDEST-17 (Life Technologies). For human cell transfection and *in vitro* cleavage assays *bat* encoding modules were assembled along with BsaI-site-flanked modules encoding HA-NLS and NLS-3xFLAG-VP64 AD domains (acBat1, human cell reporter) or 3xHA/HA-NLS and HA-FokI (*in vitro* cleavage). Sequences are in Supplementary Figure S5. These were assembled into a modified pVAX vector (Life Technologies) with combined Cytomegalovirus (CMV)/Sp6 promoter and BsaI sites (*AATG-GCTT*), details and sequences for mutational primers given in Supplementary Table S2.

The acBat1 truncation derivatives tested in Figure 5 were carried out using polymerase chain reaction (PCR) on the individual synthesized blocks of Bat1 prior to assembly, using the primers listed in Supplementary Table S2. To create the acBat1 derivatives tested in Figure 6 modified assembly blocks were synthesized with the same codon usage as wild-type *acbat1* (GenScript). To create the *pSOX2* targeted dBats tested in Figure 7, a DNA fragment encoding the N- and C-terminal non-core-repeat sections of Bat1 was synthesized with the same codon usage as wild-type *acbat1* (GenScript; Supplementary Figure S11) and assembled into the pVAX vector along with HA-NLS, NLS-3xFLAG-VP64 constructs. The repeats were ordered as two blocks for each dBat (Supplementary Figure S11) and added into the expression vector between N- and C-terminally encoding regions via BpiI cut-ligation.

**Figure 1.**
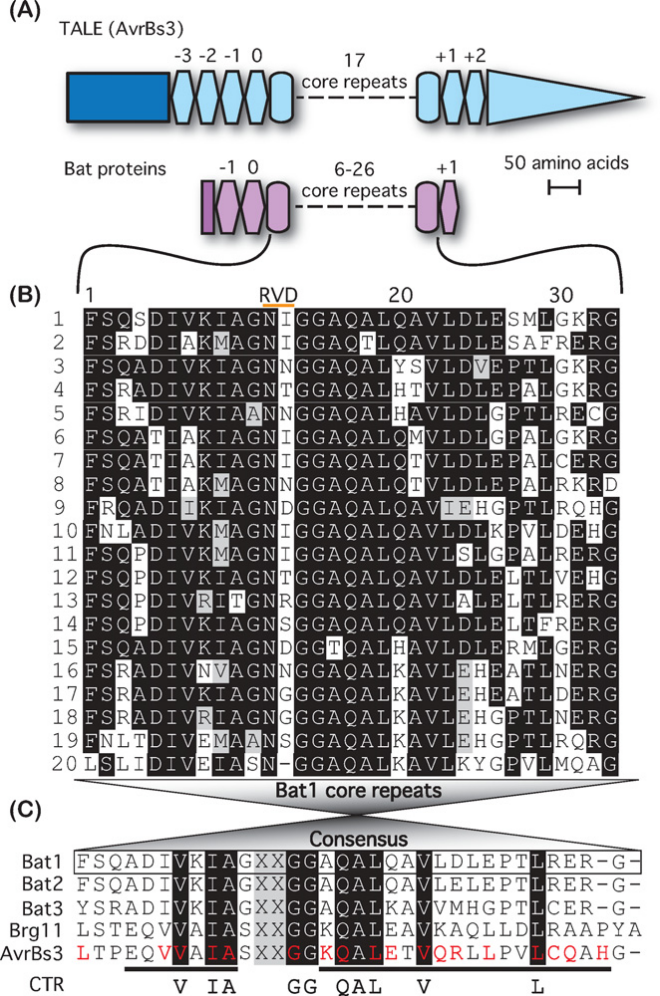
Sequence-based comparison of TALE-like proteins. (**A**) Comparison of TALE (AvrBs3) and Bat architecture. The lengths of all domains are drawn to the indicated scale, except the dashes representing core repeats. TALE domains are shown in blue and Bat domains in purple. Rectangles indicate the N-terminal non-repetitive domain of each while a triangle indicates the non-repetitive C-terminal domain of TALEs including the transcriptional AD. Ovals represent core repeats, hexagons represent cryptic repeats (repeat number is indicated above). (**B**) Alignment of Bat1 core repeats, generated with Clustal Omega and Boxshade. Repeats are shown in order of appearance in the polypeptide. Repeat numbers are given on the left and positions within the repeat, including the RVD (indicated by an orange bar) above. (**C**) A consensus repeat generated from this alignment is compared to similarly generated consensus repeats from Bat2, Bat3, Brg11 (RipTAL) and AvrBs3 (TALE). From these a set of 10 hyper-conserved residues termed the consensus TALE-like repeat (CTR) was generated. The RVD positions are excluded from this. Repeat residues previously identified as involved in stabilising intra-molecular interactions from structural studies in TALEs (4) are highlighted with red lettering in the AvrBs3 consensus repeat, while the residues forming the first and second alpha helices (4) are underlined.

**Figure 2.**
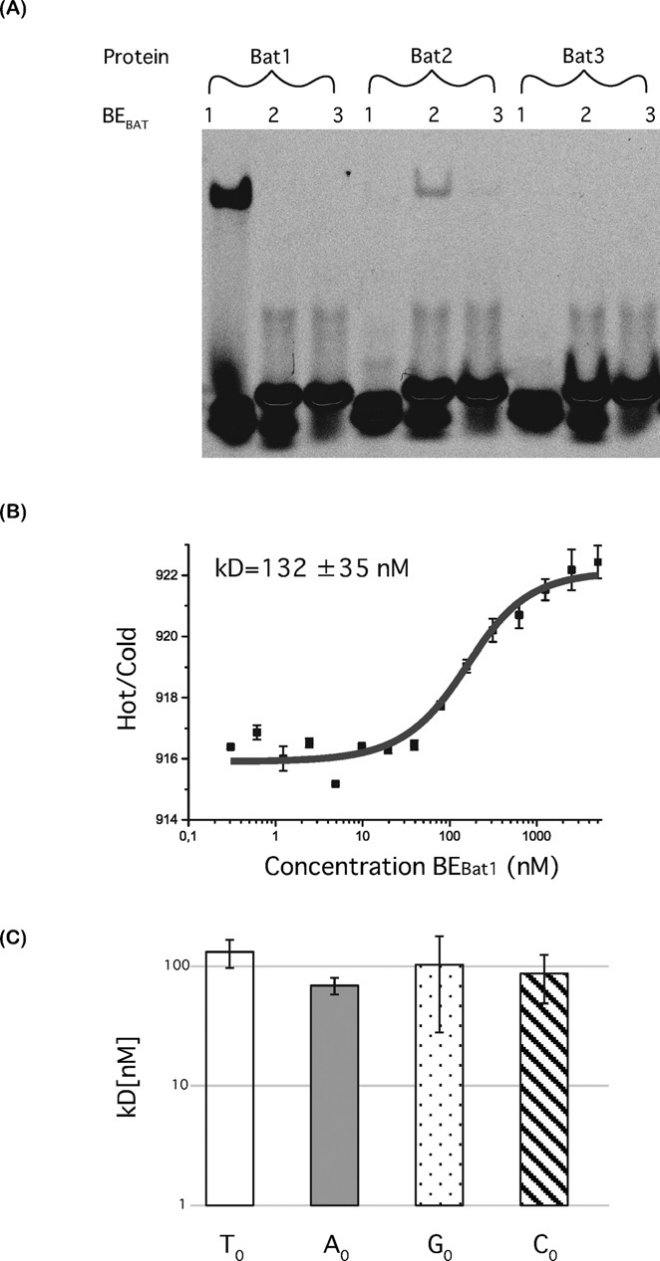
*In vitro* interaction studies of Bat proteins with predicted DNA targets. (**A**) Electrophoretic mobility shift assays were carried out for Bat1, 2 and 3 using 5’Cy5 labelled double-stranded DNA, bearing target sequences deduced from the TALE code. Each protein (100 nM) was tested against each target DNA (10 nM). Cy5 fluorescence was visualized after running through a native polyacrylamide gel. A shifted band, running slower on the gel, indicates the protein–DNA complex. (**B**) The interaction between Bat1 and its target (BE_Bat1_) was quantified using microscale thermophoresis. The fluorescence ratio over the thermophoretic jump is shown on the y-axis against DNA concentration. Standard deviation for four repetitions is indicated. Measurements were made with 40% LED and 20% laser power. The dark grey line indicates the Kd fit. (**C**) This was repeated for BE _Bat1_ derivatives bearing A (grey bar), C (filled stripes) or G (spotted) at the zero position. The Kd was calculated in each case and is shown compared to that with BE_Bat1_ (T_0_, empty bar).

**Figure 3.**
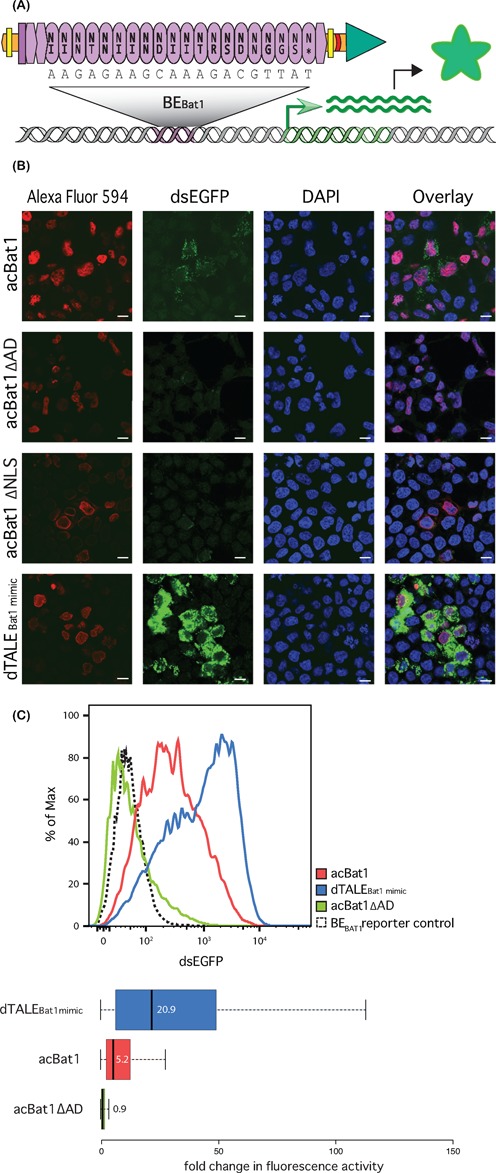
A Bat1 derived transcriptional activator (acBat1) is functional in a human cell reporter assay. (**A**) Schematic drawing showing the domain composition of acBat1. NLSs (yellow bars), a 3xFLAG tag (red crescent line) and a VP64 AD (green triangle) were fused onto Bat1 (purple) via flexible linkers (orange). This was introduced into HEK293T cells via transfection alongside a DNA reporter (grey) bearing BE _Bat1_ (purple) upstream of a dsEGFP coding sequence (green). Transcriptional activation of the reporter (green arrow) follows binding to BE _Bat1_, leading to production of dsEGFP protein (green star). acBat1 is detected via the 3xFLAG epitope with use of an Alexa Fluor 594 labelled secondary antibody. (**B**) Alexa Fluor 594, dsEGFP and DAPI fluorescence are shown for transfected cells. acBat1 is compared to derivatives lacking AD (acBat1ΔAD) or NLSs (acBat1ΔNLSs) and to a dTALE created with the same NLSs and AD and with the same core repeat number and RVD composition as Bat1 (dTALE_Bat1mimic_). The scale bar indicates 10 μm. (**C**) FACS analysis was used to quantify dsEGFP fluorescence for transfected cells expressing acBat1, ΔAD derivative or dTALE_Bat1mimic_ as well as cells transfected with the reporter only. dsEGFP values are shown for the whole population (curves) as well as boxplots showing fold changes in fluorescence intensity compared to the reporter control. Boxplot whiskers represent the 2.5% and 97.5% data limits. Median values are written next to or inside each box plot and shown graphically with thick black lines.

**Figure 4.**
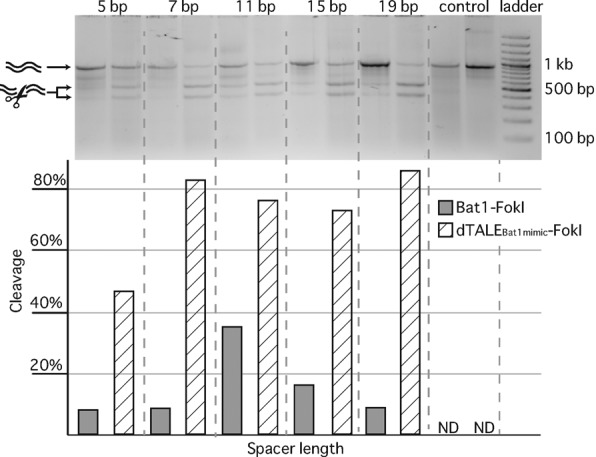
*In vitro* assessment of Bat1-FokI nuclease activity. Bat1- and TALE-FokI fusion proteins were expressed *in vitro* and equal volumes of transcription-translation product were incubated with a purified PCR product bearing two copies of BE_Bat1_ in reverse complement, separated by 5–19 base pairs. A target with a control sequence replacing the Bat1 target boxes was also used. After 3 h incubation at 37°C DNA was purified from the nuclease reactions and run on a 2% agarose gel to discriminate cleaved and uncleaved DNA (indicated with arrows and illustrations on left side). Cleavage efficacy was calculated from the ratio of cleaved to uncleaved DNA band intensities in each lane with ImageJ (14). Full and striped bars indicate activities of the Bat1-FokI and TALEN constructs respectively. ND = none detected.

**Figure 5.**
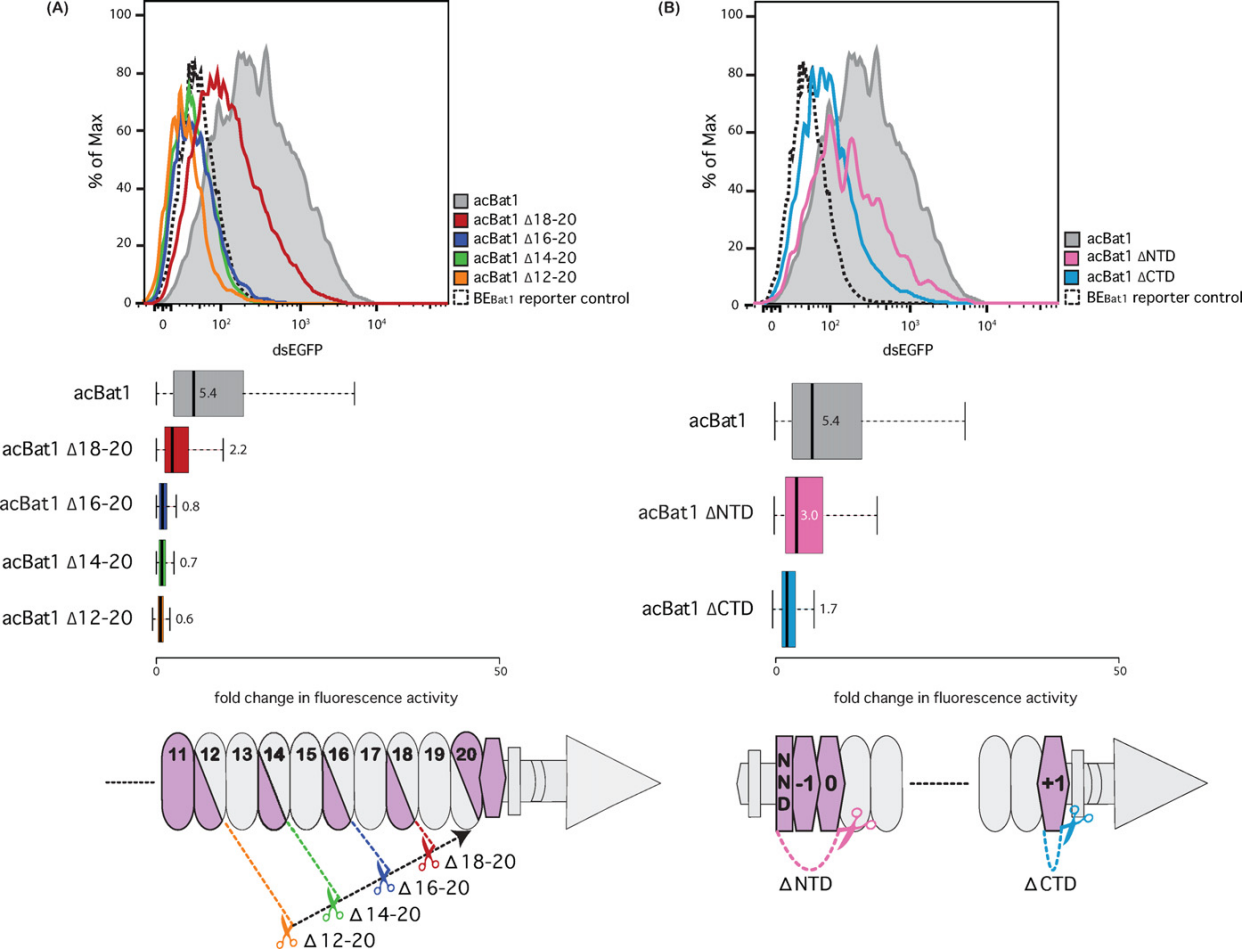
Functional analysis of acBat1 repeat truncations. Tests were carried out as described (Figure 3). Flow cytometry measurements of dsEGFP fluorescence are displayed as population distributions (top) or box plots (centre). Distinct colour codes are used throughout the whole figure and correspond to indicated constructs. Boxplots show fold changes in fluorescence intensity compared to the reporter control with whiskers representing the 2.5% and 97.5% data limits. Median values are written next to or inside each box plot and shown graphically as thick black lines. Cartoon representations of the tested truncations are shown below. Dashed lines with scissors indicate fixed (black) and variable (coloured) truncation points. Bat repeats and fused domains of acBat1 are represented as in Figure 3A. (**A**) Within the repeats grey or purple indicate truncated or retained regions, respectively. (**B**) N- (ΔNTD) or C- (ΔCTD) terminal truncations were tested. NND is the short non-repetitive N-terminal domain at the N-terminus of Bat1.

**Figure 6.**
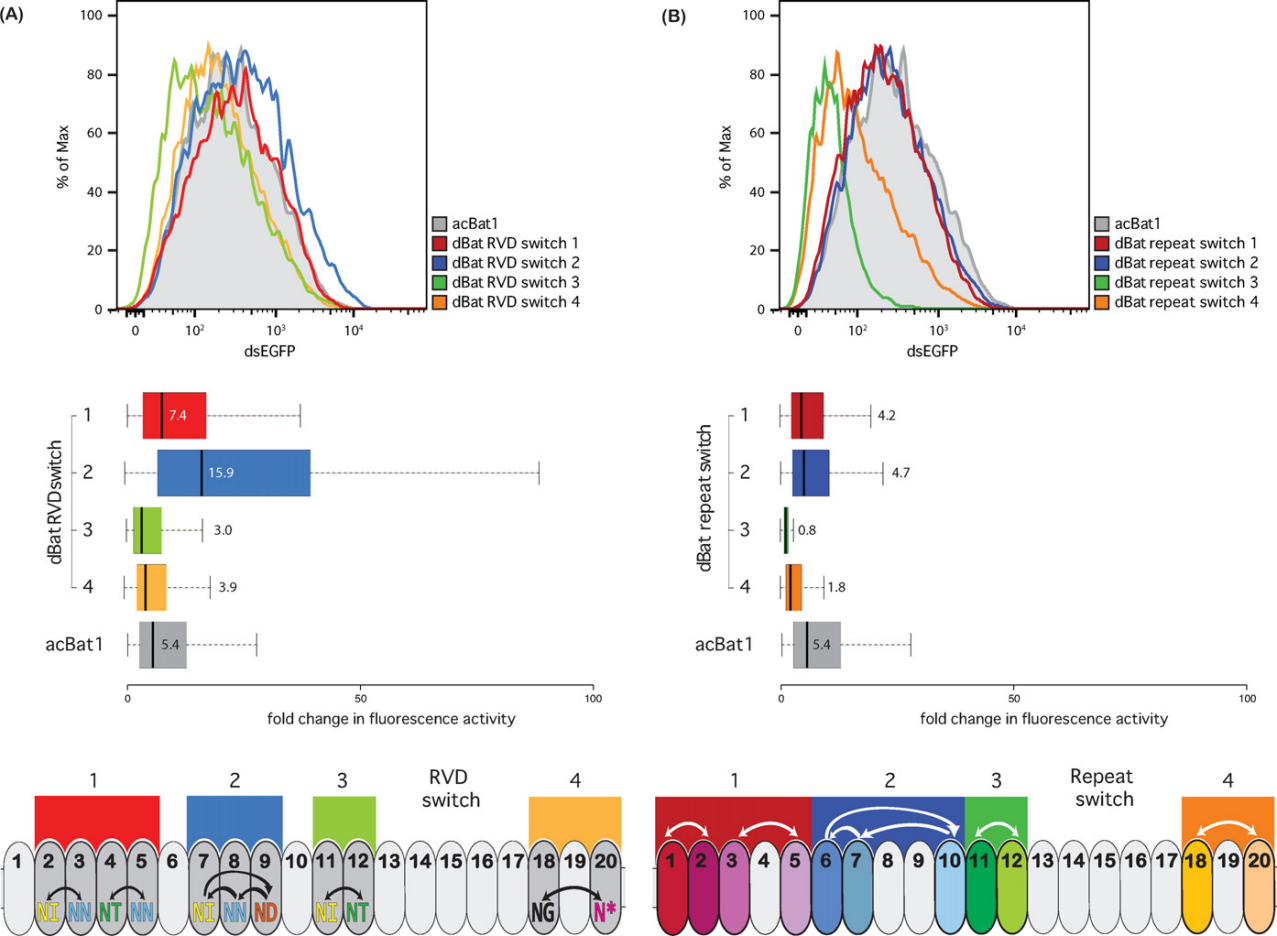
Functional analysis of designer (d)Bat constructs generated by RVD (**A**) or repeat switch (**B**). dBats were tested using flow cytometry with a transcriptional activation reporter as described (Figure 3). dsEGFP fluorescence values are displayed as population distributions (top) or boxplots (centre). dsEGFP values are normalized to the reporter only control (Supplementary Figure S13), which was BE_Bat1_ for all constructs except RVD switch 1 and 2 (Supplementary Figure S6). Boxplots show fold changes in fluorescence intensity compared to the reporter control with whiskers representing the 2.5% and 97.5% data limits. Median values are written next to or inside each box plot and shown graphically as thick black lines. dBat design is outlined below in each case. Coloured boxes indicate the repeats (ovals) modified in a given dBat. In the case of the RVD switch (A) modified repeats are highlighted with darker grey. RVDs are shown and colour coded by type. Arrows indicate the rearrangement of RVDs between repeats. In the case of the repeat switch (B) repeats are coloured to indicate that each has a unique set of non-RVD residues. Arrows indicate movement of whole repeats within the array.

**Figure 7.**
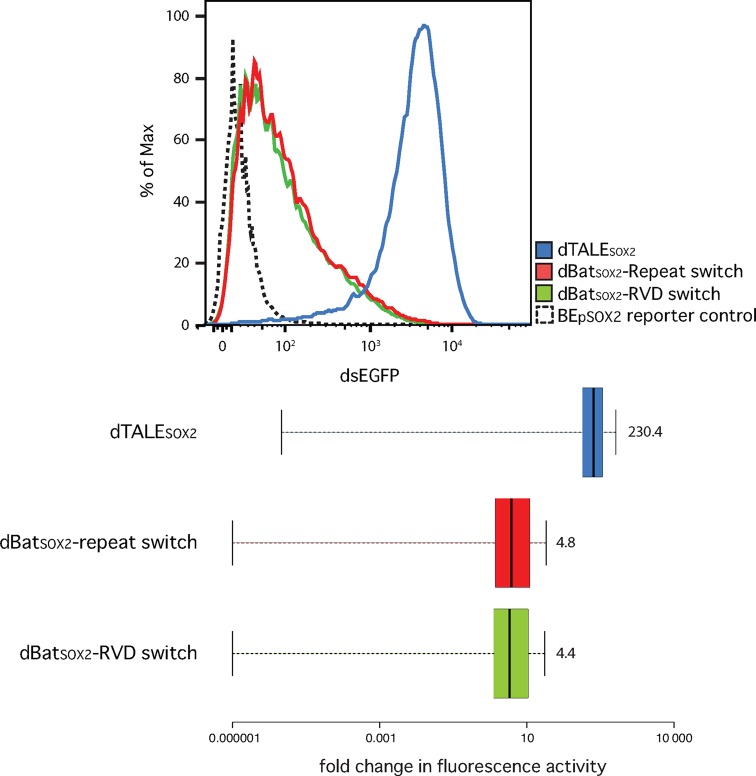
Functional analysis of designer (d)Bat constructs targeting the human SOX2 promoter. dBats were tested using flow cytometry with a transcriptional activation reporter as described (Figure 3). Population curves for dsGFP fluorescence are shown (top) as well as boxplots of fluorescence intensities (bottom) compared to the reporter control (logarithmic scale). Boxplots show fold changes in fluorescence intensity compared to the reporter control with whiskers representing the 2.5% and 97.5% data limits. Median values are written next to each box plot and shown graphically as thick black lines. Two dBats, designed based on the RVD (dBat_SOX2 RVD switch_) or repeat switch (dBat_SOX2 repeat switch_), and an equivalent dTALE were tested.

The repeat domains of dTALE_Bat1mimic_ and dTALE_SOX2_ were created using a previously described method (15). The assembly of dTALE_Bat1mimic_ required modifications to the toolkit. These included a novel level 2 vector, pUC57-CD-DEST, to allow assembly of more than 17 core repeats. This was created using PCR mutagenesis of pUC57 to insert the BsaI sites using primers listed in Supplementary Table S2. Repeats 4_NT, 5B_NN, 4_ND, 7C_NT, 1C_NR, 3_ND, 7D_NS and D ½ N* were created via PCR mutagenesis on described repeat modules (15) or amplification from the repeats of *avrbs3* using the primers listed in Supplementary Table S2.

dTALE_UPT AvrBs3 3x Bat1 rep2/6/8 /17_ were created as previously described (10) with trimers synthesized by GenScript with the sequences listed in Supplementary Figure S14, while dTALE_UPT AvrBs3 3x NI/NN/NG_ were created with the aforementioned *TALE* assembly toolkit (15). Repeat domains were assembled into pENTR-D-TALE Δrep *Bpi*I-AC (15) and then *dTALEs* transferred into T-DNA binary vector pGWB641 (16) via LR recombination (Life Technologies).

### Protein purification

Genes encoding the three N-terminally His tagged Bat proteins (Supplementary Figures S4 and S5) were expressed in *E. coli* Rosetta (DE31) pLaqI (Novagen) as previously described (17). In short, cells were induced at 30°C with IPTG for 3 h. After purification from cell lysate via TALON resin (Clontech), proteins were dialysed against storage buffer (480 mM KCl, 1.6 mM EDTA, 1 mM DTT, 12 mM Tris-Cl, pH 7.5; Slide-A-Lyzer, Thermo Scientific) and concentrated (Amicon Ultra, Millipore).

### Electrophoretic mobility shift assay

Equal amounts of 100 μM 5’ Cy5 labelled forward strand and unlabelled reverse strand oligonucleotides (Metabion) were mixed 1:4 with annealing buffer (TALE storage buffer without DTT or Sodium Azide). After heating to 100°C for 10 min the mixture was allowed to cool to room temperature, then diluted 1/20 in annealing buffer. 2 µl of 1 μM Bat protein was mixed with 16 μl electrophoretic mobility shift assay (EMSA) buffer (15 mM Tris-Cl, 75 mM KCl, 2.5 mM DTT, 0.063% NP-40, 62.5 ng/μl dI.dC, 0.125 mg/ml BSA, 6.25% glycerol, 6.25 mM MgCl, 0.125 mM EDTA) and incubated 5 min at room temperature. 2 µl of target DNA were added followed by a further 30 min incubation. Total binding reactions were run on a 6% native polyacrylamide TBE-gel for 1 h at 100V, 4°C. Cy5 labelled DNA was visualized with the FMBIO III Multi View (Hitachi).

### Microscale thermophoresis

Binding affinity was measured using the Monolith NT.115 from Nanotemper Technologies. Bat1 was labelled with the protein labelling kit RED (Nanotemper) according to the manufacturer's instructions. Differing concentrations of unlabelled Bat1 target DNA (prepared as above) were incubated with 100 nM Bat1 protein in microscale thermophoresis (MST) buffer (Tris 20 mM [pH 7.4], NaCl 150 mM, 10 mM MgCl2 and 0.05% Tween). Samples were loaded into NT.115 Hydrophilic Capillaries. Measurements were performed at room temperature, using 40% LED and 20% IR-laser power. Data analysis and Kd calculations were performed using Nanotemper Analysis software, v.1.4.17 and Origin 9.1.

### Assembly of target plasmids *in vivo* and *in vitro* reporters

For the analysis of reporter activation in human cells target sites were assembled into a BsaI-digested pUC57 derivative with BsaI sites (*TAGA-GGAT*) preceding a minimal CMV promoter followed by a *dsEGFP* reporter gene (18; Supplementary Figure S6). Target sites were introduced as annealed primers (Metabion, annealing as for EMSAs), with matching four base pair overlaps, and were ligated into the BsaI cleaved vectors.

**Figure 8.**
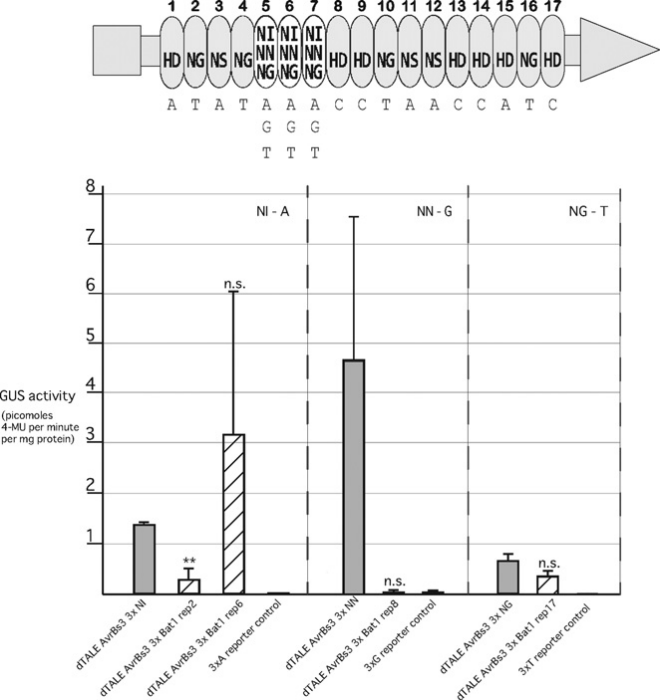
Functional analysis of Bat1 repeats within the context of a TALE repeat array. Trimers of identical Bat1 repeats or TALE repeats with the same RVDs as the Bat1 repeats were embedded into the repeat domain of the 17-repeat TALE AvrBs3 that targets the pepper *Bs3* promoter (*Bs3p*). Repeats 5–7 (3xRVD NI in AvrBs3) where replaced either by TALE repeat trimers with the RVDs NN or NG or by trimers of Bat1 repeats 2, 6, 8 and 17. This is shown in cartoon form with dTALE regions shown in light grey with the trimer of Bat1 repeats or dTALE repeats shown as white ovals. The grey rectangle and triangle indicate the native N- and C-terminal regions of AvrBs3, respectively. RVDs are given in each case and the matching bases in the target box underneath. The resulting chimeras (striped bars) were tested for their ability to activate a *Bs3p* derivative bearing the matching binding site upstream of a *uid*A (GUS) reporter gene and compared to non-chimeric dTALEs (filled bars) with the same RVDs. Dashed lines separate groups of constructs all with the same RVDs and tested against the same reporter. Barred lines indicate standard deviation. Two-tailed t-tests were used to compare chimeric and non-chimeric dTALEs for each reporter. A double asterisk indicates a *P*-value of below 0.02 and n.s. indicates a *P*-value of above 0.05.

To create the target for the *in vitro* cleavage assay, BE_Bat1_ was introduced into the transcriptionally silent *Capsicum annuum Bs3* promoter, previously cloned into pUC57, via mutagenesis PCR (see Supplementary Table S2 for primers and Supplementary Figure S6 for target sequences). The *Bs3* promoter derivatives used in Figure 8 were delivered in modified binary vector pGWB3* upstream of a *uidA* (GUS) reporter gene as previously described (10).

### Transfection of HEK293T cells

HEK293T cells were grown in Dulbecco's modified Eagle's medium—high glucose (Sigma-Aldrich) supplemented with 10% FBS (Sigma-Aldrich), penicillin (100 U/ml) and streptomycin (100 μg/ml) in a 10% CO_2_ atmosphere. 5 × 10^5^ cells were transiently transfected using Fugene (Promega) according to the manufacturer's instructions. Cells were transfected with 3 μg of Bat/TALE expression vector and 300 ng of the *dsEGFP* reporter plasmid.

### Immunohistochemistry and microscopy

For microscopic analysis HEK293T cells were mounted on poly-L-lysine coated glass slides. Forty-eight hours after transfection, the cells were fixed with 4% formaldehyde in phosphate buffered saline (PBS) for 10 min. After permeabilisation with 0.5% Triton X-100 for 10 min, the cells were incubated with 3% bovine serum albumin (BSA) in PBS for 30 min. After 1 h incubation with the primary antibody (1/200 diluted mouse monoclonal antibody ANTI-FLAG M2 (Sigma-Aldrich) in PBS supplemented with 0.05% Tween-20 (PBS-T) and 3% BSA), cells were washed three times with PBS-T. Cells were then incubated with 1/600 Alexa Fluor 594 rabbit Anti-Mouse IgG (Invitrogen) in PBS-T with 3% BSA for 1 h. After washing three times with PBS-T, nuclei were counterstained with 4,6-diamidino-2-phenylindole (DAPI) and stored in 90% Glycerol in PBS with 0.25% DABCO. Images were acquired and processed using a Leica TCS SP5 confocal microscope equipped with an HCX PL APO CS 63x 1.2 Water objective. Images were processed using Leica AF and ImageJ (14).

### FACS analysis of transfected HEK293T cells

Flow cytometry measurements of GFP and Alexa Fluor 594 were performed with a Becton-Dickinson FACS-Aria II. HEK293T cells were harvested, pelleted by centrifugation at 500 x g for 5 min at room temperature and gently washed with PBS. Cells were fixed with 4% formaldehyde in PBS for 10 min, pelleted by centrifugation at 500 x g for 5 min and permeabilized with 0.5% Triton X-100 for 10 min. After pelleting, the cells were incubated in 3% BSA for 30 min and then with mouse monoclonal antibody ANTI-FLAG M2 (Sigma-Aldrich, 1/100 dilution in PBS-T with 3% BSA) for 1 h. Subsequently, the cells were pelleted and washed three times with PBS-T and incubated with Alexa Fluor 594 rabbit anti-mouse IgG (Invitrogen, 1/500 dilution with PBS-T with 3% BSA) for 1 h. The cells were then pelleted and washed three times with PBS-T, stored in 500 μl PBS and analysed with FACS. Data were analysed using FlowJo V 10.0.6 (Tree Star). dsEGFP values for cells with above-threshold (Supplementary Figure S13) Alexa Fluor 594 fluorescence were used in Figures 3, 5–7.

### *In vitro* nuclease assay

*bat1-FokI* and *TALE-FokI* genes were expressed *in vitro* using the Sp6 Quick coupled Transcription/Translation system (Promega) as per manufacturer's instructions. Target DNA was PCR amplified from the previously assembled *Bs3p* derivatives using primers listed in Supplementary Table S2 and purified (GeneJET Gel extraction and DNA clean up Microkit, Life Technologies). Two hundred nanogram of PCR product was incubated with 5 μl transcription/translation product for 3 h at 37°C in cleavage buffer (1x restriction enzyme buffer 4, New England Biosciences, 1 ml/ml BSA, 500 nM NaCl). Reactions were terminated by heating to 60°C and DNA was separated (with kit as above). One hundred nanogram of DNA purified from the cleavage reaction was run on a 2% agarose gel. DNA was visualized via ethidium bromide staining under UV light. Size estimation was made in comparison to a standard ladder (GeneRuler 100 bp plus, Fermentas) and band intensities were measured with ImageJ (14).

### GUS assays

*dTALE* or reporter constructs were transformed into *Agrobacterium tumefaciens* (GV3101) via electroporation. Strains were grown overnight in YEB medium containing rifampicin and kanamycin (each 100 μg/ml; for pGWB3* containing strains) or rifampicin and spectinomycin (each 100 μg/ml; for pGWB641 containing strains), collected by centrifugation, resuspended in inoculation medium (10 mM MgCl_2_, 5 mM MES, pH 5.3, 150 μM acetosyringone) and adjusted to an OD_600nm_ of 0.8. For GUS assays equal amounts of *A. tumefaciens* strains containing *35S*-promoter driven *dTALE* genes and reporter constructs containing corresponding binding boxes fused to the reporter gene *uidA* (*GUS*) were mixed prior to inoculation. Leaf tissue was harvested after 48 h and GUS quantification was carried out as described (10).

## RESULTS

### Three TALE-like proteins are encoded in the genome of *B. rhizoxinica* strain HKI-0454

The Bat polypeptides are formed entirely of repetitive sequences with similarity to those of TALEs (Figures 1A, Supplementary Figures S1 and S2), excluding 17–18 amino acids at the very N-terminus (non-repetitive N-terminal domain; NND). This contrasts from all known TALEs and RipTALs, which possess N-terminal and C-terminal non-repetitive domains of between 100 and 300 amino acids each (Supplementary Figure S2) that are crucial to translocation and their *in planta* function as transcriptional activators (3,10). The Bat proteins can be divided into a set of core repeats all >45% identical to each other at the amino acid level and cryptic repeats not reaching this threshold (Figure 1B, Supplementary Figures S1 and S3; alignments generated with Clusal Omega 19,20). Core repeats are so named as they form the central, and largest, section of the studied polypeptides. Bat1, Bat2 and Bat3 have 20, 26 and 6 core repeats, respectively. The core repeats are framed by two N-terminal (−1, 0) and one C-terminal (+1) cryptic repeat in each Bat protein. The sequence identities of the various domains of the Bat proteins to each other are given in Supplementary Table S1.

Consensus core repeats were deduced for each of the three Bat proteins (Figure 1B and Supplementary Figures S3). Bat1, 2 and 3 consensus repeats are 73–94% identical (Figure 1C, Supplementary Table S1). Each of the three Bat core repeat consensus sequences is less than 40% identical to equivalent consensus repeats of AvrBs3 and Brg11 (AvrBs3 from *X. campestris pv. vesicatoria* and Brg11 from *R. solanacearum* GMI1000 are used here as the representative TALE and RipTAL, respectively). The Bat proteins thus form a highly diverged subgroup of the protein class referred to throughout this publication as ‘TALE-likes’ to mean TALEs, RipTALs and Bat proteins. Despite the high sequence diversity of repeats among TALE-like proteins, 10 residues are conserved in almost all TALE-like repeats and form what we term the ‘consensus TALE-like repeat’ (CTR; Figure 1C). The CTR includes residues clustering around the RVD as well as other residues, such as V22 and L29, able to form stabilising intra-molecular bonds in the crystal structure of DNA-bound TALE dHAX3 (Figure 1C; 4). Given their sequence conservation, the CTR residues are likely to make key contributions to the structure and function of the TALE repeat.

### Bat1 and 2 mediate sequence-specific DNA binding with a code matching the TALE code

TALEs and RipTALs mediate sequence-specific DNA recognition with each core repeat recognising one DNA base and specificity determined by RVDs (the TALE code). We tested whether Bat proteins function similarly. In Bat proteins inter-repeat variability is not limited to the RVDs (positions 12 and 13), in fact position 12 varies very little and the diversity peaks between positions 23–30 (Figure 1B and Supplementary Figure S3). However, we continue to refer to positions 12 and 13 in Bat repeats as the RVD for consistency. The base specificities of most RVDs found in the Bat proteins are known from studies on TALEs and RipTALs allowing us to predict target sequences in each case. The single NR repeat (RVDs and their corresponding repeats are referred to with the single letter amino-acid code throughout) of Bat1 and the three repeats of Bat2 lacking both RVD residues were paired to Guanine and Thymine, based on presumed molecular similarities to NK and N* repeats, respectively.

Genes encoding His-tagged versions of the three Bat proteins were synthesized, expressed in *E. coli* (see Supplementary Figures S4 and S5 for sequences), purified and assayed for binding capabilities in EMSAs against their predicted binding elements (BE_Bat1_, BE_Bat2_ and BE_Bat3_) (Figure 2A; sequences in Supplementary Figure S6). Bat1 and 2 both produced clear shifts in combination with their predicted target DNAs only (Figure 2A). Bat3, which has only six core repeats, was unable to produce a clear shift with any of the target DNAs (Figure 2A). Previous tests with TALEs have shown little activity with TALEs possessing fewer than 10 core repeats (1). It thus seems likely that Bat3 is either non-functional as a DNA-binding protein or mediates very weak interactions, not detectable in this assay.

Bat1 and 2, those displaying DNA binding with a clear sequence preference, are more similar to each other than either is to Bat3 (Supplementary Table S1). The Bat1 and 2 consensus core repeats are 94% identical. Considering the close homology of Bat1 and 2, DNA binding properties are likely conserved and only Bat1 was further characterized.

### Bat1 binds its predicted target with an affinity within the upper boundary of TALE–DNA interactions and without base discrimination at the zero position

MST experiments were carried out to measure the binding strength of Bat1 with BE_Bat1_. We found a disassociation constant (Kd) of 132 nM (Figure 2B). Affinities of TALEs with their target DNAs have been measured at 0.3 to >1000 nM (17), depending on the RVD composition. Yet, stronger interactions than that shown in Figure 2B are thought to be necessary for the *in vivo* function of TALEs. For example, the interaction of TALE AvrBs4 with its target site in the promoter of the pepper *Bs4C* resistance gene was previously measured by MST to have a Kd of 18.1 nM while the interaction with the homologous sequence from the non-activated *bs4C* allele had a Kd of 181.5 nM (21). Given that the affinity of Bat1 to BE_Bat1_ is similar to the affinity of AvrBs4 to the non-activated *bs4C* allele, it is too low to suggest a strong interaction when assuming near-identical physiological conditions. This assumption may not be valid as, for example, the concentration of Bat proteins at the native site of action may differ from that of TALEs on delivery by *Xanthomonas* bacteria. Alternatively, BE_Bat1_ may not represent the optimal binding sequence or additional endogenous factors may promote interaction *in vivo*.

BE_Bat1_ was created in accordance with the TALE requirement for a thymine at the zero position (T_0_ preference). However, the RipTALs do not share the T_0_ preference and instead activate only G_0_ targets (10). Therefore, we carried out further MST experiments with the different N_0_, bases to clarify whether the T_0_ preference holds for Bat1 or if another base is preferred. We found that in fact no significant differences were seen in the Kds of the different N_0_ base target DNAs (Figure 2C and Supplementary Figure S7 and Table S3). This accords with the results of Juillerat *et al.* (22) using an *in vivo* reporter system. All further experiments were carried out using T_0_ targets to allow optimal conditions for comparison to TALE controls.

### The fusion of NLSs and AD are sufficient to convert Bat1 into a targeted transcription factor in human cells and *in planta*

Having demonstrated that Bat1 binds its predicted target sequence *in vitro*, we developed a Bat1 derivative to function *in vivo* as a transcriptional activator and tested this with reporter assays. A Bat1 transcriptional activator (acBat1) was created through translational fusion of a viral NLS and a VP64 AD. A 3xFLAG epitope tag between NLS and VP64 domain (Supplementary Figure S5) allowed for antibody-based protein detection using an Alexa Fluor 594-tagged secondary antibody. We measured the ability of acBat1 to activate a dsEGFP-based reporter gene (18) in human cells (HEK293T; Figure 3A). A custom TALE-activator construct was tested in parallel. Termed dTALE_Bat1mimic_, it has the same repeat number and RVD composition as Bat1 and the same fused domains (Figure 3A, Supplementary Figures S5 and S8). Immunostaining showed that the acBat1 and dTALE_Bat1mimic_ both localized to the nucleus, while acBat1-ΔNLS, lacking the NLSs, did not localize to the nucleus. This demonstrates that NLSs must be added to Bat1 in order to target it to the nucleus in human cells (Figure 3B). dsEGFP expression in cells expressing acBat1 showed that it is able to activate the reporter. By contrast, cells expressing a derivative lacking the AD (acBat1-ΔAD) showed only Alexa Fluor 594 fluorescence, but did not show dsEGFP fluorescence indicating that the reporter was not activated (Figure 3B). Fusion of an AD is thus necessary to convert Bat1 into a functional transcriptional activator in human cells.

acBat1 induced the reporter 5-fold, while the dTALE_Bat1mimic_ induced the reporter 20-fold (Figure 3C). This may indicate that dTALE_Bat1mimic_ has a higher affinity for BE_Bat1_ than acBat1 does. Alternatively, the activity of the C-terminally fused VP64 AD may be differentially affected by the architecture of each fusion protein.

To study functionality of acBat1 *in planta*, a corresponding T-DNA construct was delivered via *A. tumefaciens* into *Nicotiana benthamiana* leaves. In this assay, constitutively expressed acBat1 activated a co-delivered *uidA* reporter gene downstream of a promoter bearing BE_Bat1_ (Supplementary Figure S9). In analogy to the results observed in human cells, the dTALE_Bat1mimic_ control was able to activate the reporter in plant cells to 3-fold higher levels than acBat1. In sum, we were able to show that acBat1 can transcriptionally activate a promoter with its target sequence in both human and plant cells.

### Fusion of a FokI domain to the C-terminus of Bat1 creates a sequence-specific DNA nuclease

The most common approach for the creation of TALE-nucleases (TALENs) is a C-terminal translational fusion to a FokI endonuclease domain. Since the FokI endonuclease is active only as a dimer, interaction of two FokI domains is achieved by placing neighbouring TALEN target sites on opposite strands in reverse orientation promoting interaction of the FokI monomers after DNA binding. The FokI dimer catalyses formation of a double-strand break in the DNA spacer region between the two TALEN target sites. We created an analogous architecture using Bat1 to confer DNA binding specificity and compared its activity in an *in vitro* cleavage assay against the corresponding TALEN (dTALE_Bat1mimic_-FokI; sequences given in Supplementary Figure S5). As target DNA we used a PCR product bearing two copies of BE_Bat1_ in reverse orientation on opposite strands. We generated derivatives differing only in the length of the DNA spacer separating the targets (Supplementary Figure S6) in order to determine the spacing between the two target sites that would result in the highest activity of the Bat-FokI fusion proteins. As a negative control, we tested a template with a control sequence instead of the Bat1 target sites.

Bat1-FokI and dTALE_Bat1mimic_-FokI were expressed *in vitro* and equal volumes of reaction product were incubated with the target DNA. After 3 h at 37°C the DNA was size fractionated on a 2% agarose gel (Figure 4). Both Bat1 and TALE nucleases were able to cleave the target constructs. By contrast, the controls lacking target sites were not cleaved, indicating that Bat1-FokI, like the TALEN, is target specific in its DNA cleavage. The highest efficacy shown by Bat1-FokI was 35% cleavage (11 bp spacer) while dTALE_Bat1mimic_-FokI had a maximum efficacy of 86% cleavage (19 bp spacer; Figure 4). That dTALE_Bat1mimic_-FokI showed greater flexibility with respect to spacer length may relate to the previously optimized architecture employed (18). TALEN architecture is known to play a decisive role in spacer preference (23). Similarly, alternative Bat1 truncations or peptide linkers might allow for the creation of Bat1 nucleases with greater flexibility in spacer length.

### The paradigm underlying the modification of core and cryptic TALE repeats cannot be applied to Bat1

In both natural (3) and custom TALEs, the number of core repeats is flexible, within a certain range. The number and position of cryptic N- and C-terminal repeats are typically inflexible, though alternative repeat −1 modules have recently been described (24,25). We tested acBat1 deletion derivatives to test if this paradigm applies to Bat1.

First, we tested variants of acBat1 lacking 2 (Δ18–20), 4 (Δ16–20), 6 (Δ14–20) or 8 (Δ12–20) core repeats (Figure 5A and Supplementary Figure S10). The later half of repeat 20 and repeat +1 were retained in each case. These truncations were tested against the BE_Bat1_ reporter and produced varied levels of reporter activation (Figure 5A). acBat1-Δ18–20 was able to activate the reporter more than 2-fold, corresponding to 40% activity of wild-type acBat1. The other truncation derivatives were unable to activate the reporter to levels above background. If we assume that each repeat contributes a certain amount of affinity to the Bat1–BE_Bat1_ interaction then fewer than 17 repeats may simply be insufficient for an interaction strong enough to lead to reporter activation. This is in accordance with results from TALE repeat arrays showing that a certain number of core repeats are necessary for downstream reporter gene activation (1). Alternatively, the novel interface formed within the last repeat in each truncation derivative may create unfavourable intramolecular interactions, reducing protein activity. This explanation would not apply to TALEs where repeats are near identical and repeat order does not change the interface between repeats. Given the numerous non-RVD polymorphisms between Bat1 repeats, deletion or insertion of core repeats will always create novel repeat interfaces and should be experimentally validated before use in downstream applications.

We next tested acBat1 derivatives where the 82 residues N-terminal of core repeat 1 (acBat1ΔNTD; lacking repeats 0 and −1), or the 30 residues C-terminal of core repeat 20 (acBat1ΔCTD, lacking repeat +1) were deleted (Figure 5B and Supplementary Figure S10). Whilst acBat1ΔNTD showed a modest reduction in activity (56% of acBat1), acBat1ΔCTD was barely able to activate the reporter above background (Figure 5B). This does not match expectations based on TALEs where only the cryptic N- but not the cryptic C-terminal repeats are essential for DNA binding (26). By contrast, our results suggest that the cryptic C-terminal Bat1 repeat +1, in contrast to the corresponding cryptic TALE repeat +1, makes an unexpectedly strong contribution to activity and thus should be retained for the creation of active Bat1-based transcriptional activators.

### Despite high inter-repeat diversity designer Bat1 proteins (dBats) with wild-type levels of activity can be assembled

The non-RVD residues of Bat1 repeats are highly polymorphic. This provides a means to study the functional relevance of non-RVD polymorphism in the native Bat1 as well as being relevant for the creation of Bat1 derivatives with novel specificity (dBats). We hypothesize that non-RVD polymorphisms may have two functionally relevant, non-mutually-exclusive, effects. (i) The formation of unique but functionally equivalent repeat interfaces that stabilize the superhelical structure formed by tandem-arranged repeats (4,5) (superstructural hypothesis). (ii) The creation of unique scaffolds optimized for the native RVD residues in each case (RVD scaffold hypothesis).

We used two different dBat design methods to test our hypotheses. These are the repeat switch and the RVD switch. Sequences of the dBats created can be found in Supplementary Figure S11. In the repeat switch whole repeats, including their native RVDs, were exchanged. This creates new interfaces between repeats but leaves RVDs in their native repeat context. If the superstructural hypothesis is correct then the repeat switch is likely to modify evolved repeat interfaces possibly yielding less active DNA-binding proteins. In the RVD switch it is only the RVDs that are changed while all non-RVDs remain unchanged. This design will not change repeat interfaces but will place RVDs in non-native repeat scaffolds. If the RVD scaffold hypothesis is correct then the RVD switch will reduce activity due to RVDs being sub-optimally oriented in relation to the paired DNA bases.

RVD composition and target sequence are key parameters determining affinity of TALE–DNA interactions and these were kept constant in our dBat tests as far as possible. For the repeat switch tests, we exchanged repeats with RVDs paired to the same base in BE_Bat1_ allowing the wild-type target construct to be used in each case. For the RVD switch constructs, where possible we exchanged RVDs with the same target base (dBat RVD switch 3 and 4) and tested these constructs against BE_Bat1_. Where this was not possible exchanges were made between repeats in close proximity to one another to reduce any influence from an N- to C-terminal polarity effect as known for TALEs (17,27–29). These were then tested against BE_Bat1_ derivatives with the appropriate minor modifications in base composition. Thus any differences we see in activity are likely to be linked to effects arising from manipulation of repeats and not to differences in RVD composition or target sequence.

We found that despite the minor modifications in each case the different dBat constructs mediated strikingly varied levels of reporter activation. Of the four RVD switch constructs two were superior in activation level compared to acBat1 (2.9x and 1.4x relative to acBat1; Figure 6A). The other two dBat derivatives were slightly reduced in their activity as compared to acBat1 (0.56x and 0.72x relative to acBat1; Figure 6A). Overall, the impact on activity of the RVD switch constructs showed no single trend with some superior and some inferior to the wild type. Of the four repeat switch constructs none reached the activation level of acBat1 (Figure 6B). Notably, dBat repeat switch 3, in which core repeats 11 and 12 were exchanged, was unable to induce the reporter above background levels. Thus the repeat switch constructs all showed reduced activity compared to the wild type, and some dramatically so.

These data support that inter-repeat interfaces are unique and optimized (superstructural hypothesis) though whether the same is true for RVD scaffolds is not clear. That the RVD switch constructs performed differentially suggests that RVD scaffold can have a functional impact. However, the natural scaffold does not seem to be the optimal one in every case.

### Custom dBats can be created to target a novel, user-defined sequence

We next tested whether the Bat1 repeat array could be fully customized to target a sequence of interest. Based on the two alternative strategies described above, dBat*_SOX2_*-RVD switch and dBat*_SOX2_*-repeat switch were created to activate a dsEGFP reporter driven from a minimal CMV promoter containing a binding element taken from the human *SOX2* promoter (Supplementary Figures S6, S8 and S11 for protein and reporter sequences). The SOX2 protein prevents determination in human neural stem cells and has previously been a target for dTALE studies (30). Both dBat repeat arrays were limited to 18 repeats instead of the wild-type 20 to bring them in line with the length of custom TALE repeat arrays commonly produced with our toolkit (15). The same NLS and VP64 fusions were used as for the assays displayed in Figure 3A. Both dBats were able to activate the reporter to similar levels (Figure 7) suggesting that both the RVD and repeat switch strategies can yield successful constructs. dBat_SOX2_-repeat switch mediated 4.8x reporter activation and thus was slightly more active than the dBat_SOX2_-RVD switch (4.4x reporter activation). However, as seen previously (Figure 6), results can be surprisingly varied even between very similar dBat constructs and any potential design should be tested first in a reporter system before further application. Cross-reactivity assays testing the *SOX2* dBats on the BE_Bat1_ reporter showed that they were unable to activate the non-target reporter above background (Supplementary Figure S12) indicating that target specificity is maintained in the dBats. Further work on the creation of Bat1-based arrays and fusion proteins may improve activity levels. In conclusion, we were successfully able to reprogram the Bat1 protein for the creation of transcriptional activators with novel specificity.

### TALE-Bat1 chimeras show varied activity but may be a means to harness the sequence diversity of Bat1 repeats

While the activation achieved with the SOX2 dBats was encouraging a custom TALE-activator for the *SOX2* promoter (dTALE_SOX2_) activated the reporter more than 200-fold (Figure 7). It may be possible to improve the activation levels achieved with dBats through further work on construct design and indeed Bat1 nuclease activities matching the corresponding activities of corresponding TALE nucleases were previously reported (22). However, another possibility is to create chimeric proteins to combine desirable features of both the Bat and TALE repeat scaffold.

We tested the principle of creating TALE-Bat chimeric repeat domains utilising a simple assay approach previously used in our lab to test chimeric TALE-RipTAL repeat arrays (10). Three identical copies of different Bat repeats were used to replace three repeats in a dTALE targeting the pepper *Bs3* promoter (*Bs3p*). These were then tested *in planta* against a reporter construct bearing a *Bs3p* fragment upstream of a *uidA* (GUS) gene. Three different reporters were used with triple A, G or T at the position that should be bound by the inserted Bat repeats in order to test repeats with different RVDs. In each case comparison was made to a dTALE assembled using only TALE repeats with the same RVD as the Bat repeats. As with earlier dBat tests we found strikingly different results for different constructs (Figure 8).

dTALE_AvrBs3_3xBat1_rep2_, a dTALE bearing three copies of Bat1 repeat 2 (RVD NI) at the test positions, gave a significantly weaker induction of the reporter compared to the control with TALE repeats only (dTALE _AvrBs3_3xNI_). dTALE_AvrBs3_3xBat1_rep8_ (RVD NN) was barely able to elicit any detectable activation, unlike its TALE repeat equivalent (dTALE_AvrBs3_3xNN_). In contrast, dTALE_AvrBs3_3x_Bat1 rep6_ (RVD NI) and dTALE_AvrBs3_3xBat1_rep17_ (RVD NG) activated their reporters to a level not significantly different from the TALE repeat control constructs. It is not possible to clarify whether differences in functionality arise from performance differences between Bat or TALE repeats in their native confirmations or if the differences arise due to the formation of novel and likely unfavourable inter-repeat interactions in these chimeric constructs (see superstructural hypothesis above). The functionality of any potential chimeric binding domain is likely to depend on both the particular repeats utilized and their arrangement within the repeat domain. However, we have demonstrated that such chimeric repeat domains containing some Bat1 repeats can be functional to the same level as TALE repeat equivalents paving the way for further development and applied uses.

## DISCUSSION

The Bat proteins, together with the TALEs and RipTALs, form the TALE-like protein class. Like the other TALE-likes, Bat proteins mediate sequence-specific DNA binding with specificity predicted from the established TALE code. This functional similarity likely correlates to a structural similarity since DNA recognition proceeding via the TALE code relies on a particular structure that places position 13 of each repeat in close proximity to a single DNA base (4,5). Indeed modelling the structure of Bat1 based on the known structure of TALE Pthxo1 binding to its target DNA (5) suggests that the whole Bat1 polypeptide would form a sequence aligning closely to the TALE core repeat domain (Supplementary Figure S15).

Comparison of the core repeats of distinct TALE-likes enabled us to define a set of conserved residues, the CTR, as a unifying feature of the TALE-like proteins (Figure 1C). The CTR could be a useful tool to scan databases for further TALE-likes. In addition, the conservation of CTR residues suggests that they have an important functional relevance. Intriguingly, the CTR residues do not include some repeat residues such as K16, which have been shown to provide a large contribution to non-base-specific DNA binding, or H33, suggested as key to stabilisation of the TALE repeat (31). Conversely, some CTR residues such as L29 cannot currently be linked to a certain key function. Thus, investigation of the TALE-likes provides an interesting window into the opportunities for and constraints on sequence diversification whilst maintaining protein function.

We have demonstrated that the Bat1 protein itself can be taken as a targeting module for transcriptional activation (Figure 3) and nuclease function (Figure 4). The repeat array can also be reprogrammed to target a sequence of interest (Figure 7). Unlike the reprogramming of TALEs, alternative design strategies must be considered to generate Bat1 repeat arrays with desired base specificity and we have successfully employed two conceptually distinct design approaches (Figure 6). However, Bat1 and derivative fusion proteins were outperformed by equivalent TALE fusions (Figures 3, 4 and 7). This may relate to the relatively low affinity of Bat1 for BE_Bat1_ (Figure 2B) compared to known affinities of TALEs for their natural target boxes. However, the TALE platform has been optimized over several years. The creation of high activity TALE-nucleases, in particular, has been a focus of many labs. Thus, with further work to improve activity, the Bat platform may prove a more compact alternative to TALEs for targeted DNA binding without any zero base preference to be taken into account (Figure 2C). Alternatively, Bat repeats could be assembled along with TALE repeats to create chimeric DNA-binding proteins with novel properties. At the very least the inclusion of some Bat repeats into TALE repeat arrays would lower sequence identity between repeats, useful for some cloning strategies, and possibly alleviating the previously reported problem of recombinatorial repeat loss (32). That Bat1 repeats can be integrated into a dTALE whilst retaining functionality is shown in Figure 8, but since no two Bat1 repeats are identical, so too must each Bat1-TALE chimera be treated as novel and requiring experimental validation before further use.

Functionally relevant differences between TALEs and Bat proteins were discovered upon attempting to modify the repeat domain. Bat1 showed surprisingly little tolerance to reductions in repeat number below 18 repeats (Figure 5A). These results seem to be in agreement with analysis of TALE proteins where a minimum number of repeats was needed to achieve *in vivo* function (1). The conclusion that has been drawn from such analysis is that each TALE repeat contributes something towards affinity and that a certain number of repeats are required to achieve the affinity necessary for *in vivo* function. However, the situation for Bat proteins is more complex. Due to the numerous non-RVD polymorphisms between each repeat (Figure 1B), a novel interface is formed when truncations are made within the repeat domain and these could have functionally deleterious consequences. Indeed the results of rearrangements within the repeat domain (Figure 6B) suggest that this is so.

A further difference between Bat1 and TALEs is the relative impact of truncations of the N- and C-terminal cryptic repeats. The N-terminal cryptic repeats of TALEs make a decisive contribution to DNA affinity such that their removal fully ablates DNA binding (26). By contrast, the limited evidence available suggests that the C-terminal cryptic repeats of TALEs contribute little to affinity and specificity. This includes the independently observed (17,27–29) N- to C-terminal reduction along the binding domain of contribution to base specificity. In addition, TALE fusion proteins with truncations in C-terminal cryptic repeat +2 (Supplementary Figure S2) are active (18) suggesting that any affinity contribution is not decisive. Thus in TALEs the N-terminal cryptic repeats seem to contribute more to DNA binding than the C-terminal cryptic repeats. This contrasts to our findings based on truncations of the N- and C-terminal cryptic repeats of acBat1. We found that the N-terminal truncation had a modest impact on reporter activation and did not contribute to specificity (Figures 2C, 5B and Supplementary Figure S7 and Table S3), whilst the truncation of the single C-terminal cryptic repeats almost entirely ablated activity (Figure 5B). This repeat may be important for DNA binding and the high proportion of positively charged residues (8/30; Supplementary Figure S1) is in agreement with a possible contribution to interaction with the negatively charged DNA phosphate backbone. Sequence comparison of the cryptic repeats of Bats and AvrBs3 (see Supplementary Figures S1 and S2) showed that the 0 repeats share a few residues (L1, L7 and K8) not found in the CTR (Figure 1C) but no such unique conserved residues can be found among the -1 or +1 repeats. Together with the results shown in Figure 5B it appears that, at both the sequence and functional level, at least the cryptic repeats -1 and +1 of Bats and TALEs are likely to be non-homologous.

Through the exploration of dBat assembly strategies, we gained insights into the functional significance of Bat1 non-RVD polymorphisms. These polymorphisms provided a molecular handle to question different models. The results of these experiments are possibly specific to Bat proteins but most likely are relevant to the non-RVD polymorphisms of other TALE-like proteins. The RVD switch constructs (Figure 6A) tested the importance of the RVD scaffold formed by all the non-RVD residues of a repeat, while the repeat switch constructs tested the importance of inter-repeat interactions (Figure 6B). We found that all repeat switch constructs were less active than the wild type (Figure 6B). This supports the hypothesis that the non-RVD polymorphisms of adjacent Bat1 repeats lead to the formation of unique but functionally equivalent interfaces between repeats. Our model for the structure of Bat1 bound to DNA suggests that unique bonds are indeed formed between varied residues of Bat1 repeats (Supplementary Table S4). Perturbation of these possibly co-evolved residues would likely impair protein function. The performances of the RVD-switch constructs (Figure 6A) were mixed, with some activating the reporter better than the wild-type acBat1. This speaks against the idea that each repeat scaffold has co-evolved with its RVD for optimal activity. The data do, however, support previous findings from RipTALs (10) and TALEs (33) that certain non-RVD polymorphisms can have profound effects on repeat activity. These effects can be negative or positive and must be investigated individually. The quantity of non-RVD polymorphisms in Bat1 repeats compared to TALEs (3) or RipTALs (10) thus complicates the creation of designer DNA binding domains but also represents an as yet unexploited pool of potentially beneficial repeat variants.

Comparing the diversity of Bat and TALE repeats also raises evolutionary questions. The consensus core repeats or TALEs and Bats are less than 40% conserved (Figure 1C) at the sequence level, but at the functional level Bat and TALE repeats are apparently very similar. This shows that the sequence composition of TALE-like repeats is not heavily constrained by functional requirements. If most polymorphisms are functionally equivalent we would expect that, over time, inter-repeat polymorphisms would accumulate. The high levels of inter-repeat polymorphism in the Bat proteins (Figure 1B and Supplementary Figure S3) are consistent with this assumption. What is surprising is the relative sequence uniformity of TALE repeats. This suggests that TALE repeats are under the influence of a selective pressure to maintain sequence conservation, not felt by Bat proteins. However, while the non-RVDs of each TALE repeat are highly uniform the RVD composition and repeat number are highly diverse (3). These observations may be mutually explanatory. It is known that repeat regions of *TALE* genes can evolve via intra- and inter-molecular recombination (34,35). It may be, therefore, that the sequence conservation between individual *TALE* repeats promotes this recombination and subsequent diversification of repeat number and RVD composition. This property may be positively selected for in *TALE* genes. These assumptions and hypotheses require further testing, but comparison to non-*Xanthomonas* TALE-likes will likely prove a helpful one. Indeed the RipTALs, which show intermediate sequence diversity and limited structural diversity (10), provide an interesting third group for comparison.

We have shown that the Bat proteins are a highly divergent subgroup within a class referred to as the TALE-likes, which they help to define. Moreover, Bat specificity can be programmed with a code matching to known TALE and RipTAL repeat specificity (Figure 2A). Bat proteins thus represent an alternative platform for programmable sequence-specific DNA targeting. In addition, the highly diverse Bat repeats may prove a valuable reservoir for novel residue combinations with beneficial properties. More than this they provide an out-group for comparative analysis into function and evolution of RipTALs and TALEs. Further research into the Bat proteins is thus likely to reap rewards for both fundamental and applied research.

## SUPPLEMENTARY DATA

Supplementary Data are available at NAR Online.

SUPPLEMENTARY DATA
