# Supplementary material for: Programmable DNA-binding proteins from Burkholderia provide a fresh perspective on the TALE-like repeat domain
Source: Nucleic Acids Res. 2014 May 3;42(11):7436–49. doi: 10.1093/nar/gku329 (PMC4066763; doi:10.1093/nar/gku329)
Supplement: SUPPLEMENTARY DATA [file supp_42_11_7436__index.html]

Programmable DNA-binding proteins from Burkholderia provide a fresh perspective on the TALE-like repeat domain — Programmable DNA-binding proteins from Burkholderia provide a fresh perspective on the TALE-like repeat domain — Programmable DNA-binding proteins from Burkholderia provide a fresh perspective on the TALE-like repeat domain — SUPPLEMENTARY DATA 

# Programmable DNA-binding proteins from *Burkholderia* provide a fresh perspective on the TALE-like repeat domain

## SUPPLEMENTARY DATA

**Files in this Data Supplement:**

- SUPPLEMENTARY DATA
