# Supplementary material for: Programmable DNA-binding proteins from Burkholderia provide a fresh perspective on the TALE-like repeat domain
Source: Nucleic Acids Res. 2014 May 3;42(11):7436–49. doi: 10.1093/nar/gku329 (PMC4066763; doi:10.1093/nar/gku329)
Supplement: SUPPLEMENTARY DATA [file supp_gku329_nar-03614-h-2013-File010.doc]

**de Lange *et al.* – Supplement**

**Supplementary Figures**

S1 – Annotated amino acid sequences of Bat1, Bat2 and Bat3

S2 – Annotated amino acid sequences of AvrBs3 and Brg11

S3 – Amino acid alignments of the Bat2 and Bat3 core repeats.

S4 – Nucleotide sequences of synthesised *Bat1*, *Bat2* and *Bat3* genes.

S5 – Sequences of translational fusions for protein purification, transcriptional activation reporters and nuclease assay.

S6 – Target and reporter sequences used in this study.

S7 – MST results for Bat1 measured against BE_Bat1 T-0, A-0, C-0 and G-0_.

S8 – Amino acid sequence of dTALEs used in this study.

S9 – *in planta* transcriptional activation mediated by acBat1.

S10 – Amino acid sequences of acBat1 derivatives (dBats) tested in Figures 5 and 6.

S11 – Nucleotide and amino acid sequences of dBat_SOX2_-RVD switch and -repeat switch.

S12 – Specificity test with the BE*_pSOX2_* targeted dBats.

S13 – Pseudocolour density blots of fluorescence and extended boxplots including outliers for experiments shown in Figures 3, 5-7.

S14 – Sequences of Bat1 repeat trimers used in Figure 8.

S15 – Structural predictions for Bat1 based on the structure of PthXo1 bound to DNA.

**Supplementary Tables**

S1 – Sequence identities of the Bat proteins sorted by domain.

S2 – Sequences of primers used in this study.

S3 – Hydrogen bonds predicted to be formed between repeats of Bat1 based on the structure shown in Figure S15.

**Supplementary Figure 1:** Annotated amino acid sequences of Bat1, Bat2 and Bat3

Annotated sequences of the three predicted Bat proteins. Each is formed of a short Non-repetitive N-terminal Domain (NND) followed by an array of cryptic (-1, 0, +1) and core repeats (1, 2, 3…). Consecutive repeats are numbered (left side). The RVDs (residues at repeat positions 12 and 13) are marked as boldface black letters on grey background. Blue lettering is used for the positively charged residues within repeat +1.

>Bat1 (from *Burkholderia rhizoxinica* strain HKI-0454 plasmid pBRH01, GenBank NC_014718.1, RBRH_01844; Uniprot E5AV36)

NND MSTAFVDQDKQMANRLN

-1 LSPLERSKIEKQYGGATTLAFISNKQNELAQI

0 LSRADILKIASYDCAAHALQAVLDCGPMLGKRG

1 FSQSDIVKIAG**NI**GGAQALQAVLDLESMLGKRG

2 FSRDDIAKMAG**NI**GGAQTLQAVLDLESAFRERG

3 FSQADIVKIAG**NN**GGAQALYSVLDVEPTLGKRG

4 FSRADIVKIAG**NT**GGAQALHTVLDLEPALGKRG

5 FSRIDIVKIAA**NN**GGAQALHAVLDLGPTLRECG

6 FSQATIAKIAG**NI**GGAQALQMVLDLGPALGKRG

7 FSQATIAKIAG**NI**GGAQALQTVLDLEPALCERG

8 FSQATIAKMAG**NN**GGAQALQTVLDLEPALRKRD

9 FRQADIIKIAG**ND**GGAQALQAVIEHGPTLRQHG

10 FNLADIVKMAG**NI**GGAQALQAVLDLKPVLDEHG

11 FSQPDIVKMAG**NI**GGAQALQAVLSLGPALRERG

12 FSQPDIVKIAG**NT**GGAQALQAVLDLELTLVEHG

13 FSQPDIVRITG**NR**GGAQALQAVLALELTLRERG

14 FSQPDIVKIAG**NS**GGAQALQAVLDLELTFRERG

15 FSQADIVKIAG**ND**GGTQALHAVLDLERMLGERG

16 FSRADIVNVAG**NN**GGAQALKAVLEHEATLNERG

17 FSRADIVKIAG**NG**GGAQALKAVLEHEATLDERG

18 FSRADIVRIAG**NG**GGAQALKAVLEHGPTLNERG

19 FNLTDIVEMAA**NS**GGAQALKAVLEHGPTLRQRG

20 LSLIDIVEIAS**N**-GGAQALKAVLKYGPVLMQAG

+1 RSNEEIVHVAARRGGAGRIRKMVAP---LLERQ

>Bat2 (from *Burkholderia rhizoxinica* strain HKI-0454 plasmid pBRH02, GenBank NC_014723.1, RBRH_01776; Uniprot E5AW45)

NND MPATSMHQEDKQSANGLN

-1 LSPLERIKIEKHYGGGATLAFISNQHDELAQV

0 LSRADILKIASYDCAAQALQAVLDCGPMLGKRG

1 FSRADIVRIAG**NG**GGAQALYSVLDVEPTLGKRG

2 FSQVDVVKIAG--GGAQALHTVLEIGPTLGERG

3 FSRGDIVTIAG**NN**GGAQALQAVLELEPTLRERG

4 FNQADIVKIAG**NG**GGAQALQAVLDVEPALGKRG

5 FSRVDIAKIAG--GGAQALQAVLGLEPTLRKRG

6 FHPTDIIKIAG**NN**GGAQALQAVLDLELMLRERG

7 FSQADIVKMAS**NI**GGAQALQAVLNLEPALCERG

8 FSQPDIVKMAG**NS**GGAQALQAVLDLELAFRERG

9 FSQADIVKMAS**NI**GGAQALQAVLELEPALHERG

10 FSQANIVKMAG**NS**GGAQALQAVLDLELVFRERG

11 VRQADIVKIVG**NN**GGAQALQAVFELEPTLRERG

12 FNQATIVKIAA**NG**GGAQALYSVLDVEPTLDKRG

13 FSRVDIVKIAG--GGAQALHTAFELEPTLRKRG

14 FNPTDIVKIAG**NK**GGAQALQAVLELEPALRERG

15 FNQATIVKMAG**NA**GGAQALYSVLDVEPALRERG

16 FSQPEIVKIAG**NI**GGAQALHTVLELEPTLHKRG

17 FNPTDIVKIAG**NS**GGAQALQAVLELEPAFRERG

18 FGQPDIVKMAS**NI**GGAQALQAVLELEPALRERG

19 FSQPDIVEMAG**NI**GGAQALQAVLELEPAFRERG

20 FSQSDIVKIAG**NI**GGAQALQAVLELEPTLRESD

21 FRQADIVNIAG**ND**GSTQALKAVIEHGPRLRQRG

22 FNRASIVKIAG**NS**GGAQALQAVLKHGPTLDERG

23 FNLTNIVKIAG**NG**GGAQALKAVIEHGPTLQQRG

24 FNLTDIVEMAG**KG**GGAQALKAVLEHGPTLRQRG

25 FNLIDIVEMAS**NT**GGAQALKTVLEHGPTLRQRD

26 LSLIDIVEIAS**N**-GGAQALKAVLKYGPVLMQAG

+1 RSNEEIVHVAARRGGAGRIRKMVAL---LLERQ

>Bat3 (from *Burkholderia rhizoxinica* strain HKI-0454 plasmid pBRH02 GenBank NC_014723.1, RBRH_01777; Uniprot E5AW45)

NND MPVTSVYQKDKPFGARLN

-1 LSPFECLKIEKHSGGADALEFISNKYDALTQV

0 LSRADILKIACHDCAAHALQAVLDYEQVFRQRG

1 FARADIIKITG**NG**GGAQALKAVVVHGPTLNECG

2 FSQADIVRIAD**NI**GGAQALKAVLEHGPTLNERD

3 YSGADIVKIAG**NG**GGARALKAVVMHGPTLCESG

4 YSGADIVKIAS**NG**GGAQALEAVAMHGSTLCERG

5 YCRTDIAKIAG**NG**GGAQALKAIVMHGPTLCERG

6 YSRTDIVKIAD**NN**GGAQALKAVFEHGPALTQAG

+1 RSNEDIVNMAARTGAAGQIRKMAAQ---LSGRQ

**Supplementary Figure 2:** Annotated amino acid sequences of AvrBs3 and Brg11

AvrBs3 and Brg11 are the first characterised TALE and RipTAL respectively (36, 10). Annotated amino-acid sequences are given for Brg11 and AvrBs3. N-terminal and C‑terminal non-repeat regions and the central repeat array are displayed in separate paragraphs but are part of contiguous polypeptides. Consecutive repeats are numbered (left side). Repeats can be divided into cryptic (-1, 0, +1, +2) and core (1, 2, 3…). The RVDs (residues at repeat positions 12 and 13) are marked as boldface black letters on grey background.

>AvrBs3 (from *Xanthomonas campestris* pv*. vesicatoria* strain 71-21; GenBank CAA34257.1)

MDPIRSRTPSPARELLPGPQPDGVQPTADRGVSPPAGGPLDGLPARRTMSRTRLPSPPAPSPAFSAGSFSDLLRQFDPSLFNTSLFDSLPPFGAHHTEAATGEWDEVQSGLRAADAPPPTMRVAVTAARPPRAKPAPRRRAAQPSDASPAAQVDLRTLGYSQQQQEKIKPKVRSTVAQHHEALVGHGFTHAHIVALSQHPAALGTVAVKYQDMIAALPE

-1 ATHEAIVGVGKQWSGARALEALLTVAGELRGPPLQ

0 LDTGQLLKIAKR-GGVTAVEAVHAWRNALTGAPLN

1 LTPEQVVAIAS**HD**GGKQALETVQRLLPVLCQAHG

2 LTPQQVVAIAS**NG**GGKQALETVQRLLPVLCQAHG

3 LTPQQVVAIAS**NS**GGKQALETVQRLLPVLCQAHG

4 LTPEQVVAIAS**NG**GGKQALETVQRLLPVLCQAHG

5 LTPEQVVAIAS**NI**GGKQALETVQRLLPVLCQAHG

6 LTPEQVVAIAS**NI**GGKQALETVQRLLPVLCQAHG

7 LTPEQVVAIAS**NI**GGKQALETVQRLLPVLCQAHG

8 LTPEQVVAIAS**HD**GGKQALETVQRLLPVLCQAHG

9 LTPEQVVAIAS**HD**GGKQALETVQRLLPVLCQAHG

10 LTPQQVVAIAS**NG**GGKQALETVQRLLPVLCQAHG

11 LTPEQVVAIAS**NS**GGKQALETVQALLPVLCQAHG

12 LTPEQVVAIAS**NS**GGKQALETVQRLLPVLCQAHG

13 LTPEQVVAIAS**HD**GGKQALETVQRLLPVLCQAHG

14 LTPEQVVAIAS**HD**GGKQALETVQRLLPVLCQAHG

15 LTPEQVVAIAS**HD**GGKQALETVQRLLPVLCQAHG

16 LTPQQVVAIAS**NG**GGRPALETVQRLLPVLCQAHG

17 LTPEQVVAIAS**HD**GGKQALETVQRLLPVLCQAHG

+1 LTPQQVVAIASNGGGRPALESIVAQLSRPDPALAA

+2 LTNDHLVALACL-GGRPALDAVKKGLPHAPALIKRT

NRRIPERTSHRVADHAQVVRVLGFFQCHSHPAQAFDDAMTQFGMSRHGLLQLFRRVGVTELEARSGTLPPASQRWDRILQASGMKRAKPSPTSTQTPDQASLHAFADSLERDLDAPSPMHEGDQTRASSRKRSRSDRAVTGPSAQQSFEVRVPEQRDALHLPLSWRVKRPRTSIGGGLPDPGTPTAADLAASSTVMREQDEDPFAGAADDFPAFNEEELAWLMELLPQ

>Brg11 (from *Ralstonia solanacearum* strain GMI1000; GenBank NP_519936.1)

MRIGKSSGWLNESVSLEYEHVSPPTRPRDTRRRPRAAGDGGLAHLHRRLAVGYAEDTPRTEARSPAPRRPLPVAPASAPPAPSLVPEPPMPVSLPAVSSPRFSAGSSAAITDPFPSLPPTPVLYAMARELEALSDATWQPAVPLPAEPPTDARRGNTVFDEASASSPVIASACPQAFASPPRAPRSARARRARTGGDAWPAPTFLSRPSSSRIGRDVFGKLVALGYSREQIRKLKQESLSEIAKYHTTLTGQGFTHADICRISRRRQSLRVVARNYPELAAALPE

-1 LTRAHIVDIARQRSGDLALQALLPVATALTAAPLR

0 LSASQIATVAQY GERPAIQALYRLRRKLTRAPLH

1 LTPQQVVAIAS**NT**GGKRALEAVCVQLPVLRAAPYR

2 LSTEQVVAIAS**NK**GGKQALEAVKAHLLDLLGAPYV

3 LDTEQVVAIAS**HN**GGKQALEAVKADLLDLRGAPYA

4 LSTEQVVAIAS**HN**GGKQALEAVKADLLELRGAPYA

5 LSTEQVVAIAS**HN**GGKQALEAVKAHLLDLRGVPYA

6 LSTEQVVAIAS**HN**GGKQALEAVKAQLLDLRGAPYA

7 LSTAQVVAIAS**NG**GGKQALEGIGEQLLKLRTAPYG

8 LSTEQVVAIAS**HD**GGKQALEAVGAQLVALRAAPYA

9 LSTEQVVAIAS**NK**GGKQALEAVKAQLLELRGAPYA

10 LSTAQVVAIAS**HD**GGNQALEAVGTQLVALRAAPYA

11 LSTEQVVAIAS**HD**GGKQALEAVGAQLVALRAAPYA

12 LNTEQVVAIAS**SH**GGKQALEAVRALFPDLRAAPYA

13 LSTAQLVAIAS**NP**GGKQALEAVRALFRELRAAPYA

14 LSTEQVVAIAS**NH**GGKQALEAVRALFRGLRAAPYG

15 LSTAQVVAIAS**SN**GGKQALEAVWALLPVLRATPYD

16 LNTAQIVAIAS**HD**GGKPALEAVWAKLPVLRGAPYA

+1 LSTAQVVAIACI-SGQQALEAIEAHMPTLRQASHS

+2 LSPERVAAIACI-GGRSAVEAVRQGLPVKAIRRIRR

EKAPVAGPPPASLGPTPQELVAVLHFFRAHQQPRQAFVDALAAFQATRPALLRLLSSVGVTEIEALGGTIPDATERWQRLLGRLGFRPATGAAAPSPDSLQGFAQSLERTLGSPGMAGQSACSPHRKRPAETAIAPRSIRRSPNNAGQPSEPWPDQLAWLQRRKRTARSHIRADSAASVPANLHLGTRAQFTPDRLRAEPGPIMQAHTSPASVSFGSHVAFEPGLPDPGTPTSADLASFEAEPFGVGPLDFHLDWLLQILET

**Supplementary Figure 3:** Amino acid alignments of Bat2 and Bat3 core repeats.

Alignments of the core repeats of Bat2 and Bat3 were created in Clustal Omega (34, 35) and Boxshade was used for formatting. White lettering on a black background indicates a consensus residue. Black lettering on a grey background indicates a residue similar to the consensus residue. Black lettering on a white background indicates a residue neither identical nor similar to the consensus residue. Repeats are shown in order of appearance in the polypeptide and numbered accordingly. The consensus repeat is shown below each alignment.

>Alignment of Bat2 core repeats

01 FSRADIVRIAGNGGGAQALYSVLDVEPTLGKRG

02 FSQVDVVKIAG--GGAQALHTVLEIGPTLGERG

03 FSRGDIVTIAGNNGGAQALQAVLELEPTLRERG

04 FNQADIVKIAGNGGGAQALQAVLDVEPALGKRG

05 FSRVDIAKIA--GGGAQALQAVLGLEPTLRKRG

06 FHPTDIIKIAGNNGGAQALQAVLDLELMLRERG

07 FSQADIVKMASNIGGAQALQAVLNLEPALCERG

08 FSQPDIVKMAGNSGGAQALQAVLDLELAFRERG

09 FSQADIVKMASNIGGAQALQAVLELEPALHERG

10 FSQANIVKMAGNSGGAQALQAVLDLELVFRERG

11 VRQADIVKIVGNNGGAQALQAVFELEPTLRERG

12 FNQATIVKIAANGGGAQALYSVLDVEPTLDKRG

13 FSRVDIVKIAG--GGAQALHTAFELEPTLRKRG

14 FNPTDIVKIAGNKGGAQALQAVLELEPALRERG

15 FNQATIVKMAGNAGGAQALYSVLDVEPALRERG

16 FSQPEIVKIAGNIGGAQALHTVLELEPTLHKRG

17 FNPTDIVKIAGNSGGAQALQAVLELEPAFRERG

18 FGQPDIVKMASNIGGAQALQAVLELEPALRERG

19 FSQPDIVEMAGNIGGAQALQAVLELEPAFRERG

20 FSQSDIVKIAGNIGGAQALQAVLELEPTLRESD

21 FRQADIVNIAGNDGSTQALKAVIEHGPRLRQRG

22 FNRASIVKIAGNSGGAQALQAVLKHGPTLDERG

23 FNLTNIVKIAGNGGGAQALKAVIEHGPTLQQRG

24 FNLTDIVEMAGKGGGAQALKAVLEHGPTLRQRG

25 FNLIDIVEMASNTGGAQALKTVLEHGPTLRQRD

26 LSLIDIVEIASN-GGAQALKAVLKYGPVLMQAG

FSQADIVKIAGNGGGAQALQAVLELEPTLRERG

> Alignment of Bat3 core repeats

01 FARADIIKITGNGGGAQALKAVVVHGPTLNECG

02 FSQADIVRIADNIGGAQALKAVLEHGPTLNERD

03 YSGADIVKIAGNGGGARALKAVVMHGPTLCESG

04 YSGADIVKIASNGGGAQALEAVAMHGSTLCERG

05 YCRTDIAKIAGNGGGAQALKAIVMHGPTLCERG

06 YSRTDIVKIADNNGGAQALKAVFEHGPALTQAG

YSRADIVKIAGNGGGAQALKAVVMHGPTLCERG

**Supplementary Figure 4:** Nucleotide sequences of synthesised *Bat1, Bat2* and *Bat3* genes

Genes encoding the three predicted proteins were synthesised with *E. coli* codon usage (GenScript). Each was synthesised as a series of separate blocks flanked by BsaI sites allowing ordered assembly via BsaI cut-ligation into target vectors. BsaI recognition sites are underlined, while bold typeface marks the overlaps created upon digest. Start and stop codons are distinguished with the use of lowercase italics.

>Bat1 block 1

GGTCTCT**CACC***atg*AGCACCGCCTTCGTGGACCAAGATAAGCAAATGGCAAATCGCCTGAACCTGTCACCGCTGGAACGTAGCAAAATTGAAAAACAATATGGCGGTGCAACCACGCTGGCTTTTATTAGCAACAAACAGAATGAACTGGCACAAATCCTGAGCCGTGCTGATATTCTGAAAATCGCGTCTTACGACTGCGCAGCACATGCACTGCAGGCTGTCCTGGATTGTGGCCCGATGCTGGGCAAACGCGGTTTTAGCCAGTCTGACATTGTCAAGATCGCCGGTAACATTGGCGGTGCACAGGCACTGCAAGCAGTGCTGGATCTGGAAAGTATGCTGGGCAAACGTGGTTTCTCCCGCGATGACATTGCGAAGATGGCCGGCAATATCGGCGGTGCACAGACCCTGCAGGCCGTGCTGGATCTGGAATCAGCCTTTCGTGAACGCGGCTTCTCGCAGGCCGACATTGTTAAAATCGCCGGTAACAATGGCGGTGCACAAGCTCTGTATAGTGTGCTGGATGTTGAACCGACCCTGGGTAAACGTGGTTTTTCACGCGCTGACATTGTTAAGATCGCCGGTAACACCGGCGGTGCCCAAGCACTGCACACGGTCCTGGATCTGGAACCGGCCCTGGGCAAGCGTGGTTTCTCCCGCATTGATATCGTTAAGATCGCAGCTAACAACGGTGGTGCTCAAGCCCTGCACGCTGTCCTGGATCTGGGTCCGACGCTGCGCGAATG**TGGG**TGAGACC

>Bat1 block 2

GGTCTCT**TGGG**TTCTCGCAGGCAACCATCGCAAAAATCGCTGGCAATATCGGCGGTGCTCAGGCTCTGCAAATGGTGCTGGATCTGGGTCCGGCTCTGGGCAAACGTGGTTTTAGCCAGGCAACCATTGCTAAGATCGCCGGTAACATTGGCGGTGCACAGGCACTGCAAACGGTCCTGGATCTGGAACCGGCGCTGTGCGAACGCGGCTTCTCTCAGGCCACCATCGCAAAAATGGCTGGTAACAATGGCGGTGCACAGGCTCTGCAAACGGTTCTGGATCTGGAACCGGCCCTGCGTAAACGCGATTTTCGTCAGGCGGACATTATCAAGATTGCCGGTAATGACGGTGGCGCCCAGGCACTGCAAGCAGTGATCGAACATGGCCCGACCCTGCGCCAACACGGTTTCAACCTGGCAGACATTGTTAAGATGGCTGGTAATATCGGTGGTGCTCAAGCTCTGCAAGCGGTGCTGGACCTGAAGCCGGTGCTGGACGAACAT**GGTT**TGAGACC

>Bat1 block 3

GGTCTCT**GGTT**TCTCTCAACCGGATATCGTCAAGATGGCGGGCAACATTGGTGGTGCTCAAGCCCTGCAAGCCGTCCTGTCACTGGGTCCGGCGCTGCGTGAACGTGGCTTTAGCCAGCCGGATATTGTCAAAATCGCCGGTAACACCGGCGGTGCACAGGCACTGCAAGCAGTGCTGGATCTGGAACTGACGCTGGTTGAACATGGCTTCTCTCAACCGGACATTGTTCGCATCACCGGTAATCGTGGCGGTGCCCAAGCTCTGCAAGCGGTGCTGGCTCTGGAACTGACCCTGCGTGAACG**AGGA**TGAGACC

>Bat1 block 4

GGTCTCT**AGGA**TTTAGCCAACCGGACATCGTGAAAATCGCGGGCAATAGCGGCGGTGCTCAAGCTCTGCAAGCGGTCCTGGATCTGGAACTGACGTTTCGTGAACGCGGCTTTAGCCAGGCGGATATTGTCAAAATCGCCGGTAACGACGGCGGTACCCAAGCACTGCATGCTGTGCTGGATCTGGAACGTATGCTGGGCGAACGTGGTTTCTCTCGCGCAGACATTGTGAACGTTGCTGGCAACAATGGCGGTGCGCAGGCCCTGAAAGCCGTGCTGGAACACGAAGCCACGCTGAATGAACGTGGCTTTAGTCGCGCAGATATTGTCAAGATCGCGGGTAACGGTGGCGGCGCACAAGCACTGAAGGCGGTTCTGGAACACGAAGCGACCCTGGATGAACG**CGGC**TGAGACC

>Bat1 block 5

GGTCTCT**CGGC**TTTTCTCGTGCTGATATTGTCCGTATTGCGGGTAATGGTGGTGGTGCCCAGGCTCTGAAGGCTGTGCTGGAACATGGTCCGACGCTGAACGAACGTGGCTTTAATCTGACCGATATTGTTGAAATGGCGGCCAACAGTGGCGGTGCACAGGCTCTGAAAGCGGTCCTGGAACACGGCCCGACGCTGCGTCAACGTGGTCTGAGCCTGATTGACATCGTGGAAATTGCATCTAACGGCGGTGCGCAGGCCCTGAAAGCTGTCCTGAAGTATGGTCCGGTGCTGATGCAAGCAGGTCGTAGCAATGAAGAAATCGTGCACGTTGCCGCTCGTCGTGGTGGTGCTGGCCGTATCCGTAAGATGGTTGCTCCGCTGCTGGAACGTCAG*tag***AAGG**TGAGACC

>Bat2 block1

GGTCTCT**CACC***atg*CCGGCCACCTCGATGCACCAAGAAGATAAACAGTCCGCAAACGGTCTGAACCTGAGCCCGCTGGAACGTATTAAAATTGAAAAACATTATGGCGGTGGCGCGACCCTGGCCTTTATTAGTAACCAGCACGATGAACTGGCACAAGTGCTGAGCCGTGCTGACATTCTGAAAATCGCCTCTTATGACTGTGCTGCTCAAGCTCTGCAAGCGGTGCTGGACTGCGGCCCGATGCTGGGTAAACG**CGGC**TGAGACC

>Bat2 block2

GGTCTCT**CGGC**TTTTCCCGTGCTGATATTGTCCGTATTGCTGGTAATGGTGGTGGTGCCCAAGCTCTGTATTCTGTCCTGGATGTTGAACCGACGCTGGGTAAACGTGGCTTTAGCCAGGTTGATGTGGTTAAAATTGCGGGCGGTGGCGCACAAGCACTGCATACCGTCCTGGAAATCGGTCCGACGCTGGGTGAACGTGGCTTCTCTCGCGGTGACATTGTTACCATCGCCGGCAACAATGGTGGCGCACAGGCTCTGCAAGCAGTTCTGGAACTGGAACCGACGCTGCGTGAACGCGGTTTTAACCAGGCGGATATTGTCAAAATCGCCGGTAATGGTGGCGGTGCACAGGCACTGCAAGCAGTCCTGGATGTGGAACCGGCTCTGGGTAAACGTGGCTTTTCCCGCGTGGACATTGCAAAAATCGCTGGCGGTGGCGCCCAAGCCCTGCAGGCAGTTCTGGGTCTGGAACCGACCCTGCGTAAACGCGGCTTCCACCCGACGGACATTATCAAAATTGCGGGTAACAATGGTGGTGCCCAAGCACTGCAAGCAGTTCTGGATCTGGAACTGATGCTGCGTGAACGCGGCTTTAGCCAGGCAGACATTGTGAAAATGGCTTCTAACATCGGTGGCGCCCAAGCTCTGCAAGCGGTTCTGAATCTGGAACCGGCCCTGTGCGAACGCGGTTTCTCACAGCCGGATATCGTCAAAATGGCCGGTAACTCGGGTGGCGCCCAAGCGCTGCAAGCAGTGCTGGATCTGGAACTGGCTTTTCGTGAACGCGGCTTCAGTCAGGCGGACATTGTGAAAATGGCCTCCAATATCGGCGGCGCACAAGCACTGCAAGCTGTCCTGGAACTGGAACCGGCTCTGCACGAACGCGGCTT**TAGT**TGAGACC

>Bat2 block3

GGTCTCA**TAGT**CAAGCAAATATCGTCAAAATGGCGGGTAATAGTGGTGGTGCCCAAGCCCTGCAAGCGGTCCTGGATCTGGAACTGGTCTTTCGTGAACGTGGCGTGCGCCAGGCGGATATTGTGAAAATCGTTGGTAACAATGGCGGTGCACAGGCTCTGCAAGCAGTCTTTGAACTGGAACCGACCCTGCGTGAACGCGGCTTCAACCAGGCTACGATTGTTAAAATCGCAGCAAATGGCGGTGGCGCACAAGCACTGTATAGCGTCCTGGATGTGGAACCGACCCTGGACAAACGTGGTTTCTCTCGCGTTGATATTGTCAAAATCGCAGGTGGCGGTGCCCAAGCTCTGCATACCGCTTTTGAACTGGAACCGACGCTGCGTAAACGCGGCTTCAACCCGACCGACATTGTCAAAATCGCCGGTAATAAAGGCGGTGCACAGGCACTGCAAGCAGTGCTGGAACTGGAACCGGCTCTGCGTGAACGCGGCTTTAACCAGGCAACGATTGTGAAAATGGCGGGTAATGCCGGCGGTGCACAAGCTCTGTACAGTGTGCTGGATGTTGAACCGGCACTGCGTGAACGTGGTTTCTCCCAGCCGGAAATTGTTAAAATCGCCGGTAACATCGGCGGTGCGCAAGCCCTGCATACGGTTCTGGAGTTAGAACCGACCCTGCACAAACGTGGCTTTAACCCGACCGATATTGTGAAAATCGCGGGTAATAGCGGCGGTGCCCAGGCCCTGCAGGCGGTTCTGGAACTGGAACCGGCGTTTCGTGAACGCGGCTTCGGTCAGCCGGACATTGTTAAAATGGCCAGCAATATCGGCGGTGCCCAAGCCCTGCAAGCCGTCCTGGAACTGGAACCGGCCCTGCGTGAACGTGGTTTTAG**CCAG**TGAGACC

>Bat2 block4

GGTCTCT**CCAG**CCGGATATTGTGGAAATGGCGGGTAACATCGGCGGCGCTCAAGCCCTGCAAGCTGTCCTGGAACTGGAACCGGCCTTTCGTGAACGCGGCTTTAGCCAGTCTGATATTGTTAAAATCGCGGGTAACATTGGCGGTGCACAGGCACTGCAAGCAGTTCTGGAACTGGAACCGACCCTGCGCGAAAGCGATTTCCGTCAGGCAGACATTGTGAACATCGCTGGCAATGACGGTTCTACCCAAGCGCTGAAAGCCGTTATTGAACATGGCCCGCGTCTGCGCCAGCGTGGTTTTAACCGCGCGAGTATTGTCAAAATCGCCGGCAATTCCGGCGGTGCACAGGCTCTGCAAGCAGTGCTGAAACACGGCCCGACCCTGGATGAACGTGGTTTCAACCTGACGAATATTGTTAAAATCGCCGGTAACGGCGGTGGCGCACAGGCACTGAAAGCTGTCATTGAACATGGCCCGACCCTGCAGCAACGCGGTTTTAATCTGACGGATATCGTGGAAATGGCGGGCAAAGGTGGCGGTGCACAAGCTCTGAAAGCAGTTCTGGAACACGGTCCGACCCTGCGTCAGCGTGGTTTCAACCTGATTGACATCGTCGAAATGGCGTCCAATACGGGCGGTGCGCAAGCCCTGAAAACCGTTCTGGAACATGGTCCGACGCTGCGCCAGCGTGATCTGTCACTGATTGACATCGTGGAAATTGCATCGAATGGTGGTGCACAGGCTCTGAAAGCTGTCCTGAAATATGGCCCGGTGCTGATGCAGGCAGGTCGTAGCAATGAAGAAATCGTGCACGTTGCCGCTCGTCGTGGTGGTGCGGGCCGTATTCGTAAAATGGTTGCTCTGCTGCTGGAACGCCAA*t****aa*GG**TGAGACC

>Bat3 block 1

GGTCTCT**CACC**ATGCCGGTCACCAGCGTCTACCAAAAAGATAAACCGTTCGGCGCACGTCTGAACCTGAGCCCGTTTGAATGTCTGAAAATTGAAAAACATAGCGGCGGTGCGGATGCCCTGGAATTTATTTCTAACAAATATGACGCCCTGACCCAGGTGCTGAGTCGTGCAGATATTCTGAAAATCGCTTGCCACGACTGTGCCGCCCACGCTCTGCAAGCTGTGCTGGACTATGAACAAGTTTTTCGCCAACG**CGGC**TGAGACC

>Bat3 block 2

GGTCTCT**CGGC**TTCGCTCGTGCAGATATTATTAAAATCACGGGTAACGGCGGTGGTGCCCAAGCCCTGAAAGCAGTGGTTGTCCATGGTCCGACGCTGAACGAATGCGGTTTTTCACAGGCGGATATTGTCCGTATCGCCGACAATATTGGCGGTGCGCAAGCCCTGAAAGCGGTGCTGGAACATGGCCCGACCCTGAACGAACGTGATTATTCGGGTGCAGACATTGTGAAAATCGCTGGTAATGGCGGTGGCGCACGTGCTCTGAAAGCAGTGGTTATGCACGGTCCGACGCTGTGTGAAAGCGGTTACTCTGGCGCGGATATTGTTAAAATCGCAAGTAACGGTGGCGGTGCACAGGCACTGGAAGCAGTCGCTATGCATGGTTCCACCCTGTGCGAACGTGGCTATTGTCGCACGGACATTGCGAAAATCGCCGGCAACGGCGGTGGCGCACAAGCACTGAAAGCAATTGTCATGCACGGTCCGACCCTGTGTGAACGCGGCTACAGCCGCACGGATATTGTGAAAATCGCAGACAACAATGGTGGCGCACAGGCTCTGAAAGCTGTTTTCGAACATGGTCCGGCACTGACCCAAGCTGGCCGCAGTAACGAAGATATCGTTAATATGGCCGCACGCACGGGCGCAGCGGGTCAGATTCGTAAAATGGCGGCACAACTGTCGGGTCGTCAA*t****aa*GG**TGAGACC

**Supplementary Figure 5:** Sequences of translational fusions for protein purification, transcriptional activation reporters and nuclease assay.

Only the sequences specific to each expression construct are shown. The sequence of the relevant Bat protein or derivative or TALE derivative fills the position indicated. Epitopes used for purification or antibody binding are indicated with a red background. NLSs are indicated with a yellow background. Green background marks an activation domain and mustard-brown a nuclease domain.

>Protein expression and purification

MSYYHHHHHHLESTSLYKKAGSAAAPFT – Bat1, Bat2 or Bat3 coding sequence – STOP

>Human cell transcriptional activation assay: Full construct

MGYPYDVPDYASRPKKKRKVGIHAM – Bat1, dBat or dTALE coding sequence -

GGGGGGSGGGGSGGGGSDYKDHDGDYKDHDIDYKDDDDKGSSPKKKRKVEASGSGRADALDDFDLDMLGSDALDDFDLDMLGSDALDDFDLDMLGSDALDDFDLDMLINSR – STOP

>Human cell transcriptional activation assay: ΔAD

MGYPYDVPDYASRPKKKRKVGIHAM – Bat1 coding sequence -

GGGGGGSGGGGSGGGGSDYKDHDGDYKDHDIDYKDDDDKGSSPKKKRKVEAS – STOP

>Human cell transcriptional activation assay: ΔNLSs

START – Bat1 coding sequence -

GGGGGGSGGGGSGGGGSDYKDHDGDYKDHDIDYKDDDDKGSGRADALDDFDLDMLGSDALDDFDLDMLGSDALDDFDLDMLGSDALDDFDLDMLINSR – STOP

>*Planta* transcriptional activation assay: Full construct

START – Bat1 or dTALE coding sequence -

GGGGGGSGGGGSGGGGSDYKDHDGDYKDHDIDYKDDDDKGSSPKKKRKVEASGSGRADALDDFDLDMLGSDALDDFDLDMLGSDALDDFDLDMLGSDALDDFDLDMLINSR – STOP

>*Planta* transcriptional activation assay: ΔAD

START – Bat1 coding sequence -

GGGGGGSGGGGSGGGGSDYKDHDGDYKDHDIDYKDDDDKGSSPKKKRKVEAS – STOP

>*In vitro* nuclease assay

MGLINIFYPYDVPDYAGYPYDVPDYAGSYPYDVPDYAAQCSG – Bat1 coding sequence –

GGQLVKSELEEKKSELRHKLKYVPHEYIELIEIARNSTQDRILEMKVMEFFMKVYGYRGKHLGGSRKPDGAIYTVGSPIDYGVIVDTKAYSGGYNLPIGQADEMQRYVEENQTRNKHINPNEWWKVYPSSVTEFKFLFVSGHFKGNYKAQLTRLNHITNCNGAVLSVEELLIGGEMIKAGTLTLEEVRRKFNNGEINF

MGYPYDVPDYASRPKKKRKVGIHAS – TALE coding sequence -

GSQLVKSELEEKKSELRHKLKYVPHEYIELIEIARNSTQDRILEMKVMEFFMKVYGYRGKHLGGSRKPDGAIYTVGSPIDYGVIVDTKAYSGGYNLPIGQADEMQRYVEENQTRNKHINPNEWWKVYPSSVTEFKFLFVSGHFKGNYKAQLTRLNHITNCNGAVLSVEELLIGGEMIKAGTLTLEEVRRKFNNGEINF

**Supplementary Figure 6:** Target and reporter sequences used in this study.

**Sequences of binding elements** used for electrophoretic mobility shift assays (Figure 2). Only forward strand shown, the binding element is highlighted with bold lettering

BE_Bat1_/BE_Bat1 T-0_

TAGACT**AAGAGAAGCAAAGACGTTAT**ATGC

BE_Bat2_

TAGACT**TTGTTGAAAAGTTGTAAAAACATTAT**ATGC

BE_Bat3_

TAGACATAGATTAT**TATATTTG**TAACAAGTAAATGC

BE_Bat1 C-0_

TAGACC**AAGAGAAGCAAAGACGTTAT**ATGC

BE_Bat1 G-0_

TAGACG**AAGAGAAGCAAAGACGTTAT**ATGC

BE_Bat1 A-0_

TAGACA**AAGAGAAGCAAAGACGTTAT**ATGC

**Sequences of reporters and binding elements used in assessments of transcriptional activation (Figures 3, 5-7 and S8)**

**pCMV-*BE-dsEGFP*** – transcriptional activation reporter in human cells. (Figures 3, 5-7, S8). Green highlighting is used for the dsEGFP coding sequence and italics for the subsequence polyA signal. Grey highlighting for the minimal CMV promoter. The bold-N positions are filled by one of the four binding elements listed.

BE_Bat1_ AAGAGAAGCAAAGACGTTAT

BE_dBatRVDswitch1_ A**GAGA**AAGCAAAGACGTTAT

BE_dBatRVDswitch2_ AAGAGA**GCA**AAAGACGTTAT

BE_pSOX2_ TTTATTCCCTGACAGCCCC

CTAGACT**NNNNNNNNNNNNNNNNNNNNN**ATGCGGATCCACGTATGTCGAGGTAGGCGTGTACGGTGGGAGGCCTATATAAGCAGAGCTCGTTTAGTGAACCGTCAGATCGCCTGGAGGTACCGCCACCATGGGCTTAATTAATATAATTAATAATCCACTTAAGAATTCTTTAAAGTGGATTATTAATTATAGGACCGGTATTACCCTGTTATCCCTAGTGAGCAAGGGCGAGGAGCTGTTCACCGGGGTGGTGCCCATCCTGGTCGAGCTGGACGGCGACGTAAACGGCCACAAGTTCAGCGTGTCCGGCGAGGGCGAGGGCGATGCCACCTACGGCAAGCTGACCCTGAAGTTCATCTGCACCACCGGCAAGCTGCCCGTGCCCTGGCCCACCCTCGTGACCACCCTGACCTACGGCGTGCAGTGCTTCAGCCGCTACCCCGACCACATGAAGCAGCACGACTTCTTCAAGTCCGCCATGCCCGAAGGCTACGTCCAGGAGCGCACCATCTTCTTCAAGGACGACGGCAACTACAAGACCCGCGCCGAGGTGAAGTTCGAGGGCGACACCCTGGTGAACCGCATCGAGCTGAAGGGCATCGACTTCAAGGAGGACGGCAACATCCTGGGGCACAAGCTGGAGTACAACTACAACAGCCACAACGTCTATATCATGGCCGACAAGCAGAAGAACGGCATCAAGGTGAACTTCAAGATCCGCCACAACATCGAGGACGGCAGCGTGCAGCTCGCCGACCACTACCAGCAGAACACCCCCATCGGCGACGGCCCCGTGCTGCTGCCCGACAACCACTACCTGAGCACCCAGTCCGCCCTGAGCAAAGACCCCAACGAGAAGCGCGATCACATGGTCCTGCTGGAGTTCGTGACCGCCGCCGGGATCACTCTCGGCATGGACGAGCTGTACAAGAAGCTTAGCCATGGCTTCCCGCCGGAGGTGGAGGAGCAGGATGATGGCACGCTGCCCATGTCTTGTGCCCAGGAGAGCGGGATGGACCGTCACCCTGCAGCCTGTGCTTCTGCTAGGATCAATGTGTAGCTAAGTAAGATCC*TTCGAGCAGACATGATAAGATACATTGATGAGTTTGGACAAACCACAACTAGAATGCAGTGAAAAAAATGCTTTATTTGTGAAATTTGTGATGCTATTGCTTTATTTGTAACCATTATAAGCTGCAATAAACAAGTTAACAACAACAATTGCATTCATTTTATGTTTCAGGTTCAGGGGGAGGTGTGGGAGGTTTTTTAAAGCAAGTAAAACCTCTACAAATGTGGTAAAA*

**Bs3p-BE_Bat1-_*uidA*** for *in planta* assessment of transcriptional activation (Figure S8). Blue indicates the coding sequence of the *uidA* reporter gene, which is a part of the vector pGWB3* (10). BE_Bat1_ is embedded within the pepper *Bs3* promoter (italics) and is distinguished with bold typeface. In this construct a guanine base is paired with the 20th repeat of acBat1 and dTALE_Bat1mimic_.

*TCATAGTCAAGCTAACGAAACTTATGCAAGGGAAATATGAAATTAGTATGCAAGTAAACTCAAAGAACTAATCATTGAACTGAAAGATCAATATATCAAAAAAAAAAAAAAAACAATAAAACCGTTTAACCGATAGATTAACCATTTCTGGTTCAGTTTATGGGTTAAACCACAATTTGCACACCCTGGTTAAACAATGAACACGTTTGCCTGACCAATTTTATTATATAAACCTAACCATCCTCACAACT****AAGAGAAGCAAAGACGTTAG****GTTCAAGTTATCATCCCCTTTCTCTTTTCTCCTCTTGTTCTTGTCACCCGCTAAATCTATCAAAACACAAGTAGTCCTAGTTGCACTATATTTC*AAGGGTGGGCGCGCCGACCCAGCTTTCTTGTACAAAGTGGTTCGATCTAGAGGATCCCCGGGTGGTCAGTCCCTTATGTTACGTCCTGTAGAAACCCCAACCCGTGAAATCAAAAAACTCGACGGCCTGTGGGCATTCAGTCTGGATCGCGAAAACTGTGGAATTGATCAGCGTTGGTGGGAAAGCGCGTTACAAGAAAGCCGGGCAATTGCTGTGCCAGGCAGTTTTAACGATCAGTTCGCCGATGCAGATATTCGTAATTATGCGGGCAACGTCTGGTATCAGCGCGAAGTCTTTATACCGAAAGGTTGGGCAGGCCAGCGTATCGTGCTGCGTTTCGATGCGGTCACTCATTACGGCAAAGTGTGGGTCAATAATCAGGAAGTGATGGAGCATCAGGGCGGCTATACGCCATTTGAAGCCGATGTCACGCCGTATGTTATTGCCGGGAAAAGTGTACGTATCACCGTTTGTGTGAACAACGAACTGAACTGGCAGACTATCCCGCCGGGAATGGTGATTACCGACGAAAACGGCAAGAAAAAGCAGTCTTACTTCCATGATTTCTTTAACTATGCCGGAATCCATCGCAGCGTAATGCTCTACACCACGCCGAACACCTGGGTGGACGATATCACCGTGGTGACGCATGTCGCGCAAGACTGTAACCACGCGTCTGTTGACTGGCAGGTGGTGGCCAATGGTGATGTCAGCGTTGAACTGCGTGATGCGGATCAACAGGTGGTTGCAACTGGACAAGGCACTAGCGGGACTTTGCAAGTGGTGAATCCGCACCTCTGGCAACCGGGTGAAGGTTATCTCTATGAACTGTGCGTCACAGCCAAAAGCCAGACAGAGTGTGATATCTACCCGCTTCGCGTCGGCATCCGGTCAGTGGCAGTGAAGGGCGAACAGTTCCTGATTAACCACAAACCGTTCTACTTTACTGGCTTTGGTCGTCATGAAGATGCGGACTTGCGTGGCAAAGGATTCGATAACGTGCTGATGGTGCACGACCACGCATTAATGGACTGGATTGGGGCCAACTCCTACCGTACCTCGCATTACCCTTACGCTGAAGAGATGCTCGACTGGGCAGATGAACATGGCATCGTGGTGATTGATGAAACTGCTGCTGTCGGCTTTAACCTCTCTTTAGGCATTGGTTTCGAAGCGGGCAACAAGCCGAAAGAACTGTACAGCGAAGAGGCAGTCAACGGGGAAACTCAGCAAGCGCACTTACAGGCGATTAAAGAGCTGATAGCGCGTGACAAAAACCACCCAAGCGTGGTGATGTGGAGTATTGCCAACGAACCGGATACCCGTCCGCAAGGTGCACGGGAATATTTCGCGCCACTGGCGGAAGCAACGCGTAAACTCGACCCGACGCGTCCGATCACCTGCGTCAATGTAATGTTCTGCGACGCTCACACCGATACCATCAGCGATCTCTTTGATGTGCTGTGCCTGAACCGTTATTACGGATGGTATGTCCAAAGCGGCGATTTGGAAACGGCAGAGAAGGTACTGGAAAAAGAACTTCTGGCCTGGCAGGAGAAACTGCATCAGCCGATTATCATCACCGAATACGGCGTGGATACGTTAGCCGGGCTGCACTCAATGTACACCGACATGTGGAGTGAAGAGTATCAGTGTGCATGGCTGGATATGTATCACCGCGTCTTTGATCGCGTCAGCGCCGTCGTCGGTGAACAGGTATGGAATTTCGCCGATTTTGCGACCTCGCAAGGCATATTGCGCGTTGGCGGTAACAAGAAAGGGATCTTCACTCGCGACCGCAAACCGAAGTCGGCGGCTTTTCTGCTGCAAAAACGCTGGACTGGCATGAACTTCGGTGAAAAACCGCAGCAGGGAGGCAAACAATGA

**Sequences of the PCR templates** used to create targets for the nuclease assays shown in Figure 4. Only the forward strand is shown. Grey highlighting shows the annealing sites for the amplification primers used in the PCR to create the target DNA for the nuclease assays. The two copies of BE_Bat1_ in reverse orientation are underlined. The italicised bases are one of the five spacers listed below. The entire yellow‑highlighted region is replaced by the given sequence in the case of the ‘no target’ control.

5bp CTAGC

7bp TCTAGAC

11bp TACGTCTAGAC

15bp TACGTACGTCTAGAC

19bp AAGCTACGTACGTCTAGAC

No target ATTGCCACGGCGACTCTCTTG

GCAGCTCCCGGAGACGGTCACAGCTTGTCTGTAAGCGGATGCCGGGAGCAGACAAGCCCGTCAGGGCGCGTCAGCGGGTGTTGGCGGGTGTCGGGGCTGGCTTAACTATGCGGCATCAGAGCAGATTGTACTGAGAGTGCACCATATGCGGTGTGAAATACCGCACAGATGCGTAAGGAGAAAATACCGCATCAGGCGCCATTCGCCATTCAGGCTGCGCAACTGTTGGGAAGGGCGATCGGTGCGGGCCTCTTCGCTATTACGCCAGCTGGCGAAAGGGGGATGTGCTGCAAGGCGATTAAGTTGGGTAACGCCAGGGTTTTCCCAGTCACGACGTTGTAAAACGACGGCCAGTGAATTCGAGCTCGGTACCTCGCGAATGCATCTAGATATCGGATCCCGGGCCCGTCGACTGCAGAGGGGTCTCCCCTTGAAATATAGTGCAACTAGGACTACTTGTGTTTTGATAGATTTAGCGGGTGACAAGAACAAGAGGAGAAAAGAGAAAGGGGATGATAACTTGAATAAGAGAAGCAAAGACGTTAT*NNNNNNNNNNNNN*ATAACGTCTTTGCTTCTCTTAGTTGTGAGGATGGTTAGGTTTATATAATAAAATTGGTCAGGCAAACGTGTTCATTGTTTAACCAGGGTGTGCAAATTGTGGTTTAACCCATAAACTGAACCAGAAATGGTTAATCTATCGGTTAAACGGTTTTATTGTTTTTTTTTTTTTTTGATATATTGATCTTTCAGTTCAATGATTAGTTCTTTGAGTTTACTTGCATACTAATTTCATATTTCCCTTGCATAAGTTTCGTTAGCTTGACTATGAGGTGGGAGACCCCTGCATGCAAGCTTGGCGTAATCATGGTCATAGCTGTTTCCTGTGTGAAATTGTTATCCGCTCACAATTCCACACAACATACGAGCCGGAAGCATAAAGTGTAAAGCCTGGGGTGCCTAATGAGTGAGCTAAC

**Supplementary Figure 7** – MST results for Bat1 measured against BE_Bat1A-0_,-_C-0_,-_G-0_, and-_T-0_

**Supplementary Figure 8:** Amino acid sequence of dTALEs used in this study

Core and cryptic repeats are numbered. Grey background and bold typeface highlight the RVD residues. In all cases only the TALE-derived amino acids are shown. The sequences of fused domains are given in Figure S5.

>dTALE_Bat1mimic_ (for transcriptional activation assays)

MDLRTLGYSQQQQEKIKPKVRSTVAQHHEALVGH

GFTHAHIVALSQHPAALGTVAVKYQDMIAALPE

-1 ATHEAIVGVGKQWSGARALEALLTVAGELRGPPLQ

0 LDTGQLLKIAKRGGVTAVEAVHAWRNALTGAPLN

1 LTPQQVVAIAS**NI**GGKQALETVQRLLPVLCQAHG

2 LTPEQVVAIAS**NI**GGKQALETVQRLLPVLCQAHG

3 LTPEQXVAIAS**NN**GGKQALXTVQRLLPVLCQAHG

4 LTPQQVVAIAS**NT**GGKQALXTVQRLLPVLCQAHG

5 LTPQQVVAIAS**NN**GGKQALETVQRLLPVLCQAHG

6 LTPEQVVAIAS**NI**GGKQALETVQRLLPVLCQAHG

7 LTPEQVVAIAS**NI**GGKQALETVQRLLPVLCQAHG

8 LTPEQVVAIAS**NN**GGKQALETVQRLLPVLCQAHG

9 LTPEQVVAIAS**ND**GGKQALETVQRLLPVLCQAHG

10 LTPEQVVAIAS**NI**GGKQALETVQRLLPVLCQAHG

11 LTPEQVVAIAS**NI**GGKQALETVQRLLPVLCQAHG

12 LTPQQVVAIAS**NT**GGKQALETVQALLPVLCQAHG

13 LTPQQVVAIAS**NR**GGKQALETVQRLLPVLCQAHG

14 LTPEQVVAIAS**NS**GGKQALETVQRLLPVLCQAHG

15 LTPEQVVAIAS**ND**GGKQALETVQRLLPVLCQAHG

16 LTPEQVVAIAS**NN**GGKQALETVQRLLPVLCQAHG

17 LTPEQVVAIAS**NG**GGKQALETVQRLLPVLCQAHG

18 LTPEQVVAIAS**NG**GGKQALETVQRLLPVLCQAHG

19 LTPQQVVAIAS**NS**GGKQALETVQALLPVLCQAHG

+1 LTPQQVVAIAS**N-**GGRPALESIVAQLSRPDPALAA

+2 LTNDHLVALACL-GGRPALDAVKKGLPHAPALIKR

TNRRIPERTSHRVA

>dTALE_SOX2_ (for human cell transcriptional activation assay)

MDLRTLGYSQQQQEKIKPKVRSTVAQHHEALVGH

GFTHAHIVALSQHPAALGTVAVKYQDMIAALPE

-1 ATHEAIVGVGKQWSGARALEALLTVAGELRGPPLQ

0 LDTGQLLKIAKRGGVTAVEAVHAWRNALTGAPLN

1 LTPQQVVAIAS**NI**GGKQALETVQRLLPVLCQAHG

2 LTPEQVVAIAS**NI**GGKQALETVQRLLPVLCQAHG

3 LTPEQXVAIAS**NN**GGKQALXTVQRLLPVLCQAHG

4 LTPQQVVAIAS**NT**GGKQALXTVQRLLPVLCQAHG

5 LTPQQVVAIAS**NN**GGKQALETVQRLLPVLCQAHG

6 LTPEQVVAIAS**NI**GGKQALETVQRLLPVLCQAHG

7 LTPEQVVAIAS**NI**GGKQALETVQRLLPVLCQAHG

8 LTPEQVVAIAS**NN**GGKQALETVQRLLPVLCQAHG

9 LTPEQVVAIAS**ND**GGKQALETVQRLLPVLCQAHG

10 LTPEQVVAIAS**NI**GGKQALETVQRLLPVLCQAHG

11 LTPEQVVAIAS**NI**GGKQALETVQRLLPVLCQAHG

12 LTPQQVVAIAS**NT**GGKQALETVQALLPVLCQAHG

13 LTPQQVVAIAS**NR**GGKQALETVQRLLPVLCQAHG

14 LTPEQVVAIAS**NS**GGKQALETVQRLLPVLCQAHG

15 LTPEQVVAIAS**ND**GGKQALETVQRLLPVLCQAHG

16 LTPEQVVAIAS**NN**GGKQALETVQRLLPVLCQAHG

17 LTPEQVVAIAS**NG**GGKQALETVQRLLPVLCQAHG

+1 LTPQQVVAIAS**N-**GGRPALESIVAQLSRPDPALAA

+2 LTNDHLVALACL-GGRPALDAVKKGLPHAPALIKR

TNRRIPERTSHRVA

>dTALE_Bat1mimic_ (for nuclease assay)

MAPRRRAAQPSDASPAAQVDLRTLGYSQQQQEKIKPKVRSTVAQHHEALVGH

GFTHAHIVALSQHPAALGTVAVKYQDMIAALPE

-1 ATHEAIVGVGKQWSGARALEALLTVAGELRGPPLQ

0 LDTGQLLKIAKRGGVTAVEAVHAWRNALTGAPLN

1 LTPQQVVAIAS**NI**GGKQALETVQRLLPVLCQAHG

2 LTPEQVVAIAS**NI**GGKQALETVQRLLPVLCQAHG

3 LTPEQXVAIAS**NN**GGKQALXTVQRLLPVLCQAHG

4 LTPQQVVAIAS**NT**GGKQALXTVQRLLPVLCQAHG

5 LTPQQVVAIAS**NN**GGKQALETVQRLLPVLCQAHG

6 LTPEQVVAIAS**NI**GGKQALETVQRLLPVLCQAHG

7 LTPEQVVAIAS**NI**GGKQALETVQRLLPVLCQAHG

8 LTPEQVVAIAS**NN**GGKQALETVQRLLPVLCQAHG

9 LTPEQVVAIAS**ND**GGKQALETVQRLLPVLCQAHG

10 LTPEQVVAIAS**NI**GGKQALETVQRLLPVLCQAHG

11 LTPEQVVAIAS**NI**GGKQALETVQRLLPVLCQAHG

12 LTPQQVVAIAS**NT**GGKQALETVQALLPVLCQAHG

13 LTPQQVVAIAS**NR**GGKQALETVQRLLPVLCQAHG

14 LTPEQVVAIAS**NS**GGKQALETVQRLLPVLCQAHG

15 LTPEQVVAIAS**ND**GGKQALETVQRLLPVLCQAHG

16 LTPEQVVAIAS**NN**GGKQALETVQRLLPVLCQAHG

17 LTPEQVVAIAS**NG**GGKQALETVQRLLPVLCQAHG

18 LTPEQVVAIAS**NG**GGKQALETVQRLLPVLCQAHG

19 LTPQQVVAIAS**NS**GGKQALETVQALLPVLCQAHG

+1 LTPQQVVAIAS**N-**GGRPALESIVAQLSRPDPALAA

+2 LT

**Supplementary Figure 9:** *in planta* transcriptional activation mediated by acBat1.

BE_Bat1_ was embedded within a 360 base pair fragment of the silent pepper *Bs3* promoter, using the primers listed in Table S2. This promoter derivative was then inserted upstream of *uidA* in the binary vector pGWB3* as previously described (10). Bat1 and TALE derivatives were assembled via BsaI cut-ligation along with the NLSs and VP64 activation domain (Figure S5) into pENTR/D-TOPO (Life technologies) derivatives bearing BsaI sites. They were then transferred to binary vector pGWB442 via LR recombination (Life technologies). *Agrobacterium tumefaciens* strains carrying pGWB442acBat1, pGWB442acBat1ΔAD or pGWB442dTALE_Bat1mimic_ were co-delivered into *Nicotiana benthamiana* leaves alongside a strain carrying the target reporter. In addition the reporter plasmid was delivered alone as a control. The target reporter was a promoter bearing BE_Bat1_ upstream of a *uidA* reporter gene (Figure S6). Leaf discs were harvested after 48 hours and GUS activity quantified (10). Results are shown for three biological replicates with error bars indicating standard deviation.


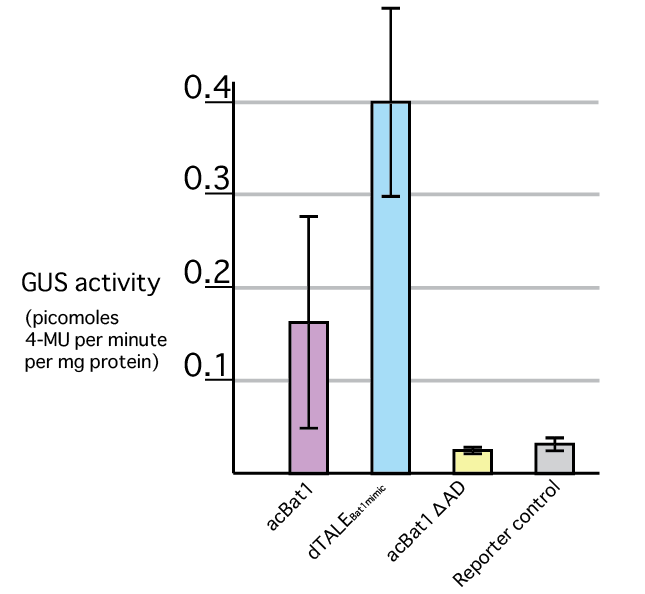


**Supplementary Figure 10:** Amino acid sequences of all acBat1 derivatives (dBats) tested in figures 5 and 6.

Dashes indicate truncated residues. Red font is used to highlight residues truncated or rearranged in each case. In all cases repeat numbering is used to identify repeats with those in the wild-type Bat1 protein. Grey background and bold typeface highlights the RVD residues. NND stands for non-repetitive N-terminal Domain. In all cases only the Bat1-derived amino-acids are shown. The sequences of fused domains are given in Figure S5.

**>**acBat1 Δ18-20

NND MSTAFVDQDKQMANRLN

-1 LSPLERSKIEKQYGGATTLAFISNKQNELAQI

0 LSRADILKIASYDCAAHALQAVLDCGPMLGKRG

1 FSQSDIVKIAG**NI**GGAQALQAVLDLESMLGKRG

2 FSRDDIAKMAG**NI**GGAQTLQAVLDLESAFRERG

3 FSQADIVKIAG**NN**GGAQALYSVLDVEPTLGKRG

4 FSRADIVKIAG**NT**GGAQALHTVLDLEPALGKRG

5 FSRIDIVKIAA**NN**GGAQALHAVLDLGPTLRECG

6 FSQATIAKIAG**NI**GGAQALQMVLDLGPALGKRG

7 FSQATIAKIAG**NI**GGAQALQTVLDLEPALCERG

8 FSQATIAKMAG**NN**GGAQALQTVLDLEPALRKRD

9 FRQADIIKIAG**ND**GGAQALQAVIEHGPTLRQHG

10 FNLADIVKMAG**NI**GGAQALQAVLDLKPVLDEHG

11 FSQPDIVKMAG**NI**GGAQALQAVLSLGPALRERG

12 FSQPDIVKIAG**NT**GGAQALQAVLDLELTLVEHG

13 FSQPDIVRITG**NR**GGAQALQAVLALELTLRERG

14 FSQPDIVKIAG**NS**GGAQALQAVLDLELTFRERG

15 FSQADIVKIAG**ND**GGTQALHAVLDLERMLGERG

16 FSRADIVNVAG**NN**GGAQALKAVLEHEATLNERG

17 FSRADIVKIAG**NG**GGAQALKAVLEHEATLDERG

18 FSRADIVRIAG**NG**GGAQ----------------

19 ---------------------------------

20 -----------------ALKAVLKYGPVLMQAG

+1 RSNEEIVHVAARRGGAGRIRKMVAP---LLERQ

**>**acBat1 Δ16-20

NNDMSTAFVDQDKQMANRLN

-1 LSPLERSKIEKQYGGATTLAFISNKQNELAQI

0 LSRADILKIASYDCAAHALQAVLDCGPMLGKRG

1 FSQSDIVKIAG**NI**GGAQALQAVLDLESMLGKRG

2 FSRDDIAKMAG**NI**GGAQTLQAVLDLESAFRERG

3 FSQADIVKIAG**NN**GGAQALYSVLDVEPTLGKRG

4 FSRADIVKIAG**NT**GGAQALHTVLDLEPALGKRG

5 FSRIDIVKIAA**NN**GGAQALHAVLDLGPTLRECG

6 FSQATIAKIAG**NI**GGAQALQMVLDLGPALGKRG

7 FSQATIAKIAG**NI**GGAQALQTVLDLEPALCERG

8 FSQATIAKMAG**NN**GGAQALQTVLDLEPALRKRD

9 FRQADIIKIAG**ND**GGAQALQAVIEHGPTLRQHG

10 FNLADIVKMAG**NI**GGAQALQAVLDLKPVLDEHG

11 FSQPDIVKMAG**NI**GGAQALQAVLSLGPALRERG

12 FSQPDIVKIAG**NT**GGAQALQAVLDLELTLVEHG

13 FSQPDIVRITG**NR**GGAQALQAVLALELTLRERG

14 FSQPDIVKIAG**NS**GGAQALQAVLDLELTFRERG

15 FSQADIVKIAG**ND**GGTQALHAVLDLERMLGERG

16 FSRADIVNVAG**NN**GGAQ----------------

17 ---------------------------------

18 ---------------------------------

19 ---------------------------------

20 ------------------ALKAVLKYGPVLMQAG

+1 RSNEEIVHVAARRGGAGRIRKMVAP---LLERQ

**>**acBat1 Δ14-20

NND MSTAFVDQDKQMANRLN

-1 LSPLERSKIEKQYGGATTLAFISNKQNELAQI

0 LSRADILKIASYDCAAHALQAVLDCGPMLGKRG

1 FSQSDIVKIAG**NI**GGAQALQAVLDLESMLGKRG

2 FSRDDIAKMAG**NI**GGAQTLQAVLDLESAFRERG

3 FSQADIVKIAG**NN**GGAQALYSVLDVEPTLGKRG

4 FSRADIVKIAG**NT**GGAQALHTVLDLEPALGKRG

5 FSRIDIVKIAA**NN**GGAQALHAVLDLGPTLRECG

6 FSQATIAKIAG**NI**GGAQALQMVLDLGPALGKRG

7 FSQATIAKIAG**NI**GGAQALQTVLDLEPALCERG

8 FSQATIAKMAG**NN**GGAQALQTVLDLEPALRKRD

9 FRQADIIKIAG**ND**GGAQALQAVIEHGPTLRQHG

10 FNLADIVKMAG**NI**GGAQALQAVLDLKPVLDEHG

11 FSQPDIVKMAG**NI**GGAQALQAVLSLGPALRERG

12 FSQPDIVKIAG**NT**GGAQALQAVLDLELTLVEHG

13 FSQPDIVRITG**NR**GGAQALQAVLALELTLRERG

14 FSQPDIVKIAG**NS**--------------------

15 ---------------------------------

16 ---------------------------------

17 ---------------------------------

18 ---------------------------------

19 ---------------------------------

20 -------------GGAQALKAVLKYGPVLMQAG

+1 RSNEEIVHVAARRGGAGRIRKMVAP---LLERQ

**>**acBat1 Δ12-20

NND MSTAFVDQDKQMANRLN

-1 LSPLERSKIEKQYGGATTLAFISNKQNELAQI

0 LSRADILKIASYDCAAHALQAVLDCGPMLGKRG

1 FSQSDIVKIAG**NI**GGAQALQAVLDLESMLGKRG

2 FSRDDIAKMAG**NI**GGAQTLQAVLDLESAFRERG

3 FSQADIVKIAG**NN**GGAQALYSVLDVEPTLGKRG

4 FSRADIVKIAG**NT**GGAQALHTVLDLEPALGKRG

5 FSRIDIVKIAA**NN**GGAQALHAVLDLGPTLRECG

6 FSQATIAKIAG**NI**GGAQALQMVLDLGPALGKRG

7 FSQATIAKIAG**NI**GGAQALQTVLDLEPALCERG

8 FSQATIAKMAG**NN**GGAQALQTVLDLEPALRKRD

9 FRQADIIKIAG**ND**GGAQALQAVIEHGPTLRQHG

10 FNLADIVKMAG**NI**GGAQALQAVLDLKPVLDEHG

11 FSQPDIVKMAG**NI**GGAQALQAVLSLGPALRERG

12 FSQPDIVKIAG**NT**--------------------

13 ---------------------------------

14 ---------------------------------

15 ---------------------------------

16 ---------------------------------

17 ---------------------------------

18 ---------------------------------

19 ---------------------------------

20 -------------GGAQALKAVLKYGPVLMQAG

+1 RSNEEIVHVAARRGGAGRIRKMVAP---LLERQ

**>**acBat1 ΔNTD

NND

-1

0

1 FSQSDIVKIAG**NI**GGAQALQAVLDLESMLGKRG

2 FSRDDIAKMAG**NI**GGAQTLQAVLDLESAFRERG

3 FSQADIVKIAG**NN**GGAQALYSVLDVEPTLGKRG

4 FSRADIVKIAG**NT**GGAQALHTVLDLEPALGKRG

5 FSRIDIVKIAA**NN**GGAQALHAVLDLGPTLRECG

6 FSQATIAKIAG**NI**GGAQALQMVLDLGPALGKRG

7 FSQATIAKIAG**NI**GGAQALQTVLDLEPALCERG

8 FSQATIAKMAG**NN**GGAQALQTVLDLEPALRKRD

9 FRQADIIKIAG**ND**GGAQALQAVIEHGPTLRQHG

10 FNLADIVKMAG**NI**GGAQALQAVLDLKPVLDEHG

11 FSQPDIVKMAG**NI**GGAQALQAVLSLGPALRERG

12 FSQPDIVKIAG**NT**GGAQALQAVLDLELTLVEHG

13 FSQPDIVRITG**NR**GGAQALQAVLALELTLRERG

14 FSQPDIVKIAG**NS**GGAQALQAVLDLELTFRERG

15 FSQADIVKIAG**ND**GGTQALHAVLDLERMLGERG

16 FSRADIVNVAG**NN**GGAQALKAVLEHEATLNERG

17 FSRADIVKIAG**NG**GGAQALKAVLEHEATLDERG

18 FSRADIVRIAG**NG**GGAQALKAVLEHGPTLNERG

19 FNLTDIVEMAA**NS**GGAQALKAVLEHGPTLRQRG

20 LSLIDIVEIAS**N**-GGAQALKAVLKYGPVLMQAG

+1 RSNEEIVHVAARRGGAGRIRKMVAP---LLERQ

**>**acBat1 ΔCTD

NND MSTAFVDQDKQMANRLN

-1 LSPLERSKIEKQYGGATTLAFISNKQNELAQI

0 LSRADILKIASYDCAAHALQAVLDCGPMLGKRG

1 FSQSDIVKIAG**NI**GGAQALQAVLDLESMLGKRG

2 FSRDDIAKMAG**NI**GGAQTLQAVLDLESAFRERG

3 FSQADIVKIAG**NN**GGAQALYSVLDVEPTLGKRG

4 FSRADIVKIAG**NT**GGAQALHTVLDLEPALGKRG

5 FSRIDIVKIAA**NN**GGAQALHAVLDLGPTLRECG

6 FSQATIAKIAG**NI**GGAQALQMVLDLGPALGKRG

7 FSQATIAKIAG**NI**GGAQALQTVLDLEPALCERG

8 FSQATIAKMAG**NN**GGAQALQTVLDLEPALRKRD

9 FRQADIIKIAG**ND**GGAQALQAVIEHGPTLRQHG

10 FNLADIVKMAG**NI**GGAQALQAVLDLKPVLDEHG

11 FSQPDIVKMAG**NI**GGAQALQAVLSLGPALRERG

12 FSQPDIVKIAG**NT**GGAQALQAVLDLELTLVEHG

13 FSQPDIVRITG**NR**GGAQALQAVLALELTLRERG

14 FSQPDIVKIAG**NS**GGAQALQAVLDLELTFRERG

15 FSQADIVKIAG**ND**GGTQALHAVLDLERMLGERG

16 FSRADIVNVAG**NN**GGAQALKAVLEHEATLNERG

17 FSRADIVKIAG**NG**GGAQALKAVLEHEATLDERG

18 FSRADIVRIAG**NG**GGAQALKAVLEHGPTLNERG

19 FNLTDIVEMAA**NS**GGAQALKAVLEHGPTLRQRG

20 LSLIDIVEIAS**N**-GGAQALKAVLKYGPVLMQAG

+1

**>**dBat RVD switch 1

NND MSTAFVDQDKQMANRLN

-1 LSPLERSKIEKQYGGATTLAFISNKQNELAQI

0 LSRADILKIASYDCAAHALQAVLDCGPMLGKRG

1 FSQSDIVKIAG**NI**GGAQALQAVLDLESMLGKRG

2 FSRDDIAKMAG**NN**GGAQTLQAVLDLESAFRERG

3 FSQADIVKIAG**NI**GGAQALYSVLDVEPTLGKRG

4 FSRADIVKIAG**NN**GGAQALHTVLDLEPALGKRG

5 FSRIDIVKIAA**NT**GGAQALHAVLDLGPTLRECG

6 FSQATIAKIAG**NI**GGAQALQMVLDLGPALGKRG

7 FSQATIAKIAG**NI**GGAQALQTVLDLEPALCERG

8 FSQATIAKMAG**NN**GGAQALQTVLDLEPALRKRD

9 FRQADIIKIAG**ND**GGAQALQAVIEHGPTLRQHG

10 FNLADIVKMAG**NI**GGAQALQAVLDLKPVLDEHG

11 FSQPDIVKMAG**NI**GGAQALQAVLSLGPALRERG

12 FSQPDIVKIAG**NT**GGAQALQAVLDLELTLVEHG

13 FSQPDIVRITG**NR**GGAQALQAVLALELTLRERG

14 FSQPDIVKIAG**NS**GGAQALQAVLDLELTFRERG

15 FSQADIVKIAG**ND**GGTQALHAVLDLERMLGERG

16 FSRADIVNVAG**NN**GGAQALKAVLEHEATLNERG

17 FSRADIVKIAG**NG**GGAQALKAVLEHEATLDERG

18 FSRADIVRIAG**NG**GGAQALKAVLEHGPTLNERG

19 FNLTDIVEMAA**NS**GGAQALKAVLEHGPTLRQRG

20 LSLIDIVEIAS**N**-GGAQALKAVLKYGPVLMQAG

+1 RSNEEIVHVAARRGGAGRIRKMVAP---LLERQ

**>**dBat RVD switch 2

NND MSTAFVDQDKQMANRLN

-1 LSPLERSKIEKQYGGATTLAFISNKQNELAQI

0 LSRADILKIASYDCAAHALQAVLDCGPMLGKRG

1 FSQSDIVKIAG**NI**GGAQALQAVLDLESMLGKRG

2 FSRDDIAKMAG**NI**GGAQTLQAVLDLESAFRERG

3 FSQADIVKIAG**NN**GGAQALYSVLDVEPTLGKRG

4 FSRADIVKIAG**NT**GGAQALHTVLDLEPALGKRG

5 FSRIDIVKIAA**NN**GGAQALHAVLDLGPTLRECG

6 FSQATIAKIAG**NI**GGAQALQMVLDLGPALGKRG

7 FSQATIAKIAG**NN**GGAQALQTVLDLEPALCERG

8 FSQATIAKMAG**ND**GGAQALQTVLDLEPALRKRD

9 FRQADIIKIAG**NI**GGAQALQAVIEHGPTLRQHG

10 FNLADIVKMAG**NI**GGAQALQAVLDLKPVLDEHG

11 FSQPDIVKMAG**NI**GGAQALQAVLSLGPALRERG

12 FSQPDIVKIAG**NT**GGAQALQAVLDLELTLVEHG

13 FSQPDIVRITG**NR**GGAQALQAVLALELTLRERG

14 FSQPDIVKIAG**NS**GGAQALQAVLDLELTFRERG

15 FSQADIVKIAG**ND**GGTQALHAVLDLERMLGERG

16 FSRADIVNVAG**NN**GGAQALKAVLEHEATLNERG

17 FSRADIVKIAG**NG**GGAQALKAVLEHEATLDERG

18 FSRADIVRIAG**NG**GGAQALKAVLEHGPTLNERG

19 FNLTDIVEMAA**NS**GGAQALKAVLEHGPTLRQRG

20 LSLIDIVEIAS**N**-GGAQALKAVLKYGPVLMQAG

+1 RSNEEIVHVAARRGGAGRIRKMVAP---LLERQ

**>**dBat RVD switch 3

NNDMSTAFVDQDKQMANRLN

-1 LSPLERSKIEKQYGGATTLAFISNKQNELAQI

0 LSRADILKIASYDCAAHALQAVLDCGPMLGKRG

1 FSQSDIVKIAG**NI**GGAQALQAVLDLESMLGKRG

2 FSRDDIAKMAG**NI**GGAQTLQAVLDLESAFRERG

3 FSQADIVKIAG**NN**GGAQALYSVLDVEPTLGKRG

4 FSRADIVKIAG**NT**GGAQALHTVLDLEPALGKRG

5 FSRIDIVKIAA**NN**GGAQALHAVLDLGPTLRECG

6 FSQATIAKIAG**NI**GGAQALQMVLDLGPALGKRG

7 FSQATIAKIAG**NI**GGAQALQTVLDLEPALCERG

8 FSQATIAKMAG**NN**GGAQALQTVLDLEPALRKRD

9 FRQADIIKIAG**ND**GGAQALQAVIEHGPTLRQHG

10 FNLADIVKMAG**NI**GGAQALQAVLDLKPVLDEHG

11 FSQPDIVKMAG**NT**GGAQALQAVLSLGPALRERG

12 FSQPDIVKIAG**NI**GGAQALQAVLDLELTLVEHG

13 FSQPDIVRITG**NR**GGAQALQAVLALELTLRERG

14 FSQPDIVKIAG**NS**GGAQALQAVLDLELTFRERG

15 FSQADIVKIAG**ND**GGTQALHAVLDLERMLGERG

16 FSRADIVNVAG**NN**GGAQALKAVLEHEATLNERG

17 FSRADIVKIAG**NG**GGAQALKAVLEHEATLDERG

18 FSRADIVRIAG**NG**GGAQALKAVLEHGPTLNERG

19 FNLTDIVEMAA**NS**GGAQALKAVLEHGPTLRQRG

20 LSLIDIVEIAS**N**-GGAQALKAVLKYGPVLMQAG

+1 RSNEEIVHVAARRGGAGRIRKMVAP---LLERQ

**>**dBat RVD switch 4

NNDMSTAFVDQDKQMANRLN

-1 LSPLERSKIEKQYGGATTLAFISNKQNELAQI

0 LSRADILKIASYDCAAHALQAVLDCGPMLGKRG

1 FSQSDIVKIAG**NI**GGAQALQAVLDLESMLGKRG

2 FSRDDIAKMAG**NI**GGAQTLQAVLDLESAFRERG

3 FSQADIVKIAG**NN**GGAQALYSVLDVEPTLGKRG

4 FSRADIVKIAG**NT**GGAQALHTVLDLEPALGKRG

5 FSRIDIVKIAA**NN**GGAQALHAVLDLGPTLRECG

6 FSQATIAKIAG**NI**GGAQALQMVLDLGPALGKRG

7 FSQATIAKIAG**NI**GGAQALQTVLDLEPALCERG

8 FSQATIAKMAG**NN**GGAQALQTVLDLEPALRKRD

9 FRQADIIKIAG**ND**GGAQALQAVIEHGPTLRQHG

10 FNLADIVKMAG**NI**GGAQALQAVLDLKPVLDEHG

11 FSQPDIVKMAG**NI**GGAQALQAVLSLGPALRERG

12 FSQPDIVKIAG**NT**GGAQALQAVLDLELTLVEHG

13 FSQPDIVRITG**NR**GGAQALQAVLALELTLRERG

14 FSQPDIVKIAG**NS**GGAQALQAVLDLELTFRERG

15 FSQADIVKIAG**ND**GGTQALHAVLDLERMLGERG

16 FSRADIVNVAG**NN**GGAQALKAVLEHEATLNERG

17 FSRADIVKIAG**NG**GGAQALKAVLEHEATLDERG

18 FSRADIVRIAG**N-**GGAQALKAVLEHGPTLNERG

19 FNLTDIVEMAA**NS**GGAQALKAVLEHGPTLRQRG

20 LSLIDIVEIAS**NG**GGAQALKAVLKYGPVLMQAG

+1 RSNEEIVHVAARRGGAGRIRKMVAP---LLERQ

>dBat Repeat switch 1

NND MSTAFVDQDKQMANRLN

-1 LSPLERSKIEKQYGGATTLAFISNKQNELAQI

0 LSRADILKIASYDCAAHALQAVLDCGPMLGKRG

2 FSRDDIAKMAG**NI**GGAQTLQAVLDLESAFRERG

1 FSQSDIVKIAG**NI**GGAQALQAVLDLESMLGKRG

4 FSRADIVKIAG**NT**GGAQALHTVLDLEPALGKRG

5 FSRIDIVKIAA**NN**GGAQALHAVLDLGPTLRECG

3 FSQADIVKIAG**NN**GGAQALYSVLDVEPTLGKRG

6 FSQATIAKIAG**NI**GGAQALQMVLDLGPALGKRG

7 FSQATIAKIAG**NI**GGAQALQTVLDLEPALCERG

8 FSQATIAKMAG**NN**GGAQALQTVLDLEPALRKRD

9 FRQADIIKIAG**ND**GGAQALQAVIEHGPTLRQHG

10 FNLADIVKMAG**NI**GGAQALQAVLDLKPVLDEHG

11 FSQPDIVKMAG**NI**GGAQALQAVLSLGPALRERG

12 FSQPDIVKIAG**NT**GGAQALQAVLDLELTLVEHG

13 FSQPDIVRITG**NR**GGAQALQAVLALELTLRERG

14 FSQPDIVKIAG**NS**GGAQALQAVLDLELTFRERG

15 FSQADIVKIAG**ND**GGTQALHAVLDLERMLGERG

16 FSRADIVNVAG**NN**GGAQALKAVLEHEATLNERG

17 FSRADIVKIAG**NG**GGAQALKAVLEHEATLDERG

18 FSRADIVRIAG**NG**GGAQALKAVLEHGPTLNERG

19 FNLTDIVEMAA**NS**GGAQALKAVLEHGPTLRQRG

20 LSLIDIVEIAS**N**-GGAQALKAVLKYGPVLMQAG

+1 RSNEEIVHVAARRGGAGRIRKMVAP---LLERQ

>dBat Repeat switch 2

NND MSTAFVDQDKQMANRLN

-1 LSPLERSKIEKQYGGATTLAFISNKQNELAQI

0 LSRADILKIASYDCAAHALQAVLDCGPMLGKRG

1 FSQSDIVKIAG**NI**GGAQALQAVLDLESMLGKRG

2 FSRDDIAKMAG**NI**GGAQTLQAVLDLESAFRERG

3 FSQADIVKIAG**NN**GGAQALYSVLDVEPTLGKRG

4 FSRADIVKIAG**NT**GGAQALHTVLDLEPALGKRG

5 FSRIDIVKIAA**NN**GGAQALHAVLDLGPTLRECG

7 FSQATIAKIAG**NI**GGAQALQTVLDLEPALCERG

10 FNLADIVKMAG**NI**GGAQALQAVLDLKPVLDEHG

8 FSQATIAKMAG**NN**GGAQALQTVLDLEPALRKRD

9 FRQADIIKIAG**ND**GGAQALQAVIEHGPTLRQHG

6 FSQATIAKIAG**NI**GGAQALQMVLDLGPALGKRG

11 FSQPDIVKMAG**NI**GGAQALQAVLSLGPALRERG

12 FSQPDIVKIAG**NT**GGAQALQAVLDLELTLVEHG

13 FSQPDIVRITG**NR**GGAQALQAVLALELTLRERG

14 FSQPDIVKIAG**NS**GGAQALQAVLDLELTFRERG

15 FSQADIVKIAG**ND**GGTQALHAVLDLERMLGERG

16 FSRADIVNVAG**NN**GGAQALKAVLEHEATLNERG

17 FSRADIVKIAG**NG**GGAQALKAVLEHEATLDERG

18 FSRADIVRIAG**NG**GGAQALKAVLEHGPTLNERG

19 FNLTDIVEMAA**NS**GGAQALKAVLEHGPTLRQRG

20 LSLIDIVEIAS**N**-GGAQALKAVLKYGPVLMQAG

+1 RSNEEIVHVAARRGGAGRIRKMVAP---LLERQ

>dBat Repeat switch 3

NND MSTAFVDQDKQMANRLN

-1 LSPLERSKIEKQYGGATTLAFISNKQNELAQI

0 LSRADILKIASYDCAAHALQAVLDCGPMLGKRG

1 FSQSDIVKIAG**NI**GGAQALQAVLDLESMLGKRG

2 FSRDDIAKMAG**NI**GGAQTLQAVLDLESAFRERG

3 FSQADIVKIAG**NN**GGAQALYSVLDVEPTLGKRG

4 FSRADIVKIAG**NT**GGAQALHTVLDLEPALGKRG

5 FSRIDIVKIAA**NN**GGAQALHAVLDLGPTLRECG

6 FSQATIAKIAG**NI**GGAQALQMVLDLGPALGKRG

7 FSQATIAKIAG**NI**GGAQALQTVLDLEPALCERG

8 FSQATIAKMAG**NN**GGAQALQTVLDLEPALRKRD

9 FRQADIIKIAG**ND**GGAQALQAVIEHGPTLRQHG

10 FNLADIVKMAG**NI**GGAQALQAVLDLKPVLDEHG

12 FSQPDIVKIAG**NT**GGAQALQAVLDLELTLVEHG

11 FSQPDIVKMAG**NI**GGAQALQAVLSLGPALRERG

13 FSQPDIVRITG**NR**GGAQALQAVLALELTLRERG

14 FSQPDIVKIAG**NS**GGAQALQAVLDLELTFRERG

15 FSQADIVKIAG**ND**GGTQALHAVLDLERMLGERG

16 FSRADIVNVAG**NN**GGAQALKAVLEHEATLNERG

17 FSRADIVKIAG**NG**GGAQALKAVLEHEATLDERG

18 FSRADIVRIAG**NG**GGAQALKAVLEHGPTLNERG

19 FNLTDIVEMAA**NS**GGAQALKAVLEHGPTLRQRG

20 LSLIDIVEIAS**N**-GGAQALKAVLKYGPVLMQAG

+1 RSNEEIVHVAARRGGAGRIRKMVAP---LLERQ

>dBat Repeat switch 4

NND MSTAFVDQDKQMANRLN

-1 LSPLERSKIEKQYGGATTLAFISNKQNELAQI

0 LSRADILKIASYDCAAHALQAVLDCGPMLGKRG

1 FSQSDIVKIAG**NI**GGAQALQAVLDLESMLGKRG

2 FSRDDIAKMAG**NI**GGAQTLQAVLDLESAFRERG

3 FSQADIVKIAG**NN**GGAQALYSVLDVEPTLGKRG

4 FSRADIVKIAG**NT**GGAQALHTVLDLEPALGKRG

5 FSRIDIVKIAA**NN**GGAQALHAVLDLGPTLRECG

6 FSQATIAKIAG**NI**GGAQALQMVLDLGPALGKRG

7 FSQATIAKIAG**NI**GGAQALQTVLDLEPALCERG

8 FSQATIAKMAG**NN**GGAQALQTVLDLEPALRKRD

9 FRQADIIKIAG**ND**GGAQALQAVIEHGPTLRQHG

10 FNLADIVKMAG**NI**GGAQALQAVLDLKPVLDEHG

11 FSQPDIVKMAG**NI**GGAQALQAVLSLGPALRERG

12 FSQPDIVKIAG**NT**GGAQALQAVLDLELTLVEHG

13 FSQPDIVRITG**NR**GGAQALQAVLALELTLRERG

14 FSQPDIVKIAG**NS**GGAQALQAVLDLELTFRERG

15 FSQADIVKIAG**ND**GGTQALHAVLDLERMLGERG

16 FSRADIVNVAG**NN**GGAQALKAVLEHEATLNERG

17 FSRADIVKIAG**NG**GGAQALKAVLEHEATLDERG

20 LSLIDIVEIAS**N**-GGAQALKAVLKYGPVLMQAG

19 FNLTDIVEMAA**NS**GGAQALKAVLEHGPTLRQRG

18 FSRADIVRIAG**NG**GGAQALKAVLEHGPTLNERG

+1 RSNEEIVHVAARRGGAGRIRKMVAP---LLERQ

**Supplementary Figure 11:** Nucleotide and amino acid sequences of dBat_SOX2_-RVD switch and -repeat switch.

Genes encoding the dBats were synthesised with *E. coli* codon usage (GenScript). One block encodes the N- and C-terminal regions including the cryptic repeats, separated by BpiI sites, flanked by BsaI sites. This was assembled via BsaI cut-ligation into the pVAX destination vector. Repeats were encoded on two BpiI flanked modules assembled directly into the destination vector via BpiI cut-ligation. BsaI recognition sites are underlined and BpiI sites grey-highlighted, while bold typeface marks the overlaps created upon digest.

In the amino acid sequences consecutive repeats are numbered, corresponding to the repeats of wild type Bat1. The RVDs (residues at repeat positions 12 and 13) are marked as boldface black letters on grey background. The sections encoded by the BsaI-flanked N- and C- terminal module are underlined.

>Bat1 N-BpiI BpiI-C

GGTCTCT**TATG**AGCACCGCCTTCGTGGACCAAGATAAGCAAATGGCAAACCGCCTGAACCTGTCACCGCTGGAACGTAGCAAAATTGAAAAACAATATGGCGGTGCAACCACGCTGGCTTTTATTAGCAACAAACAGAATGAACTGGCACAAATCCTGAGCCGTGCTGATATTCTGAAAATCGCGTCTTACGACTGCGCAGCACATGCACTGCAGGCTGTCCTGGATTGTGGCCCGATGCTGGGCAAACGCGGTTT**TAGC**TAGTCTTCTAGAAGACTA**GGCG**GTGCGCAGGCCCTGAAAGCTGTCCTGAAGTATGGTCCGGTGCTGATGCAAGCAGGTCGTAGCAATGAAGAAATCGTGCACGTTGCCGCTCGTCGTGGTGGTGCTGGCCGTATCCGTAAGATGGTTGCTCCGCTGCTGGAACGTCAG**GGTG**TGAGACC

>dBat_SOX2_ Repeat switch AB

GAAGACTT**TAGC**CGCGCAGATATTGTCAAGATCGCGGGTAACGGTGGCGGCGCACAAGCACTGAAGGCGGTTCTGGAACACGAAGCGACCCTGGATGAAAGCGGCTTTAGTCGCGCAGATATTGTCAAGATCGCGGGTAACGGTGGCGGCGCACAAGCACTGAAGGCGGTTCTGGAACACGAAGCGACCCTGGATGAAAGCGGCTTCTCCCGCGATGACATTGCGAAGATGGCCGGCAATATCGGCGGTGCACAGACCCTGCAGGCCGTGCTGGATCTGGAATCAGCCTTTCGTGAACGCGGCTTTTCTCGTGCTGATATTGTCCGTATTGCGGGTAATGGTGGTGGTGCCCAGGCTCTGAAGGCTGTGCTGGAACATGGTCCGACGCTGAACGAACGTGGCTTTTCTCGTGCTGATATTGTCCGTATTGCGGGTAATGGTGGTGGTGCCCAGGCTCTGAAGGCTGTGCTGGAACATGGTCCGACGCTGAACGAACGTGGCTTTCGTCAGGCGGACATTATCAAGATTGCCGGTAATGACGGTGGCGCCCAGGCACTGCAAGCAGTGATCGAACATGGCCCGACCCTGCGCCAACACGGTTTTAGCCAGGCGGATATTGTCAAAATCGCCGGTAACGACGGCGGTACCCAAGCACTGCATGCTGTGCTGGATCTGGAACGTATGCTGGGCGAACGTGGTTTTCGTCAGGCGGACATTATCAAGATTGCCGGTAATGACGGTGGCGCCCAGGCACTGCAAGCAGTGATCGAACATGGCCCGACCCTGCGCCAACACGGTTTTAGTCGCGCAGATATTGTCAAGATCGCGGGTAACGGTGGCGGCGCACAAGCACTGAAGGCGGTTCTGGAACACGAAGCGACCCTGGATGAAAGCG**GTTT**TAGTCTTC

>dBat_SOX2_ Repeat switch BC

GAAGACTG**GTTT**CTCCCGCATTGATATCGTTAAGATCGCAGCTAACAACGGTGGTGCTCAAGCCCTGCACGCTGTCCTGGATCTGGGTCCGACGCTGCGCGAATGTGGGTTCTCGCAGGCAACCATCGCAAAAATCGCTGGCAATATCGGCGGTGCTCAGGCTCTGCAAATGGTGCTGGATCTGGGTCCGGCTCTGGGCAAACGTGGTTTTAGCCAGGCGGATATTGTCAAAATCGCCGGTAACGACGGCGGTACCCAAGCACTGCATGCTGTGCTGGATCTGGAACGTATGCTGGGCGAACGTGGTTTTAGCCAGTCTGACATTGTCAAGATCGCCGGTAACATTGGCGGTGCACAGGCACTGCAAGCAGTGCTGGATCTGGAAAGTATGCTGGGCAAACGTGGTTTCTCGCAGGCCGACATTGTTAAAATCGCCGGTAACAATGGCGGTGCACAAGCTCTGTATAGTGTGCTGGATGTTGAACCGACCCTGGGTAAACGTGGTTTTCGTCAGGCGGACATTATCAAGATTGCCGGTAATGACGGTGGCGCCCAGGCACTGCAAGCAGTGATCGAACATGGCCCGACCCTGCGCCAACACGGTTTTAGCCAGGCGGATATTGTCAAAATCGCCGGTAACGACGGCGGTACCCAAGCACTGCATGCTGTGCTGGATCTGGAACGTATGCTGGGCGAACGTGGTTTTCGTCAGGCGGACATTATCAAGATTGCCGGTAATGACGGTGGCGCCCAGGCACTGCAAGCAGTGATCGAACATGGCCCGACCCTGCGCCAACACGGTTTTAGCCAGGCGGATATTGTCAAAATCGCCGGTAACGAC**GGCG**AAGTCTTC

>dBat_SOX2_ RVD switch AB

GAAGACTT**TAGC**CAGTCTGACATTGTCAAGATCGCCGGTAACGGTGGCGGTGCACAGGCACTGCAAGCAGTGCTGGATCTGGAAAGTATGCTGGGCAAACGTGGTTTCTCCCGCGATGACATTGCGAAGATGGCCGGCAATGGTGGCGGTGCACAGACCCTGCAGGCCGTGCTGGATCTGGAATCAGCCTTTCGTGAACGCGGCTTCTCGCAGGCCGACATTGTTAAAATCGCCGGTAACATTGGCGGTGCACAAGCTCTGTATAGTGTGCTGGATGTTGAACCGACCCTGGGTAAACGTGGTTTTTCACGCGCTGACATTGTTAAGATCGCCGGTAACGGTGGCGGTGCCCAAGCACTGCACACGGTCCTGGATCTGGAACCGGCCCTGGGCAAGCGTGGTTTCTCCCGCATTGATATCGTTAAGATCGCAGCTAACGGTGGTGGTGCTCAAGCCCTGCACGCTGTCCTGGATCTGGGTCCGACGCTGCGCGAATGTGGGTTCTCGCAGGCAACCATCGCAAAAATCGCTGGCAATGATGGCGGTGCTCAGGCTCTGCAAATGGTGCTGGATCTGGGTCCGGCTCTGGGCAAACGTGGTTTTAGCCAGGCAACCATTGCTAAGATCGCCGGTAACGATGGCGGTGCACAGGCACTGCAAACGGTCCTGGATCTGGAACCGGCGCTGTGCGAACGCGGCTTCTCTCAGGCCACCATCGCAAAAATGGCTGGTAACGATGGCGGTGCACAGGCTCTGCAAACGGTTCTGGATCTGGAACCGGCCCTGCGTAAACGCGATTTTCGTCAGGCGGACATTATCAAGATTGCCGGTAATGGTGGTGGCGCCCAGGCACTGCAAGCAGTGATCGAACATGGCCCGACCCTGCGCCAACACG**GTTT**TAGTCTTC

>dBat_SOX2_ RVD switch BC

GAAGACTA**GTTT**CAACCTGGCAGACATTGTTAAGATGGCTGGTAATAATGGTGGTGCTCAAGCTCTGCAAGCGGTGCTGGACCTGAAGCCGGTGCTGGACGAACATGGTTTCTCTCAACCGGATATCGTCAAGATGGCGGGCAACATTGGTGGTGCTCAAGCCCTGCAAGCCGTCCTGTCACTGGGTCCGGCGCTGCGTGAACGTGGCTTTAGCCAGCCGGATATTGTCAAAATCGCCGGTAACGACGGCGGTGCACAGGCACTGCAAGCAGTGCTGGATCTGGAACTGACGCTGGTTGAACATGGCTTCTCTCAACCGGACATTGTTCGCATCACCGGTAATATTGGCGGTGCCCAAGCTCTGCAAGCGGTGCTGGCTCTGGAACTGACCCTGCGTGAACGAGGATTTAGCCAACCGGACATCGTGAAAATCGCGGGCAATAACGGCGGTGCTCAAGCTCTGCAAGCGGTCCTGGATCTGGAACTGACGTTTCGTGAACGCGGCTTTAGCCAGGCGGATATTGTCAAAATCGCCGGTAACGACGGCGGTACCCAAGCACTGCATGCTGTGCTGGATCTGGAACGTATGCTGGGCGAACGTGGTTTCTCTCGCGCAGACATTGTGAACGTTGCTGACAACAATGGCGGTGCGCAGGCCCTGAAAGCCGTGCTGGAACACGAAGCCACGCTGAATGAACGTGGCTTTAGTCGCGCAGATATTGTCAAGATCGCGGGTAACGATGGCGGCGCACAAGCACTGAAGGCGGTTCTGGAACACGAAGCGACCCTGGATGAAAGCGGCTTTTCTCGTGCTGATATTGTCCGTATTGCGGGTAATGAT**GGCG**AAGTCTTC

>dBat_SOX2_ RVD switch

NND MSTAFVDQDKQMANRLN

-1 LSPLERSKIEKQYGGATTLAFISNKQNELAQI

0 LSRADILKIASYDCAAHALQAVLDCGPMLGKRG

1 FSQSDIVKIAG**NG**GGAQALQAVLDLESMLGKRG

2 FSRDDIAKMAG**NG**GGAQTLQAVLDLESAFRERG

3 FSQADIVKIAG**NI**GGAQALYSVLDVEPTLGKRG

4 FSRADIVKIAG**NG**GGAQALHTVLDLEPALGKRG

5 FSRIDIVKIAA**NG**GGAQALHAVLDLGPTLRECG

6 FSQATIAKIAG**ND**GGAQALQMVLDLGPALGKRG

7 FSQATIAKIAG**ND**GGAQALQTVLDLEPALCERG

8 FSQATIAKMAG**ND**GGAQALQTVLDLEPALRKRD

9 FRQADIIKIAG**NG**GGAQALQAVIEHGPTLRQHG

10 FNLADIVKMAG**NN**GGAQALQAVLDLKPVLDEHG

11 FSQPDIVKMAG**NI**GGAQALQAVLSLGPALRERG

12 FSQPDIVKIAG**ND**GGAQALQAVLDLELTLVEHG

13 FSQPDIVRITG**NI**GGAQALQAVLALELTLRERG

14 FSQPDIVKIAG**NN**GGAQALQAVLDLELTFRERG

15 FSQADIVKIAG**ND**GGTQALHAVLDLERMLGERG

16 FSRADIVNVAD**NN**GGAQALKAVLEHEATLNERG

17 FSRADIVKIAG**ND**GGAQALKAVLEHEATLDESG

18 FSRADIVRIAG**ND**GGAQALKAVLKYGPVLMQAG

+1 RSNEEIVHVAARRGGAGRIRKMVAP---LLERQ

>dBat_SOX2_ repeat switch

NND MSTAFVDQDKQMANRLN

-1 LSPLERSKIEKQYGGATTLAFISNKQNELAQI

0 LSRADILKIASYDCAAHALQAVLDCGPMLGKRG

17 FSRADIVKIAG**NG**GGAQALKAVLEHEATLDERG

17 FSRADIVKIAG**NG**GGAQALKAVLEHEATLDERG

2 FSRDDIAKMAG**NI**GGAQTLQAVLDLESAFRERG

18 FSRADIVRIAG**NG**GGAQALKAVLEHGPTLNERG

18 FSRADIVRIAG**NG**GGAQALKAVLEHGPTLNERG

9 FRQADIIKIAG**ND**GGAQALQAVIEHGPTLRQHG

15 FSQADIVKIAG**ND**GGTQALHAVLDLERMLGERG

9 FRQADIIKIAG**ND**GGAQALQAVIEHGPTLRQHG

17 FSRADIVKIAG**NG**GGAQALKAVLEHEATLDERG

5 FSRIDIVKIAA**NN**GGAQALHAVLDLGPTLRECG

6 FSQATIAKIAG**NI**GGAQALQMVLDLGPALGKRG

15 FSQADIVKIAG**ND**GGTQALHAVLDLERMLGERG

1 FSQSDIVKIAG**NI**GGAQALQAVLDLESMLGKRG

3 FSQADIVKIAG**NN**GGAQALYSVLDVEPTLGKRG

9 FRQADIIKIAG**ND**GGAQALQAVIEHGPTLRQHG

15 FSQADIVKIAG**ND**GGTQALHAVLDLERMLGERG

9 FRQADIIKIAG**ND**GGAQALQAVIEHGPTLRQHG

15 FSQADIVKIAG**ND**GGAQALKAVLKYGPVLMQAG

+1 RSNEEIVHVAARRGGAGRIRKMVAP---LLERQ

**Supplementary figure 12:** specificity test with the BE*_pSOX2_* targeted dBats.

Both dBats were tested against the BE_Bat1_ reporter as described in Materials and Methods. The number of cells analysed is indicated below each pseudodensity plot and the vertical bar indicates the threshold Alexa Fluor 594 level above which cells were considered as expressing the relevant Bat or TALE construct and included in downstream analysis. Colour from blue-green to yellow-red indicates increasing cell density. The box plots show fold-change in dsEGFP fluorescence intensity relative to the reporter only control for the two dBats against either the BE*_pSOX2_* or BE_Bat1_ reporters. Median values are given next to the boxes in each case.

**
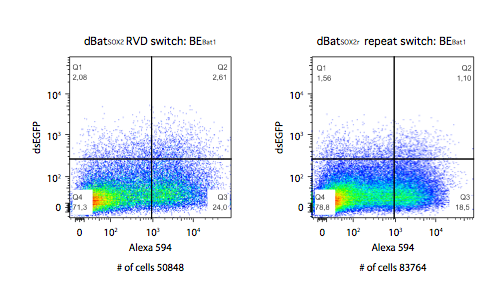
**

**Supplementary Figure 13:** Pseudocolour density blots of fluorescence and extended boxplots including outliers for experiments shown in Figures 3, 5-7.

dsEGFP and Alexa Fluor 594 fluorescence levels are shown for all cells analysed for the preparation of figures 3 and 5-7. Data are sorted by figure and transfected constructs are written above the plot in each case. The number of cells analysed is indicated below. The vertical bar indicates the threshold Alexa Fluor 594 level above which cells were considered as expressing the relevant Bat or TALE construct and included in downstream analysis. The x-axis utilises a logical display. Colour from blue-green to yellow-red indicates increasing cell density. Boxplots are also sorted by figure and transfected constructs are given beside each plot. dsEGFP fluorescence is given relative to the reporter alone and is shown only for those cells with above-threshold Alexa Fluor 594 levels.


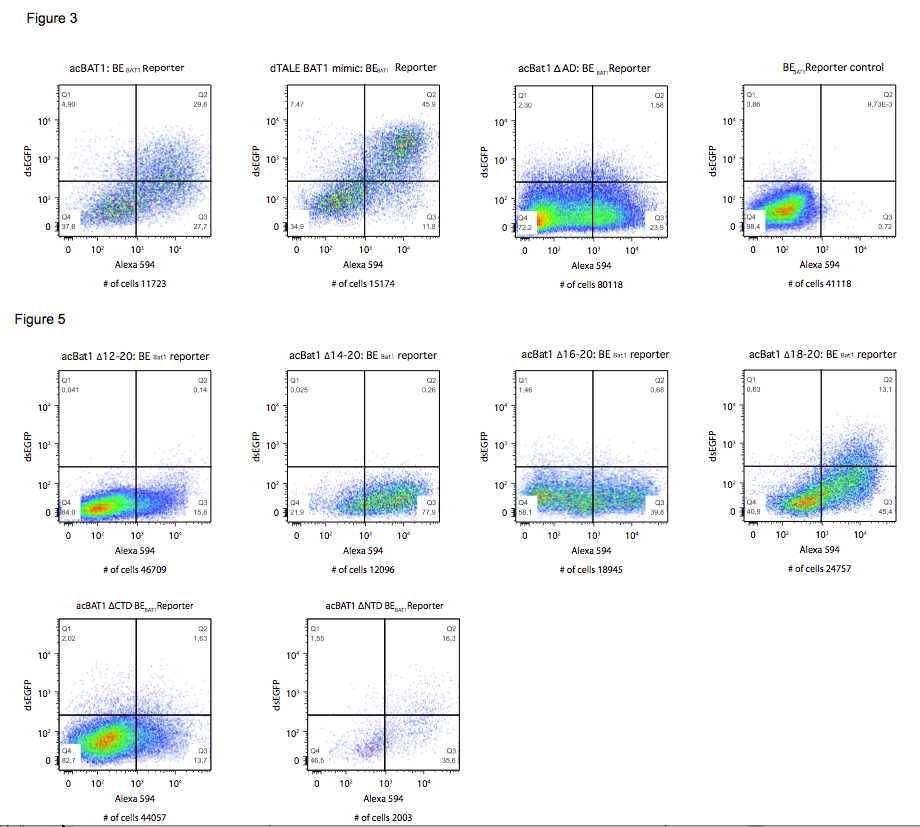


Figure 6


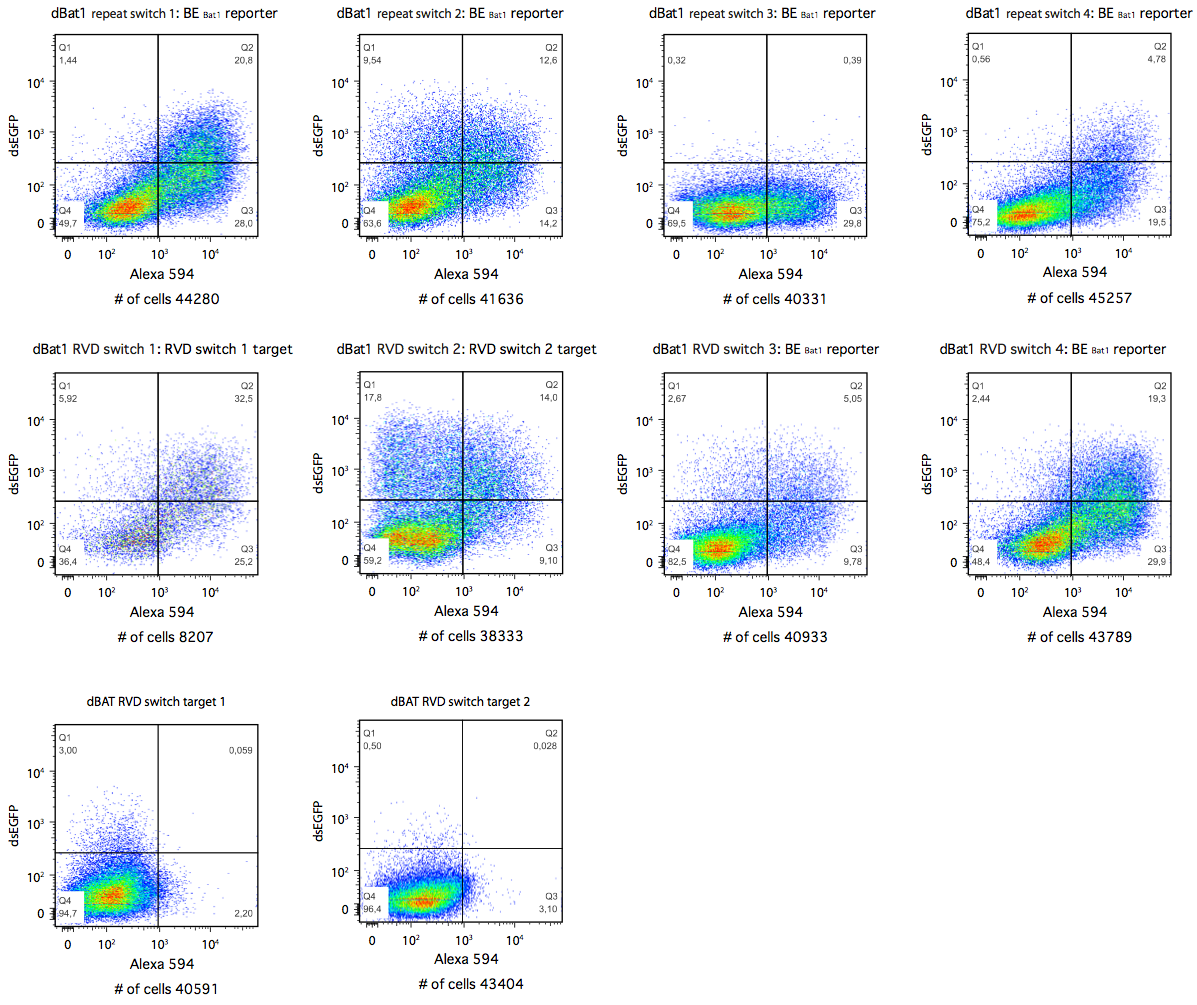


**
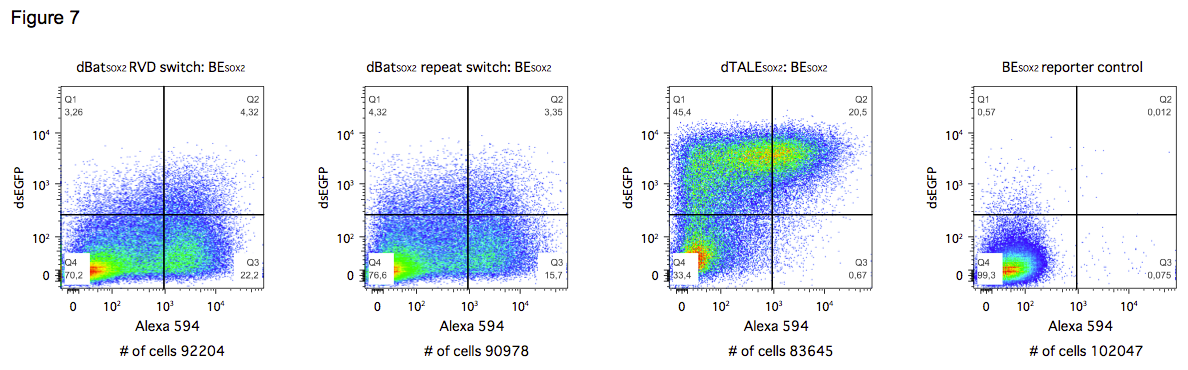
**

**
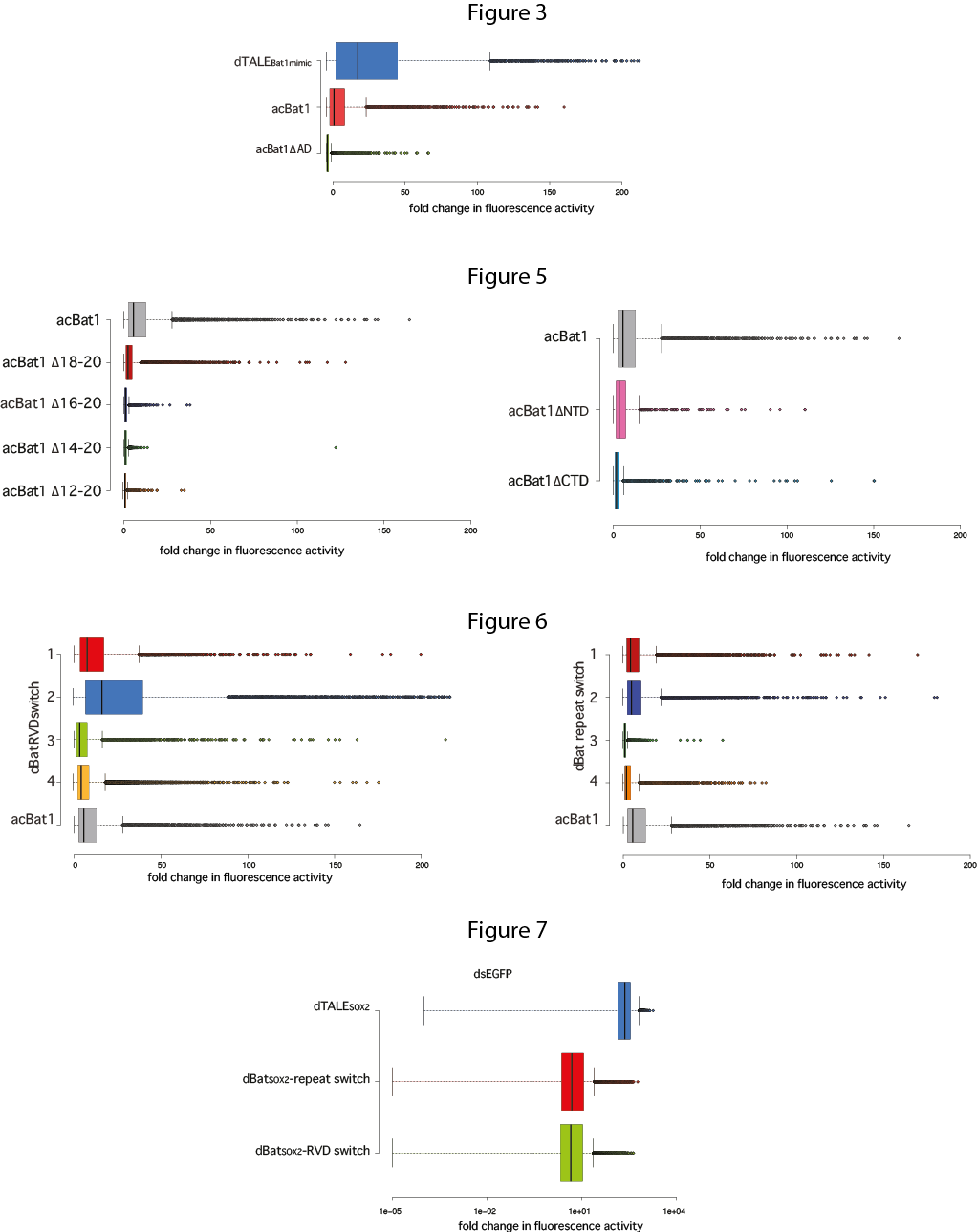
**

**Supplementary Figure 14:** Amino acid sequences of the Bat1 repeat trimers used in Figure 8. The sequences corresponding to the Bat repeats are shown in bold and the central repeat of the trimer is underlined to allow each repeat to be identified. Flanking sequences correspond to sections of AvrBs3 necessary for cloning via the previously established toolkit (15). Sequences corresponding to the terminal BpiI recognition sites facilitating compatibility with the TALE binding domain assembly toolkit are highlighted and are removed during cloning.

**>Bat1 repeat 2 trimer**

EDAETVQRLLPVLCQAHG**FSRDDIAKMAGNIGGAQTLQAVLDLESAFRERGFSRDDIAKMAGNIGGAQTLQAVLDLESAFRERGFSRDDIAKMAGNIGGAQTLQAVLDLESAFRERG** LTPEQVVAIASQS

**>Bat1 repeat 6 trimer**

EDAETVQRLLPVLCQAHG**FSQATIAKIAGNIGGAQALQMVLDLGPALGKRGFSQATIAKIAGNIGGAQALQMVLDLGPALGKRGFSQATIAKIAGNIGGAQALQMVLDLGPALGKRG** LTPEQVVAIASQS

**>Bat1 repeat 8 trimer**

EDAETVQRLLPVLCQAHG**FSQATIAKMAGNNGGAQALQTVLDLEPALRKRDFSQATIAKMAGNNGGAQALQTVLDLEPALRKRDFSQATIAKMAGNNGGAQALQTVLDLEPALRKRD** LTPEQVVAIASQS

**>Bat1 repeat 17 trimer**

EDAETVQRLLPVLCQAHG**FSRADIVKIAGNGGGAQALKAVLEHEATLDERGFSRADIVKIAGNGGGAQALKAVLEHEATLDERGFSRADIVKIAGNGGGAQALKAVLEHEATLDERG** LTPEQVVAIASQS

**Supplementary Figure 15:** Structural predictions for Bat1 based on the structure of PthXo1 bound to DNA.

Homology Model: Created using SWISS-MODEL (38)

Template: 3UGM PthXo1

Sequence identity 38.20%

Range of Bat1 covered by the alignment: 11-767

GMQE: 0.70,

QMEANA4: -6.75,

Diameter of pore: 16.5-19 Angstroms

Average inter-repeat angle: 33°


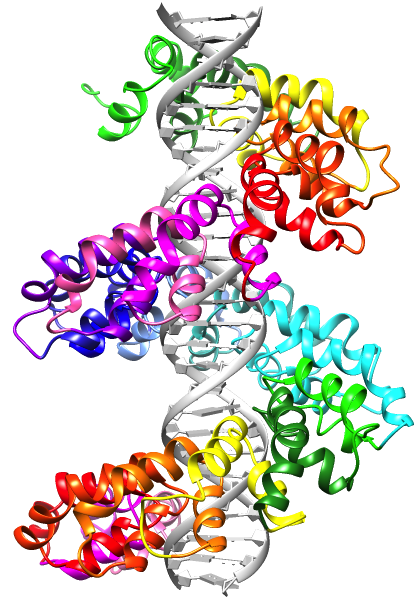


Model of Bat1 wrapped around BE_Bat1_ (silver) based on the structure of PthXo1 bound to its target DNA shown from N- to C-terminus going down the page. Each repeat is coloured individually.


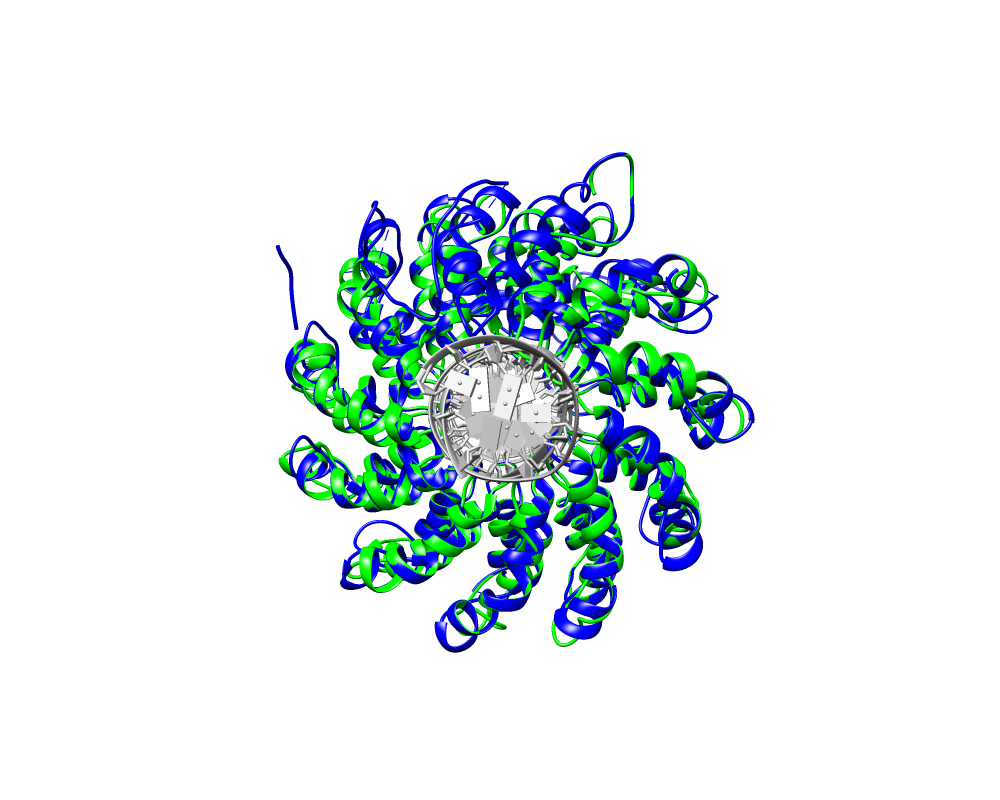


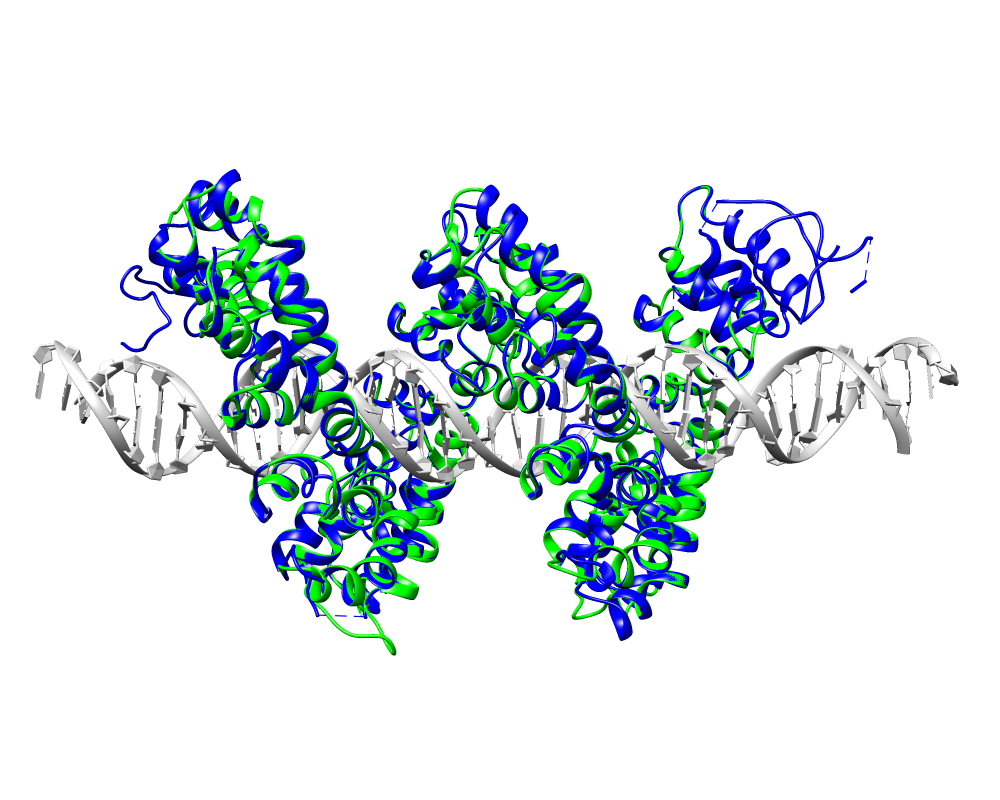


Longitudinal and transverse views of the Bat1 structural prediction (green) aligned to the structure of PthXo1 (blue). PthXo1 target DNA is shown (silver). Created in UCSF Chimera (39).

| **Table S1: Percentage sequence identities of the Bat proteins sorted by domain.** | | | | |
| --- | --- | --- | --- | --- |
|  | NND | Repeats  -1/0 | Consensus core repeats | Repeat +1 |
| Bat1 ⇔ Bat2 | 50 | 86 | 94 | 97 |
| Bat1 ⇔ Bat3 | 39 | 66 | 73 | 67 |
| Bat2 ⇔ Bat3 | 50 | 66 | 76 | 67 |
| Consensus core refers to the consensus formed from an alignment of all the core repeats of a single Bat protein. Alignments were performed on CLC Main Workbench 6.1. (Gap open cost 10.0, Gap extension cost 1.0). Percentage identities shown to two significant figures. | | | | |

| **Table S2: A list of primers used in this study** | | |
| --- | --- | --- |
| **Primer name** | **Sequence** | **Notes** |
| pUC57 BB D Fwd | GGG GTC TCT TAA CTA GTC TTC GGG CCC GTC GAC TG | Used to create modified *TALE* toolkit level 2 vector pUC57-CD-DEST. 5’ phosphorylated |
| pUC57 BB C Rev | CCT TGG TCT CAG GGT TAG TCT TCC GAT ATC TAG ATG C | Used to create modified *TALE* toolkit level 2 vector pUC57-CD-DEST |
| Toolkit_N12_Rev | ATT GCT GGC GAT GGC CAC CAC C | 5’ Phosphorylated. Used to modify RVDs of *TALE* toolkit repeats |
| Rep7C_13T_Fwd | ACC GGT GGC AAG CAG GCG CTG | Used to create modified *TALE* toolkit repeat 7C_NT |
| Rep4_13T_Fwd | ACC GGC AAG CAG GCG CTT GAG | Used to create modified *TALE* toolkit repeat 4_NT |
| Rep3_ 13D_Fwd | GAC GGT GGC AAG CAG GCG CTG | Used to create modified *TALE* toolkit repeat 3_ND |
| Rep4_13D_Fwd | GAC GGC AAG CAG GCG CTT GAG | Used to create modified *TALE* toolkit repeat 4_ND |
| Toolkit_13R_Fwd | CGG GGT GGC AAG CAG GCG CTG | Used to create modified *TALE* toolkit repeat 1C_NR |
| 1/2_13*_Fwd | GGC GGC AGG CCG GCG C | Used to create modified *TALE* toolkit repeat D1/2_N* |
| rep6_mut_6 ½ Fwd | CGA GAG ACC CCG GGA TCC GAT ATC TAG | Used to create B overlap on toolkit repeat 6 (6 ½ B) |
| rep_mut_rep ½ Rev | CTA CCA CCT GCT CCG GGG TCA GGC | Used to create B overlap on toolkit repeat 6 (6 ½ B). 5’ phosphorylated. |
| Linker 5-6 ½ Fwd | CGG GTC TCT TGA GGG GGA GCG TGA GAC CTG | Used to create Linker 5-6 in pUC57 with two BsaI sites. Repeats 5_NN and 6 ½ B were then ligated into linker 5-6 to create 5B_NN |
| Linker 5-6 ½ Rev | CAG GTC TCA CGC TCC CCC TCA AGA GAC CCG | Used to create Linker 5-6 in pUC57 with two BsaI sites. Repeats 5_NN and 6 ½ B were then ligated into linker 5-6 to create 5B_NN |
| Toolkit D ½ BpiI Rev | GGG GAA GAC CCT AAC CCC GCA GCA GGT GG | Used to create flexible *TALE* toolkit half repeat modules with the D overlap. |
| pUC57 ½ BpiI Rev | CCC GAA GAC CCA GCG CCG GCC TGC | Used to create flexible *TALE* toolkit half repeat modules with the D overlap. |
| Rep7_D-overlap_Fwd | TAA CTG AGA CCT GGG CCC GTC GAC TGC AG | Used to create modified *TALE* toolkit repeat 7D_NS |
| Rep7_D-overlap_Rev | GGC CAT GGG CCT GGC ACA GCA CCG | Used to create modified *TALE* toolkit repeat 7D_NS |
| pVAX GoldenGate + Sp6 Fwd | ATC AAT GTG AGA CCT TTC CCG GGT TTG GTC TCT GCT TGG GCC CGT TTA AAC CCG CTG ATC AG | Used to remove the previous TALEN gene from a published TALEN expression vector (18), replace it with BsaI sites and introduce an Sp6 priming site into the CMV promoter. |
| pVAX GoldenGate + Sp6 Rev | ATC ACT AGC TTC TAT AGT GTC ACC TAA ATC AGC TTG AGT CTC CCT ATA GTG AGT CG | Used to remove the previous TALEN gene from a published TALEN expression vector (18), replace it with BsaI sites and introduce an Sp6 priming site into the CMV promoter. |
| HA-NLS GoldenGate AATG Fwd | TTG GTC TCT AAT GGG CTA CCC TTA CGA CGT GC | Used to amplify HA-NLS domain from a published TALEN construct (18) and introduce BsaI sites. |
| HA-NLS GoldenGate TATG Rev | AAT GGT CTC ACA TAG CGT GGA TGC CCA CTT TCC GC | Used to amplify HA-NLS domain from a published TALEN construct (18) and introduce BsaI sites. |
| 3xHA goldengate Fwd | TTT GGT CTC TAA TGG GGT TAA TTA ACA TCT TTT ACC CAT ACG | Used to amplify 3xHA from binary vector pGWB13 (37) and introduce BsaI sites |
| 3xHA goldengate Rev | TTT GGT CTC ACA TAC CGC TGC ACT GAG CAG CGT AAT C | Used to amplify 3xHA from binary vector pGWB13 (37) and introduce BsaI sites |
| FokI GGTG BpiI Fwd | TTT GGT CTC TGG TGG TCA GCT AGT GAA ATC TGA ATT GGA AGA G | Used to amplify FokI nuclease domain from a published TALEN construct (18) and introduce BsaI sites. |
| FokI GGTG BpiI Rev | AAT GGT CTC AAA GCT TAT CTC ACC GTT ATT AAA TTT CCT TCT CAC | Used to amplify FokI nuclease domain from a published TALEN construct (18) and introduce BsaI sites. |
| *Bat1*_Block 1 TATG Rev | CAT AAG AGA CCA TTG GGA TCG GAT C | Used to modify ‘Bat1 Block1’ (Figure S4) for cloning into the pVAX derived human cell expression vector and remove start codon (provided by N-terminal tag). |
| *Bat1*_Block 1 ATGless Fwd | AGC ACC GCC TTC GTG GAC CAA G | 5’ Phosphorylated. Used to modify ‘Bat1 Block1’ (Figure S4) for cloning into the pVAX derived human cell expression vector and remove start codon (provided by N-terminal tag). |
| Block 5 GGTG Fwd phospho | GGT GTG AGA CCG ACC CAA TAT C | 5’ Phosphorylated. Used to modify ‘Bat1 Block5’ (Figure S5) to remove stop codon and for cloning into the pVAX derived human cell expression vector. |
| Block5 Last codon Rev | CTG ACG TTC CAG CAG CGG AG | Used to modify ‘Bat1 Block5’ (Figure S5) to remove stop codon and for cloning into the pVAX derived human cell expression vector. |
| acBat1 AD out Rev phospho | GCT GGC CTC CAC CTT TCT C | Used to remove VP64 activation domain from acBat1 C-terminal domain. |
| acBat1 AD out Fwd | TAG GCT TTG AGA CCA CGA AG | Used to remove VP64 activation domain from acBat1 C-terminal domain. |
| acBat1 NLS out Rev | CTT GTC ATC GTC ATC CTT GTA GTC | Used to remove the NLS from the acBat1 C-terminal domain. |
| acBat1 NLS out Fwd | GGT TCC GGA CGG GCT GAC | 5’ phosphorylated. Used to remove the NLS from the acBat1 C-terminal domain. |
| BAT1rep20 2^nd^ Helix Fwd | GCC CTG AAA GCT GTC CTG AAG TAT G | Used to create acBat1Δ18-20 and acBat1Δ16-20 |
| BAT1rep20 GG Fwd | GGC GGT GCG CAG GCC CTG AAA GCT GTC CTG AAG | Used to create acBat1Δ14-20 and acBat1Δ12-20 |
| BAT1 rep18 1st Helix Rev | CTG GGC ACC ACC ACC ATT ACC CGC | Used to create acBat1Δ18-20 |
| BAT1 rep16 1st Helix Rev | CTG CGC ACC GCC ATT GTT GCC AGC AAC GTT C | Used to create acBat1Δ16-20 |
| BAT1 rep14 1st Helix Rev | GCT ATT GCC CGC GAT TTT CAC GAT GTC CGG TTG | Used to create acBat1Δ14-20 |
| BAT1 rep12 1st Helix Rev | GGT GTT ACC GGC GAT TTT GAC AAT ATC CGG CTG | Used to create acBat1Δ12-20 |
| Bat1 NTD out Fwd | TTT AGC CAG TCT GAC ATT GTC AAG ATC GC | 5’ phosphorylated. Used to create acBat1ΔNTD |
| Bat1 NTD out Rev | CAT AAG AGA CCA TTG GGA TCG GAT C | Used to create acBat1ΔNTD |
| Bat1 CTD out Fwd | AAG GTG AGA CCG ACC CAA TAT C | 5’ phosphorylated. Used to create acBat1ΔCTD |
| Bat1 CTD out Rev | ACC TGC TTG CAT CAG CAC CG | Used to create acBat1ΔCTD |
| BE_Bat1_ into Bs3p Fwd | TGC TTC TCT TAG TTG TGA GGA TGG TTA GG | 5’ Phosphorylated. Used to create Bs3p BE_Bat1_ for GUS assays and for the creation of the Bat1-Fok1 target templates. |
| BE_Bat1_ into Bs3p Rev | AAGACGTTAGGTTCAAGTTATCATCCCC | Used to create Bs3p BE_Bat1_ for GUS assays and for the creation of the Bat1-Fok1 target templates. |
| Bat1-Fok1 target 5bp | CTA GCA TAA CGT CTT TGC TTC TCT TAG | Used to create the 5bp spacer target for the nuclease assays. |
| Bat1-Fok1 target 7bp | TCT AGA CAT AAC GTC TT GCT TCT C | Used to create the 7bp spacer target for the nuclease assays. |
| Bat1-Fok1 target 11bp | TAC GTC TAG ACA TAA CGT CTT TGC TTC TC | Used to create the 11bp spacer target for the nuclease assays. |
| Bat1-Fok1 target 15bp | TAC GTA CGT CTA GAC ATA ACG TCT TTG CTT CTC | Used to create the 15bp spacer target for the nuclease assays. |
| Bat1-Fok1 target 19bp | TAA GCT ACG TAC GTC TAG ACA TAA CGT C | Used to create the 19bp spacer target for the nuclease assays. |
| BE _Bat1_ TAGA Fwd | TAG ACT AAG AGA AGC AAA GAC GTT ATA TGC | To get BE_Bat1_ into dsEGFP reporter |
| BE _Bat1_ CCTA Rev | ATC CGC ATA TAA CGT CTT TGC TTC TCT TAG | To get BE_Bat1_ into dsEGFP reporter |

| **Table S3: p-values for two-tailed t-tests without assuming equal variances to establish whether affinities differ between interactions of Bat1 with BE_Bat1_ derivatives bearing A, C, G or T at the zero position.** | | | | |
| --- | --- | --- | --- | --- |
|  | **A_0_** | **C_0_** | **G_0_** | **T_0_** |
| **A_0_** |  |  |  |  |
| **C_0_** | **0.589** |  |  |  |
| **G_0_** | **0.860** | **0.860** |  |  |
| **T_0_** | **0.231** | **0.382** | **0.754** |  |
| Sample size n=3. (A_0_, G_0_, T_0_) or 5 (C_0_). Results shown to three decimal places. | | | | |

| **Table S4: Hydrogen bonds formed between repeat residues of Bat1 predicted with UCSF Chimera (39). Unless stated, interactions are between side chain and backbone atoms.** | | | |
| --- | --- | --- | --- |
| **Repeats involved** | **AA 1** | **AA 2** | **Comment** |
| -1 – 0 | Gln 29 | Ala 59 |  |
| 0 – 1 | Lys 57 | Gly 93 |  |
| 0 - 1 | Tyr 61 | Ala 92 |  |
| 1 – 2 | Gly 82 | Arg 118 | In the inter repeat loop region |
| 3 – 4 | Asn 160 | Ala 191 |  |
| 3 – 4 | Gly 162 | Thr 194 |  |
| 2 – 4 | Gly 148 | Arg 184 | In the inter repeat loop region |
| 4 – 5 | Thr 202 | His 234 |  |
| 5 – 6 | Lys 222 | Gly 258 |  |
| 6 – 7 | Asn 292 | Ala 323 |  |
| 7 – 8 | Asn 325 | Gly 357 |  |
| 7 – 8 | Gln 349 | Arg 343 | In the inter repeat loop region |
| 7 - 8 | Asn 326 (N) | Asp 359 |  |
| 8 – 9 | Asn 358 | Gly 390 |  |
| 8 – 9 | Asn 358 | Ala 389 |  |
| 11 – 12 | Lys 420 | Gly 456 |  |
| 11 – 12 | Arg 442 | Phe 446 (N) | Inter repeat connection |
| 11 – 13 | Arg 444 | Ser 480 (OH) |  |
| 13 – 14 | Arg 510 | Leu 534 (O) |  |
| 13 – 14 | Arg 491 | Ser 524 |  |
| 15 – 16 | Asn 556 | Gly 588 |  |
| 16 – 17 | Asn 589 | Gly 621 |  |
| 16 – 17 | Glu 601 | Lys 630 |  |
| 17 – 18 | Asp 615 | Arg 646 | Between two side chains |
| 17 – 18 | Glu 634 | Lys 663 | Between two side chains |
| 19 – 20 | Glu 700 | Lys 728 | Between two side chains |
| 20 - +1 | Asn 721 | Arg 754 (O) |  |

36. Szurek, B., Marois, E., Bonas, U., Van den Ackerveken, G. (2001) Eukaryotic features of the Xanthomonas type III effector AvrBs3: protein domains involved in transcriptional activation and the interaction with nuclear import receptors from pepper. Plant J., 26, 523-534.

37. Nakagawa, T., Kurose, T., Hino, K., Tanaka, K., Kawamukai, M., Niwa, Y., Toyooka, K., Matsuoka, K., Jinbo, T., Kimuraf, T. (2007) Development of series of gateway binary vectors, pGWBs, for realizing efficient construction of fusion genes for plant transformation. J. Biosci. Bioeng., 104, 34-41.

38. Schwede, T., Kopp, J., Guex, N. and Peitsch, M.C. (2003) SWISS-MODEL: an automated protein homology-modeling server. Nucleic Acids Res., 31,3381-3385.

39. Pettersen, E., Goddard, T., Huang, C., Couch, G., Greenblatt, D., Meng, E., Ferrin, T. (2004) UCSF Chimera - a visualization system for exploratory research and analysis. J. Comput. Chem., 25,1605-1612.
